# Are hummingbirds generalists or specialists? Using network analysis to explore the mechanisms influencing their interaction with nectar resources

**DOI:** 10.1371/journal.pone.0211855

**Published:** 2019-02-27

**Authors:** Claudia I. Rodríguez-Flores, Juan Francisco Ornelas, Susan Wethington, María del Coro Arizmendi

**Affiliations:** 1 Ecology Laboratory (Laboratorio de Ecología), Biotechnology and Prototype Unit (Unidad de Biotecnología y Prototipos [UBIPRO]), Iztacala Faculty of Higher Studies (Facultad de Estudios Superiores Iztacala), National Autonomous University of Mexico (Universidad Nacional Autónoma de México), Los Reyes Iztacala, Tlalnepantla de Baz, Estado de México, México; 2 Biological Sciences Postgraduate Program (Posgrado en Ciencias Biológicas), National Autonomous University of Mexico (Universidad Nacional Autónoma de México), Posgraduate Unit (Unidad de Posgrados), Ciudad de México, México; 3 Department of Evolutionary Biology (Departamento de Biología Evolutiva), Institute of Ecology (Instituto de Ecología, A.C.), Xalapa, Veracruz, Mexico; 4 The Hummingbird Monitoring Network, Patagonia, Arizona, United States of America; University of New England, AUSTRALIA

## Abstract

Mutualistic interactions are powerful drivers of biodiversity on Earth that can be represented as complex interaction networks that vary in connection pattern and intensity. One of the most fascinating mutualisms is the interaction between hummingbirds and the plants they visit. We conducted an exhaustive search for articles, theses, reports, and personal communications with researchers (unpublished data) documenting hummingbird visits to flowers of nectar-rewarding plants. Based on information gathered from 4532 interactions between 292 hummingbird species and 1287 plant species, we built an interaction network between nine hummingbird clades and 100 plant families used by hummingbirds as nectar resources at a continental scale. We explored the network architecture, including phylogenetic, morphological, biogeographical, and distributional information. As expected, the network between hummingbirds and their nectar plants was heterogeneous and nested, but not modular. When we incorporated ecological and historical information in the network nodes, we found a generalization gradient in hummingbird morphology and interaction patterns. The hummingbird clades that most recently diversified in North America acted as generalist nodes and visited flowers with ornithophilous, intermediate and non-ornithophilous morphologies, connecting a high diversity of plant families. This pattern was favored by intermediate morphologies (bill, wing, and body size) and by the low niche conservatism in these clades compared to the oldest clades that diversified in South America. Our work is the first effort exploring the hummingbird-plant mutualistic network at a continental scale using hummingbird clades and plant families as nodes, offering an alternative approach to exploring the ecological and evolutionary factors that explain plant-animal interactions at a large scale.

## Introduction

Mutualisms, such as seed dispersal and pollination, are key factors explaining the distribution and persistence of biodiversity on Earth [1]. Notably, mutualisms create complex networks of interacting species whose relationships vary in type, strength, and duration [2, 3]. Interacting species impose reciprocal selective pressures on each other as they interact over ecological and evolutionary time. The properties of the resulting interaction networks can help to describe and explain current biodiversity patterns [3].

**Fig 1:**
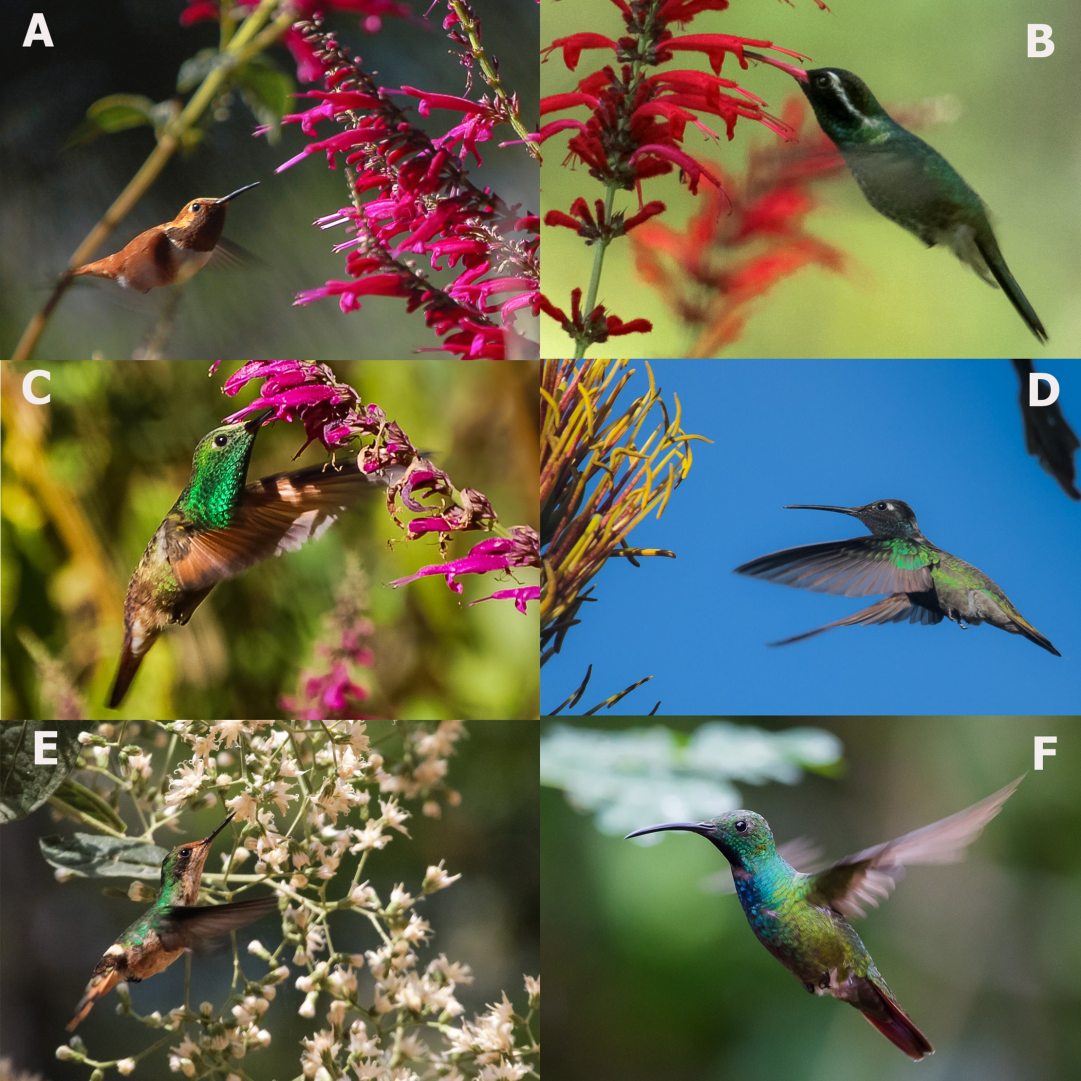
Photographs of hummingbirds and their nectar resources. *Salvia iodantha* (Lamiaceae) flowers are visited by long-distance seasonal migrant species including *Selasphorus rufus* (Bee clade, A), and resident hummingbirds *Hylocharis leucotis* (B) and *Amazilia beryllina* (C) of the Emerald clade at the temperate forests of the Sierra de Manantlán, Jalisco, Mexico. D. *Eugenes fulgens* (Mountain Gems clade), a large altitudinal migrant, visits flowers with different morphologies including those of Agave species (Asparagaceae). E. *Lophornis brachylophus* (Coquette clade) visits the flowers of many non-ornithophilous plant species as *Vernonatura cordata* (Asteraceae). This coquette is an endemic, range-restricted species and endangered by habitat destruction. F. *Anthracothorax prevostii* (Mangoes clade) is a widely distributed species in the lowlands with a curved bill that can be easily identified by its brilliant colors. Photographs by Carlos Soberanes-González.

Hummingbirds (Aves: Trochilidae) are specialized nectarivorous birds that act as pollinators, transporting gametes among flowers [4]. The hummingbird diversity pattern shows a strong latitudinal gradient throughout the Americas [5, 6]. Hummingbirds colonized South America approximately 22 million years ago, yet their center of diversification, as revealed by molecular phylogenetics, is in the Neotropics [7, 8]. In particular, hummingbirds diversified into nine clades, some of which had a rapid rate of species diversification as a result of adaptation to new ecological niches and dispersion to new geographical areas [9]. Hummingbirds morphology and ecology (including relationships with flowers) reflect phylogeny, and hummingbird clades have characteristic morphologies that influence resource use, flight capabilities, competitive skills and environmental filtering, important mechanisms structuring hummingbird communities (Fig 1) [10, 11].

Theoretical predictions about the factors influencing the interaction between hummingbirds and the plants they use as nectar resources can be tested based on the biogeography of hummingbirds and plants as well as the ecology of hummingbird and plant interactions. The pollination system resulting from the co-evolution of hummingbirds and plants in the Western Hemisphere can be further described using network properties.

Examining 31 plant-hummingbird networks, Dalsgaard *et al*. [12] found that biotic specialization decreases with decreasing latitude and that hummingbird species richness, contemporary precipitation, and Quaternary climate-change velocity were key predictors of biotic specialization. Similarly, Martín-González *et al*. [13] investigated hummingbird-plant diversity patterns and found a significant association between network structure (complementary specialization and modularity) and species’ phylogenetic signals at a macroecological scale, suggesting a close co-evolutionary association between hummingbirds and their nectar plants. Also, Vitória *et al*. [14] studied how species’ evolutionary histories shaped the interaction network between hummingbirds and plants in the Atlantic Forest in Brazil and concluded that morphology and phenological matching among species were more important than evolutionary history in structuring the studied plant-hummingbird network.

The hemispheric scale of interactions between hummingbirds and their nectar plants also allow comparisons between niche conservatism versus niche evolution theories. The range of environmental conditions that are inhabitable by species, enabling them to maintain viable populations, comprises the fundamental niche [15]. Niche conservatism occurs when the colonization and dispersion of species are limited as a result of environmental changes affecting their ancestral niche. Conversely, niche evolution occurs when species disperse and colonize new habitats with environmental conditions differing from those of their ancestral fundamental niche [15]. Based on environmental conditions and species diversity patterns in 188 New World vertebrate families that originated in North and South America (including hummingbirds), Smith *et al*. [16] found that many families with a southern origin exhibited niche conservatism and had lineages restricted to Neotropical areas, whereas many families with a northern origin were distributed across Nearctic and Neotropical areas. These results suggest that northern lineages have contributed more to high tropical biodiversity than southern lineages have contributed to northern temperate biodiversity [16].

Accordingly, hummingbird diversity reflects both niche evolution and niche conservatism patterns. For example, hummingbird diversity in the Neotropics is the highest in moist cloud forests at middle elevations [7, 17], where many species and clades (i.e., Brilliants) have limited ranges [17], reflecting niche conservatism. Conversely, the evolutionary history of Bee Hummingbirds, the most northern, most recent hummingbird clade with the highest rate of net diversification [9, 18], suggests a release of niche conservatism constraints, resulting in niche evolution. Licona-Vera and Ornelas [18] explored the phylogenetic and biogeographical relationship among Bee Hummingbirds (Mellisugini) and their nectar plants to assess the evolution of long distance seasonal migration in this clade. Their results suggest that the historical range expansion of this clade is connected with the biogeography of their host plants and the colonization of new ecological niches. Along these same lines, Abrahamczyk & Renner [19] found that diversification within hummingbird-pollinated clades in temperate North America and South America regions was a gradual and continuous process due to habitat specialization and allopatric speciation in which independent plant species switched from insect to hummingbird pollination.

However, integrating the historical biogeography and ecology of hummingbird clades and their nectar resources remains largely unexplored at the hemispheric scale. [20]. Previous studies have examined mutualistic networks based on community and site records. We aimed to construct a mutualistic network between the nine hummingbird clades and their nectar plant families based on records of hummingbird visits to plants. Moreover, we aimed to combine phylogenetic, morphological, latitudinal, elevational, and biogeographical information in order to identify the ecological and evolutionary mechanisms driving the interaction and specialization between hummingbirds and plants used by hummingbirds as nectar resources. Some researchers have highlighted the need to understand the ecological, evolutionary, and historical mechanisms explaining the architecture of mutualistic networks and to compare these interactions at different scales [21, 22]. We opted to use hummingbird clades because the limits between these clades are clear in terms of phylogeny, ecology, and evolution, although the placement of genus *Patagonia* is still controversial. Overall, each hummingbird clade has a unique evolutionary history that is reflected in their current distribution, morphology, and ecology [7, 9].

As the plant-hummingbird network is based on mutualistic interactions (i.e. interactions involving mutual benefits among partners [23] (Fig 1), we predicted that the presently analyzed network would behave like previously described mutualistic networks. In particular, (1) we expected the connectivity distribution to be highly heterogeneous because most hummingbird species visit few plant species, and few hummingbird species are more connected than expected by chance. Also, (2) we expected the network to be nested because hummingbird and plant specialist nodes typically interact with subsets of species that are mainly connected with generalist nodes. Finally, (3) we expected the network to be modular because some species subgroups tend to interact more strongly among themselves than with other species [1, 23–25].

To explore the connection pattern between hummingbirds and their nectar resources from an evolutionary and geographical perspective, we tested the following two hypotheses using phylogenetic, biogeographical, morphological, and distributional information. First, we posited that biogeographical evolution acting along with hummingbird clade diversification [9, 26] has affected species co-occurrences and, consequently, has influenced life-history traits of birds and plants [3, 19]. On the basis that a monophyletic ancestor of hummingbirds from tropical South America later colonized Andean South America (Brilliant and Coquette clades), the Caribbean, and North America (Mango, Bee, and Mountain Gem clades) [9], we hypothesized that there is a morphological trend toward generalization among recent colonizing lineages wherein morphologies tend to facilitate access to nectar from a higher diversity of flower resources [9, 19, 27]. Specifically, we expected that hummingbird species in generalist nodes would have small- to medium-sized (intermediate length) straight bills. With respect to plants, we expected that flowers visited by generalist hummingbirds would have more varied morphologies, from typically ornithophilous flowers to more entomophilous ones (*sensu* Faegri & Van der Pijl [28]). Secondly, taking into account that biogeographical patterns are the result of ecological and environmental processes that influence colonization and dispersion at different ecological and spatial scales [15], we expected that hummingbird clades with a center of diversification in North America (i.e., those with lower niche conservatism) would have wider elevational and latitudinal ranges than those that mainly diversified in South America [29, 30], thus favoring the colonization of South America by northern hummingbird lineages. Finally, we expected that northern lineages (Bee and Mountain Gem clades) would interact with more plant families.

In the present paper, we first describe the mutualistic network of the hummingbird clades and plant families used as nectar sources at a continental scale, and then explore the factors explaining the connection pattern from an ecological and evolutionary perspective. We found that the evolution of clades, hummingbird and plant morphology, biogeographical region, and the center of diversification were the factors that best explained the patterns in this network.

## Materials and methods

### Ethics statement

An ethics statement is not required for the present study. No specific permits were required for the described methodology. Researchers who shared unpublished data from local studies had the full knowledge that these data would be used to analyze the geographical patterns of mutualistic networks. Our field studies did not involve endangered or protected species, and hummingbird morphological measurements were mainly obtained by JFO from museum specimens (see Acknowledgments). No live animals were manipulated.

### Hummingbird-plant interactions

#### Data collection

We compiled data for hummingbirds and their nectar plants conducting exhaustive searches in academic databases, search engines, and online university libraries using the following key words: “hummingbird,” “Trochilidae,” “flower,” “visit,” and “pollination.” Additionally, we obtained information from the bird and specialized hummingbird literature, including unpublished theses, records, and personal communications from experts.

For hummingbirds, we followed the nomenclature of the South American Classification Committee (SACC) [31] and the American Ornithologists’ Union (AOU) [32] and, for plants, we followed the nomenclature of TROPICOS [33] and the World Checklist of Selected Plant Families [34]. For plants, we determined the category of native vs. non-native using the distribution maps in TROPICOS [33] and JSTOR Plant Science [35].

#### Network matrix

Using the compiled information, we constructed a qualitative matrix with hummingbird species in the columns and plant species in the rows. In this binary matrix, 1 indicates an interaction between a hummingbird and a plant species, and 0 otherwise. We discarded the option of using a quantitative matrix because of the high variability in the methods used by the different sources (in terms of sampling effort, time lag, and area sampled, for example), which could affect the observed pattern and subsequent interpretations [36]. Also, in most cases, the authors did not provide an estimate of the number of interaction events. We constructed a second matrix using the same principles described above but excluding the plant species that were non-native to the New World. We conducted all network analyses with and without non-native plant species to minimize the potential confounding effects of non-native plant species on the evolutionary patterns detected in the network.

Because we were interested in mutualistic relationships at the global level, we grouped hummingbird species by clade or lineage according to McGuire *et al*. [7] and plant species by family. Mutualistic networks were graphed using the “plotweb” and “visweb” functions in the bipartite package [37] in R version 3.1.2 [38] in conjunction with pajek [39].

#### Hummingbird and plant phylogeny

To explore whether the architecture of the mutualistic network corresponded with the evolutionary history of the nodes, we built the phylogenies for the nodes included in the network and coupled these with the mutualistic network. This comparison allowed us to detect correspondences in an ecological and evolutionary context. Plant phylogeny was built at the family level using Phylomatic [40] and following Davies *et al*. [41], and hummingbird phylogeny was drawn using the ape package [42] in R version 3.1.2 [38] following the codes used by McGuire *et al*. [7] for hummingbird clade classification.

### Network analysis

#### Connectivity distribution

Connectivity distribution is a measurement of network robustness or resiliency that describes how interactions are distributed across nodes [4]. Species phenology and morphological co-adaptations can operate as constraints that prevent or favor the occurrence of network interactions [4, 28, 29]. In mutualistic networks, the frequency distribution of the number of interactions per node is heterogeneous; in other words, the number of interactions across nodes varies more than expected by chance [23]. In these networks, a few nodes have a large number of connections (generalist nodes) in comparison to the bulk of the nodes, which have few connections (specialist nodes) [23]. Using matrices with and without non-native plant species, we calculated the cumulative frequency of the interactions between all species in the network. We tested whether the observed probability fit one of three different distributions: (1) exponential, (2) power-law, or (3) truncated power-law [30, 43–45]. Adjustment to a particular distribution provides information about the scale of a network and its resistance to node loss [23, 30]. Mutualistic networks adjusted to the power-law distribution have a scale-free degree distribution. These networks are robust in face of random node extinction but are fragile to extinctions at the most connected nodes [23]. We obtained the Akaike Information Criterion (AIC) for each fit and chose the fit with the lowest value as the best fit [44]. Statistical tests for the AIC and the distribution comparisons were performed using the “brainwaver” package [46] in R version 3.1.2 [38].

#### Nestedness

Nestedness is a property concerning the pattern of connections in a network given the identity of the nodes [23]. In a nested network, specialist nodes interact with certain species that form perfect subsets of species with which generalist nodes interact, offering robustness and tolerance to node loss [30, 47]. Node abundance, phenotypic complementarity, and phylogenetic history have been proposed as factors underlying a nested pattern and have important consequences for how co-evolution acts on mutualistic interactions [48–50]. We estimated matrix nestedness using a Nestedness metric based on Overlap and Decreasing Fill (NODF) [51]. Perfectly nested networks have high values of NODF, wherein 0 indicates a compartmentalized matrix and 100 a perfectly nested matrix [51]. The ANINHADO software [52], and the vegan [53] and Metacom [54] packages in R version 3.1.2 [38] were used to estimate the NODF values and to test whether the degree of nestedness departed statistically from the random expectation. The choice of the correct null models is crucial, and because all null models have pros and cons, the best strategy is to select a suite of null models that allows identifying the ecological factors that structure the network [23, 55]. We selected three null models that differ in the way they are constrained: Erdos-Renyi (ER), CE and fixed-fixed (FF) [1, 52, 55]. The ER model is the least constrained binary null model where the “1s” in the original matrix were randomly assigned; in other words, each cell in the interaction matrix had the same probability of being occupied. This null model is prone to Type I error detecting nestedness when a matrix is random [55]. In the CE model, the probability of having an interaction in the simulated matrix was estimated as the arithmetic mean of the connection probabilities of the focal plant and animal species [52]; in biological terms, this probability was proportional to the level of generalization of plants and animals in the original matrix [1, 52]. CE model offers and unbiased estimates of overall connectivity and had a low rate of Type I and II errors [23]. Finally, the FF model is the most constrained null model, and it constrains matrix size, marginal totals, and frequency. These constraints favor a more conservative model where the more elements of the original matrix are incorporated, decreases the occurrence of Type I error, but is prone to Type II error [56]. We generated 3000 random nets, or 1000 for each null model. Because we used a phylogenetic approach for network construction and data interpretation, we also calculated the NODF and generated 3000 random nets (1000 for the ER model, 1000 for the CE model, and 1000 for the FF model) using the non-ordered option in the ANINHADO software [52], and “bipartite” [57] and “metacom” [54] packages. We requested that the program uses the phylogenetic order provided by us to calculate nestedness (see Hummingbird and plant phylogeny section above). For each null model we estimated the arithmetic mean of the 1000 random networks and the standardized effect size (SES). SES was calculated as the difference between the observed values and the mean of the simulated values, divided by the standard deviation of the simulated values.

#### Modularity

Modularity describes the degree to which a network is organized in compartments or subsets of nodes that are highly connected and interact more frequently with each other, influencing network stability and persistence [24,58]. Phenological and morphological complementary are two mechanisms acting in a modular network that define the role of each node and that connect nodes within and/or between modules [58]. To explore whether the hummingbird-plant network was modular, we used an algorithm based on simulated annealing (SA) implemented in the NETCARTO program [59,60]. This algorithm was designed for unipartite networks, enabling us (1) to measure the degree to which the network was organized into clearly defined modules by calculating an index of modularity (M) for each network, (2) to identify the number of modules within the network and the number of nodes belonging to each module, and (3) to assign a role to each node according to its topological properties [58, 61–63].

For the hummingbird-plant network, we examined whether the obtained modularity index was significantly more modular than the modularity index calculated for 200 random networks (100 for ER model and 100 for FF model; [63]). Additionally, for ER and FF null models we estimated the arithmetic mean and SES as described above. Because the results of the SA algorithm may vary in different runs, we ran the modularity analysis 50 times and calculated the mean and standard deviation. Species were included in a particular module if they were assigned to the module in > 90% of the runs [64].

We characterized each node in the network based on its topological properties. Specifically, each node was characterized by comparing its position with that of other nodes in its own module, *z* (standardized within-module degree) and how well it connects to nodes in other modules using *c* (among-module connectivity), and its placement in the *zc*-parameter space [58]. Following Olesen *et al*. [58], we sorted all species into four roles using *z* = 2.5 and *c* = 0.62 as cutoff values. A *peripheral* node has few links inside its own module but rarely with other modules (*z* ≤ 2.5 and *c* ≤ 0.62). A *connector* node links different modules (*z* ≤ 2.5 and *c* ≥ 0.62). A *module hub* maintains the coherence of its own module (*z* ≥ 2.5 and *c* ≤ 0.62). And, a *network hub* is a “supergeneralist” node that maintains the coherence of both the network and its own module (*z* ≥ 2.5 and *c* ≥ 0.62). Furthermore, we tested the suitability of these cutoffs exploring if the location of each node in the *zc-*space agreed with the theoretical role in the network, and analyzing the behavior of *z* and *c* values when the number of internal and external links in the modules changed [58]. We are aware about the limitations of modularity estimates in qualitative networks, and the recent advances in the establishment of efficient algorithms to identify modules in quantitative bipartite networks using null models [37]. However, the algorithm used here performed adequately identifying modularity as those with similar algorithms for binary qualitative matrices [63].

### Morphological information

To explore the relationship between morphology and network architecture (in terms of connectivity, nestedness, and modularity), we examined morphological information for the flowers and hummingbirds included in our network. For plants, we used the classical pollination syndromes with modifications [65]. We detected three main morphological groups in the flowers visited by hummingbirds and combined some of the traits used by Faegri & van der Pijl [65], Proctor & Yeo [28], Rocca & Sazima [66], Thomson *et al*. [67], and Wilson *et al*. [68] to describe these groups. More specifically, we classified each species, genus, and family as ornithophilous, intermediate, or non-ornithophilous and then established the most common characteristics of these morphologies (see Table 1 for flower characteristics). This classification was exclusively made for the plant records included in this study. Ollerton *et al*. [69] expressed caution over the use of pollination syndromes because they may inadequately describe the diversity of floral phenotypes or predict the most common pollinators. We are aware of these limitations, but for the hummingbird-plant matrices, we are not interested in making inferences about the pollinators but rather in having a conceptual framework for characterizing the morphological traits of the flowers that hummingbirds visit.

**Table 1:** Floral morphology classification.

Ornithophilous flower*	Intermediate flower**	Non-ornithophilous flower***
Diurnal anthesis.	Diurnal and/or nocturnal anthesis.	Diurnal and/or nocturnal anthesis.
Corolla with vivid colors, often scarlet, red, or orange (very rarely purple). If corolla has non-vivid colors (yellow or white), it strongly contrasts with the calyx and/or floral bracts.	Corolla yellow, white, blue-violet, or purple (very rarely red or orange), but with contrasting patterns.	Generally non-vivid colors (principally yellow and white), rarely purple or orange corollas, but without strong contrast.
Lip or margin absent (if present, curved backwards), flower hanging with inclined orientation, zygomorphy frequent, less pronounced landing platform.	Margin generally very expanded, pronounced landing platform. Actinomorphy and zygomorphy may be present. Flowers have different orientations.	Actinomorphic flowers, principally oriented upwards. If margin or lip present, it forms a landing platform.
Pedicel may be elongated, and pedicel and inflorescence axis may be delicate.	Pedicel and inflorescence axis robust or delicate.	Pedicel short. Pedicel and inflorescence axis robust.
Exerted anthers and stigma. Pollen load deposited with precision on the pollinator.	Pollen load deposited with precision on pollinators but more dispersed compared with ornithophilous morphologies.	Exposed reproductive organs. Pollen load deposited more dispersedly on pollinators.
Mainly tubular and bilabiate flowers. Deep tube or spur, wider than intermediate flowers (wide enough to allow the hummingbirds’ beak to effectively enter the corolla tube).	Urceolate, funnelform, and salverform flowers.	Principally rotate- and brush-shaped flowers. If campanulate, generally shallow.

*Flowers pollinated by hummingbirds described by Faegri & van der Pijl [65], Proctor & Yeo [28], Thomson *et al*. [67], and Wilson *et al*. [68] with modifications.

** Flowers pollinated by moths, butterflies, and bats described by Faegri & van der Pijl [65] and Proctor & Yeo [28] with modifications.

*** Flowers pollinated by perching birds and/or bees described by Rocca & Sazima [66] and Faegri & van der Pijl [65] with modifications.

For hummingbirds, we used information previously collected by Núñez-Rosas *et al*. [70], J. Hernández (pers. comm.), JFO, and Schuchmann [4] to calculate the mean and standard deviation of the body weight, wing chord, and bill length (exposed culmen) of 292 hummingbird species (around 89% of all extant species). We further classified these species by bill curvature: recurved, straight, curved, or strongly curved. We used these trait categories because they provide information about the size of hummingbirds and traits are associated with their access to and use of nectar resources [71, 72].

### Biogeographical distribution and center of diversification

To assess the biogeographical distribution of hummingbird species, we calculated the latitudinal and elevational gradient for the hummingbird species included in the matrix and counted the number of biogeographical regions in each species’ range.

To define whether each species was distributed in the Nearctic, Neotropical, and/or Austral region, we used the range maps from ebird [73] and del Hoyo *et al*. [74]. We estimated the total number of species in each clade and genus occurring in each of these regions. We defined the Nearctic, Neotropical, and Austral biogeographical regions according to the Sclater-Wallace system [16, 75] considering the modifications for bird genera proposed by Rueda *et al*. [75]. The Nearctic region extends from Alaska to the Trans-Mexican Volcanic Belt (around 19° to 20° N). The Neotropical region includes the tropical lowlands adjacent to the Mexican highlands and extends southwards, including the northern and central portions of South America. Finally, the Austral region comprises the southern Andes from Peru to the Patagonia (Fig 1C in Rueda *et al*. [75]).

For the latitudinal range, we used unpublished data from Ornelas [76]. We established the northernmost and southernmost range limits for each hummingbird species and calculated the mean latitudinal range for each genus and clade. To obtain distributional information for plants, we consulted distribution maps from TROPICOS [33], The Plant List [77], and the International Union for Conservation of Nature’s Red List of Threatened Species [78].

For the elevational distribution, we defined the elevational range of each species as the difference between the minimum and maximum elevation where each species has been recorded, excluding all records referred as “possible” or “rarely”. Then, we estimated the mean elevational range for each clade. We calculated these ranges for hummingbird species using information from Arizmendi *et al*. [79], Bleiweiss [80], Ornelas [76], and Schuchmann [4].

We assigned the center of diversification to each hummingbird species based on published studies on molecular phylogenetics, species diversification, and ancestral biogeographical inferences for hummingbirds [7, 9, 81]. The two geographical areas were North America (from Alaska to the Isthmus of Panama) and South America following Smith *et al*. [16].

We only considered mainland species, excluding hummingbird species from the Caribbean and other oceanic islands, because hummingbirds are principally a mainland avian group with less than 5% of species inhabiting islands. Besides, phylogenetic relationships between hummingbird insular species, specially the relationships between migrant and resident species, are still controversial making the inference about the center of diversification difficult [9, 18]. Finally, the complexity and uniqueness of the Caribbean region in terms of geological history, climate, and biogeography [82], makes this region difficult to classify in terms of biogeographical regions, being included interchangeably in the Neotropical or Nearctic region depending on the classification and focal study group [75].

### Hummingbird niche conservatism analysis

To test whether niche conservatism in hummingbirds was related with the center of diversification and/or clade identity, we fitted Generalized Linear Models (GLM) with binomial distribution to logit link and probit link functions, respectively [83]. We defined species distributed in two or three of the biogeographical regions (see above) as a success (corresponding with a value of 1 for the binomial response variable) and species distributed in only one of these as a failure (corresponding with a value of 0 for the binomial response variable). The independent variables were center of diversification (categorical variable with two levels) and clade (categorical variable with 8 levels). In these models, we excluded the Patagona clade because it has only one species (*Patagona gigas*).

We tested for overdispersion using the protocol proposed by Zuur, Ieno, and Elphick [84]. All analyses were performed in R software version 3.3.0 [85]. The model selection process was performed using the “base” (R Development Core Team, 2014) and “car” packages [86]. Post-hoc multiple comparison tests of the different fitted models were carried out using the general linear hypothesis function (glht) in the “multcomp” package [87]. We used the Bonferroni correction for multiple testing and an alpha of 0.05 or less to determine significance. Plots were made using the “ggplot2” package [88].

## Results

### Hummingbird-plant interactions

We compiled records on hummingbird-plant interactions from 124 reports (see S1 Table) and four personal communications, analyzing up to 4532 interactions between 292 hummingbird species and 1287 plant species.

The binary network included species records from all the nine hummingbird clades (*sensu* McGuire *et al*. [9, 29]) and from 105 native and non-native plant families (S2 Table). The hummingbird clades and plant families were connected by 409 links. The hummingbird genera with the most species in the matrix were *Amazilia* (27 species), *Phaethornis* (17), *Chlorostilbon* (14), *Coeligena* (9), *Lophornis* (9), *Campylopterus* (8), *Eriocnemis* (7), *Hylocharis* (7), *Anthracothorax* (7), and *Metallura* (7) (Table 2). The plant families with most species used by hummingbirds were Fabaceae (121 species), Bromeliaceae (81), Rubiaceae (70), Lamiaceae (69), Gesneriaceae (54), Ericaceae (51), Acanthaceae (51), Asteraceae (45), Malvaceae (37), and Bignoniaceae (35) (S1 Table).

**Table 2:** Hummingbird clades, genera, and species included in the present study. For each of the nine clades (Clade) included in the network, we show the corresponding genera (Genus) and number of species (Species) per genus.

Clade*	Genus	Species	Clade*	Genus	Species
**Bees**	*Archilochus*	2	**Emeralds**	*Abeillia*	1
	*Atthis*	2		*Amazilia*	27
	*Calliphlox*	4		*Aphantochroa*	1
	*Calothorax*	2		*Campylopterus*	8
	*Calypte*	2		*Chalybura*	2
	*Chaetocercus*	6		*Chlorestes*	1
	*Doricha*	1		*Chlorostilbon*	14
	*Eulidia*	1		*Chrysuronia*	1
	*Mellisuga*	2		*Cyanophaia*	1
	*Myrmia*	1		*Cynanthus*	2
	*Myrtis*	1		*Damophila*	1
	*Rhodopis*	1		*Elvira*	2
	*Selasphorus*	6		*Eupetomena*	1
	*Thaumastura*	1		*Eupherusa*	4
	*Tilmatura*	1		*Goethalsia*	1
**Brilliants**	*Aglaeactis*	4		*Goldmania*	1
	*Boissonnneaua*	2		*Hylocharis*	7
	*Clytolaema*	1		*Klais*	1
	*Coeligena*	9		*Lepidopyga*	2
	*Ensifera*	1		*Leucippus*	4
	*Eriocnemis*	7		*Leucochloris*	1
	*Haplophaedia*	2		*Microchera*	1
	*Heliodoxa*	5		*Orthorhyncus*	1
	*Lafresnaya*	1		*Phaeochroa*	1
	*Loddigesia*	1		*Stephanoxis*	1
	*Ocreatus*	1		*Taphrospilus*	1
	*Pterophanes*	1		*Thalurania*	6
	*Urochroa*	1		*Trochilus*	2
	*Urosticte*	2	**Mountain Gems**	*Eugenes*	1
**Coquettes**	*Adelomyia*	1		*Heliomaster*	4
	*Aglaiocercus*	3		*Hylonympha*	1
	*Chalcostigma*	5		*Lampornis*	6
	*Discosura*	4		*Lamprolaima*	1
	*Heliangelus*	5		*Panterpe*	1
	*Lesbia*	2		*Sternoclyta*	1
	*Lophornis*	9	**Mangoes**	*Androdon*	1
	*Metallura*	7		*Anthracothorax*	7
	*Opisthoprora*	1		*Augastes*	2
	*Oreonympha*	1		*Avocettula*	1
	*Oreotrochilus*	5		*Chrysolampis*	1
	*Oxypogon*	1		*Colibri*	4
	*Phlogophilus*	2		*Doryfera*	2
	*Polyonymus*	1		*Eulampis*	2
	*Ramphomicron*	2		*Heliactin*	1
	*Sappho*	1		*Heliothryx*	2
	*Sephanoides*	2		*Polytmus*	3
	*Taphrolesbia*	1		*Schistes*	1
**Hermits**	*Anopetia*	1			
	*Eutoxeres*	2	**Patagona**	*Patagona*	1
	*Glaucis*	2			
	*Phaethornis*	17	**Topazes**	*Florisuga*	2
	*Ramphodon*	1		*Topaza*	1
	*Threnetes*	3			

* Based on McGuire *et al*. [9, 29].

**Fig 2:**
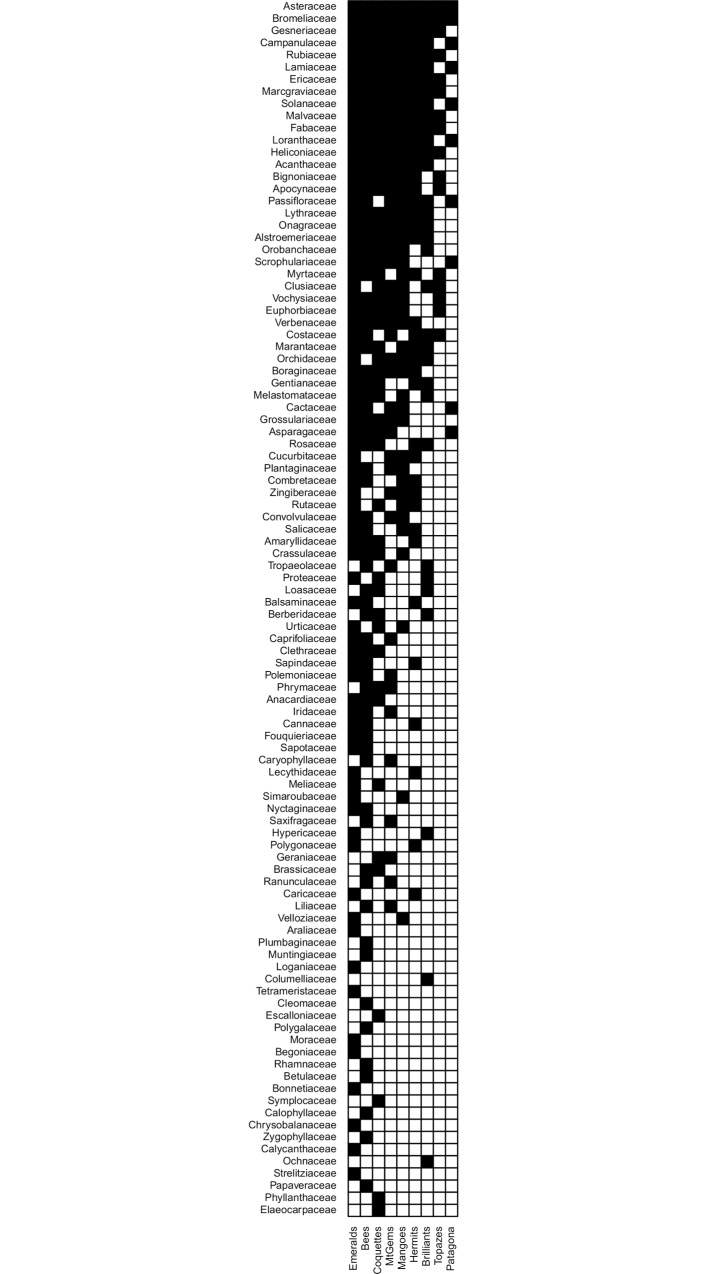
Matrix representation of the interaction network between hummingbirds and their nectar plants. Hummingbird clades are in columns and plant families in rows, and non-native plant species were excluded. Nodes were ordered by number of links exemplifying nestedness pattern, with most pairwise interactions located at the top left corner of the matrix.

The binary matrix with only native plant species also included all the nine hummingbird clades, which interacted with 100 plant families through 385 links (Fig 2, S1 Fig). Plant families such as Myrtaceae, Rutaceae, and Strelitziaceae had fewer connections in this matrix, and families such as Musaceae, Oleaceae, Pittosporaceae, Vitaceae, and Xanthorrhoeaceae were excluded. All species in these families were classified in the network as “exotic.”

**Fig 3:**
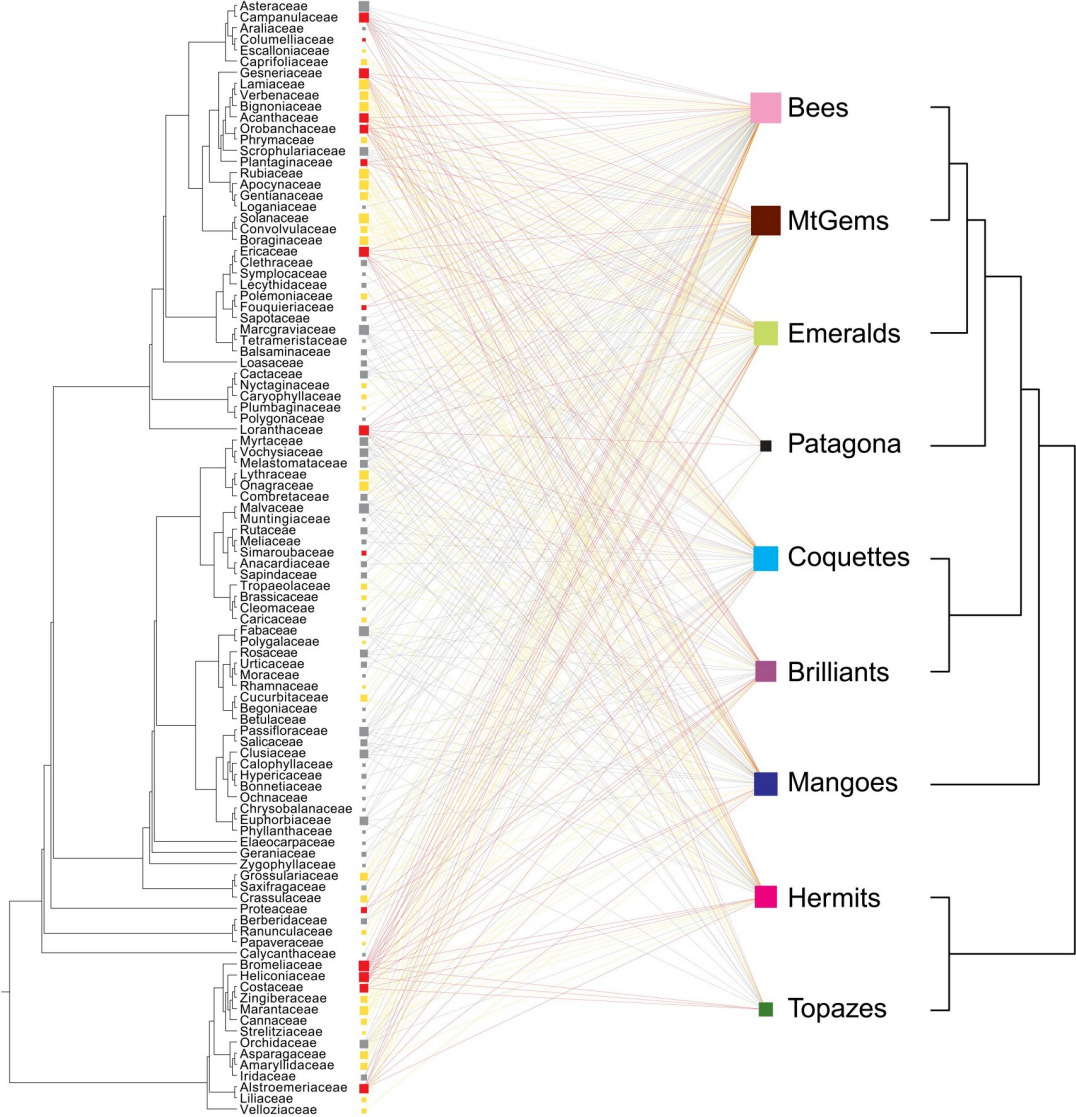
Network of hummingbirds and their nectar plants with nodes ordered phylogenetically. Ecological network of hummingbird clades (right) and plant families (left) (non-native plant species were excluded). Nodes were ordered phylogenetically, and their size was proportional to the number of species with which they interact. The lines represent the recorded 385 interactions. Plants were classified by floral morphology as ornithophilous (red), intermediate (yellow), or non-ornithophilous (gray) (Table 1).

We identified a core composed of generalist nodes including the Bee and Emerald hummingbird clades and the Asteraceae, Bromeliaceae, Gesneriaceae, Campanulaceae, Rubiaceae, Lamiaceae, Ericaceae, Marcgraviaceae, Solanaceae, Malvaceae, Fabaceae, Loranthaceae, and Heliconiaceae plant families (Fig 2, S1 Fig). When the network nodes were ordered phylogenetically (Fig 3), the nodes of the more recent hummingbird lineages (Emeralds and Bees; [9]) were generalist and linked with a greater diversity of plant families from across the plant phylogeny. The nodes of the Hermit, Topaz, and Patagona clades were strongly connected to both basal (Heliconiaceae, Bromeliaceae, Costaceae, Musaceae) and more recent plant families (Asteraceae, Campanulaceae, Gesneriaceae, Lamiaceae, Verbenaceae, Bignoniaceae, Acanthaceae) (Fig 3).

### Network analysis

Regarding connectivity distribution, the network of hummingbird clades and plant families was highly heterogeneous. The network had a few highly connected nodes (generalist), but the bulk of the nodes had few links (specialist). Considering only native plant species, 13 out of the 100 plant families were connected with representatives from all the nine hummingbird clades, and only three hummingbird clades were connected with more than 50 plant families. Additionally, the power-law distribution (AIC = 565.342) fit the observed probability distribution better than the exponential (AIC = 658.581) or truncated power-law (AIC = 656.354) distributions. The same heterogeneity was observed in the matrix including both native and non-native plant species (S2 Fig, S1 Appendix).

When we ordered the nodes based on the connection number, considering only native plant species, the network was highly nested (NODF = 70.680), and hummingbirds and plants showed high NODF values (NODF hummingbirds = 81.050, NODF plants = 70.600) (Fig 2). Compared with the null models, the matrix was statistically different from the ER and CE models (NODF model ER = 43.950, *P* < 0.001, SES model ER = 14.372; NODF model CE = 51.890, *P* < 0.001, SES model CE = 9.443), but not from the FF model (NODF model FF = 69.700, *P* = 0.935, SES model FF = 2.387).

However, when we included phylogeny in the network construction, the nestedness of the network containing only native plants dropped dramatically (NODF = 43.270, NODF hummingbirds = 63.970, NODF plants = 43.120; Fig 3). The network was statistically different from randomly selected networks when using the ER and CC models (NODF model ER = 22.140, *P* < 0.001, SES model ER = 9.763; NODF model CE = 29.510, *P* < 0.001, SES model CE = 6.819) but not the FF model (NODF model FF = 40.829, *P* = 0.835, SES model FF = 5.499). The nestedness value of the matrix including both native and non-native plant species was similar to those described above (S1 Appendix).

**Fig 4:**
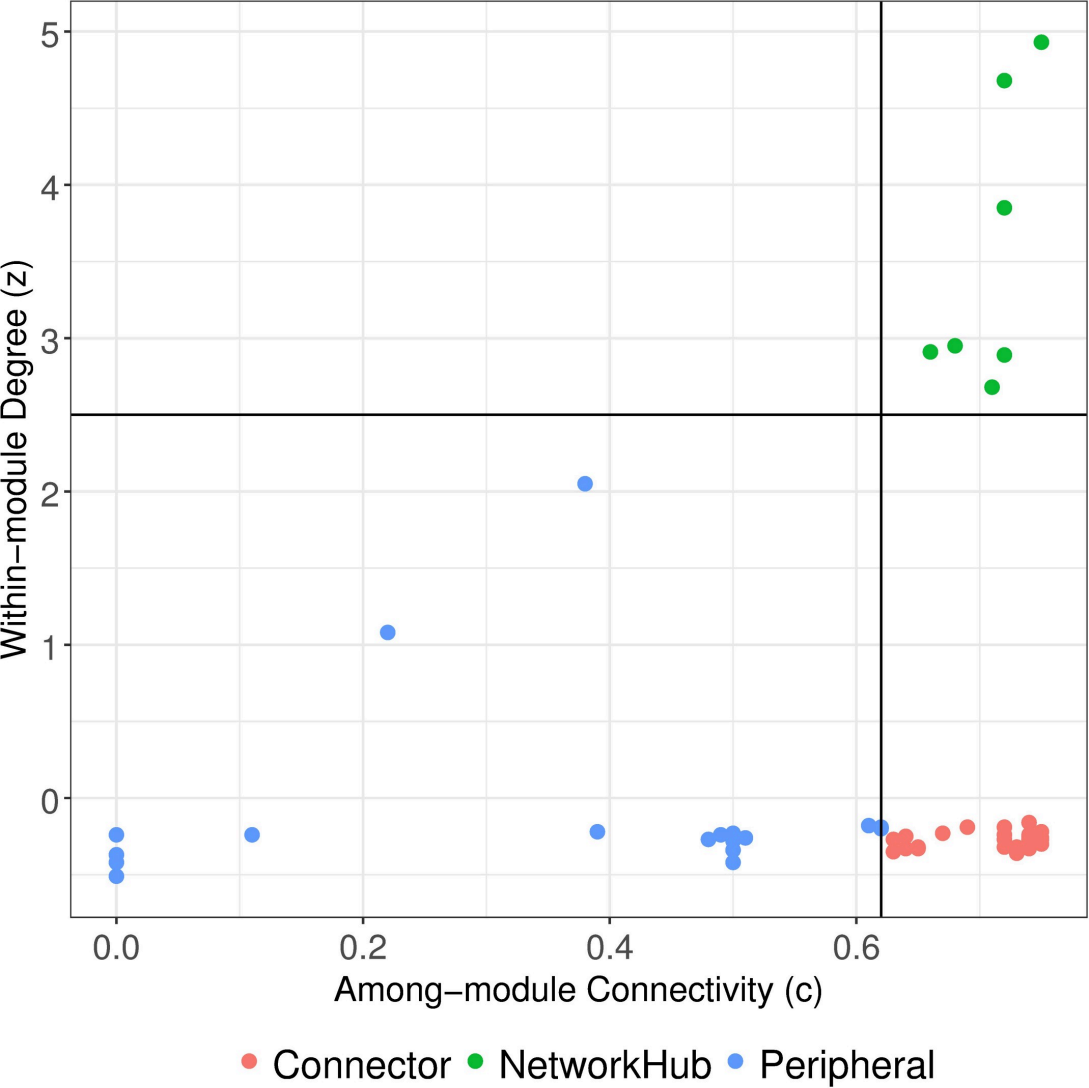
Plot classifying the ecological roles of hummingbirds and their floral nectar resources in a mutualistic network. The standardized within-module degree (*z*) measures how well connected a node is to other nodes in the same module (y-axis). The among-module connectivity (*c*) measures how each node is positioned with respect to all modules (x-axis). The values of 2.5 for *z* (horizontal line) and of 0.62 for *c* (vertical line) are cutoffs to defined node roles following Olesen *et al*.[58]. Peripheral node (z ≤ 2.5, c ≤ 0.62), connector node (z ≤ 2.5 and c ≥ 0.62), module hub (z ≥ 2.5 and c ≤ 0.62), and network hub (z ≥ 2.5 and c ≥ 0.62).

The network was not significantly modular (M = 0.189 ± 0.004; M model ER = 0.204, *P* = 0.97, SES model ER = –3.085; M model FF = 0.214, *P* = 0.957, SES model FF = –5.028). Similarly, the network including both native and non-native plant species was not significantly modular (S1 Appendix). Most links were observed between species from different modules (73.4%), and the mean connectance among modules was high (42.9%). Although the networks were not modular, the analysis with only native plant species identified four (63%) to five (37%) modules. In these modules, the hummingbird clades were associated with several plant families in more than 90% of the repetitions, indicating shared preferences for plants (S3 Table). With the exception of Topazes, all hummingbird clades were frequently associated with plant families whose floral morphology was principally intermediate or non-ornithophilous (S3 Table). No hummingbird clade was associated with another hummingbird clade in more than 90% of the modularity repetitions. Specifically, Bees and Mountain Gems were in the same module in 78% of the repetitions, Brilliants and Coquettes in 70% of the repetitions, Hermits and Mangoes in 56% of the repetitions, and Hermits and Topazes in 42% of the repetitions. The Patagona clade had a variable position in the modules. In contrast, Emeralds were associated with plant nodes but not with any other hummingbird nodes in 76% of the repetitions. Based on the within-module degree *z* (i.e., the standardized number of links to other species in the same module) and the among-module connectivity *c* (i.e., the extent to which species in one module were linked to other modules), the plant and hummingbird nodes played different roles (Fig 4). Plant families acted as *peripheral* or *connector* nodes but were not *module hubs*. All hummingbird clades, except Topazes and Patagona, were “supergeneralist” nodes (*network hubs*), maintaining the cohesion of the network (green dots Fig 4).

### Morphological information

Using floral morphology (Table 1), we found that 50% of the 100 plant families did not have ornithophilous flowers, whereas 36% had intermediate flower morphology and 14% had flowers corresponding with the ornithophilous pollination syndrome (S1 Table). Hummingbirds intensively visited flowers of all morphologies (top right, S1 Fig, Fig 3), but plant families with ornithophilous pollination syndrome were generalist nodes and were intensively visited by representatives of all hummingbird clades (red squares in the top right of S1 Fig). These latter plant families were principally located at the extremes of the plant phylogeny (red squares in Fig 3).

The analysis of hummingbird morphology at the clade level (Table 3) showed that the species in the Emerald and Bee clades, which corresponded with the most recent (Fig 1) and most generalist clades (Fig 2), respectively, had on average short-to-intermediate bills (17–25 mm), short wings (42–54 mm), small body sizes (2.9–4.3 g), and straight bills. In contrast, the species belonging to the oldest clades, Topazes and Hermits, had curved and long and strongly curved beaks, respectively (Table 1).

**Table 3:** Hummingbird morphology at the clade level. For each morphological character, the number of records (n), mean, and standard deviation (mean ± s.d.) are shown. Clades are ordered phylogenetically.

	Exposed culmen (mm)		Wing chord (mm)		Weight (g)		Curvature*	
Clade	n	Mean ± s.d.	n	Mean ± s.d.	n	Mean ± s.d.	n	Mean ± s.d.
Bees	495	17.209 ± 2.192	487	42.620 ± 3.935	1715	2.874 ± 0.569	33	2 ± 0.485
Mountain Gems	301	25.021 ± 5.321	301	67.187± 5.453	315	6.408± 1.187	15	2 ± 0.488
Emeralds	3298	19.490 ± 2.596	2850	54.239 ± 6.069	1820	4.319 ± 1.390	96	2 ± 0.352
Patagona	1	40.7	1	126	3	20.567 ± 2.272	1	2 ± 0
Coquettes	440	14.787 ± 2.677	443	55.981 ± 11.017	206	4.949 ± 1.826	57	2 ± 0.350
Brilliants	426	27.174 ± 6.133	417	70.790 ± 6.502	211	7.009 ± 1.864	42	2 ± 0.407
Mangoes	429	25.809 ± 7.228	418	60.073 ± 6.543	258	5.654 ± 1.663	53	2 ± 0.644
Hermits	942	32.011 ± 6.334	840	55.939 ± 8.986	398	5.240 ± 1.934	183	4 ± 0.772
Topazes	27	11.305 ± 8.096	10	75.440 ± 3.790	19	9.287 ± 2.780	7	3 ± 0

*Curvature: 2, straight; 3, curved; 4, strongly curved.

### Biogeographical distribution and center of diversification

**Fig 5:**
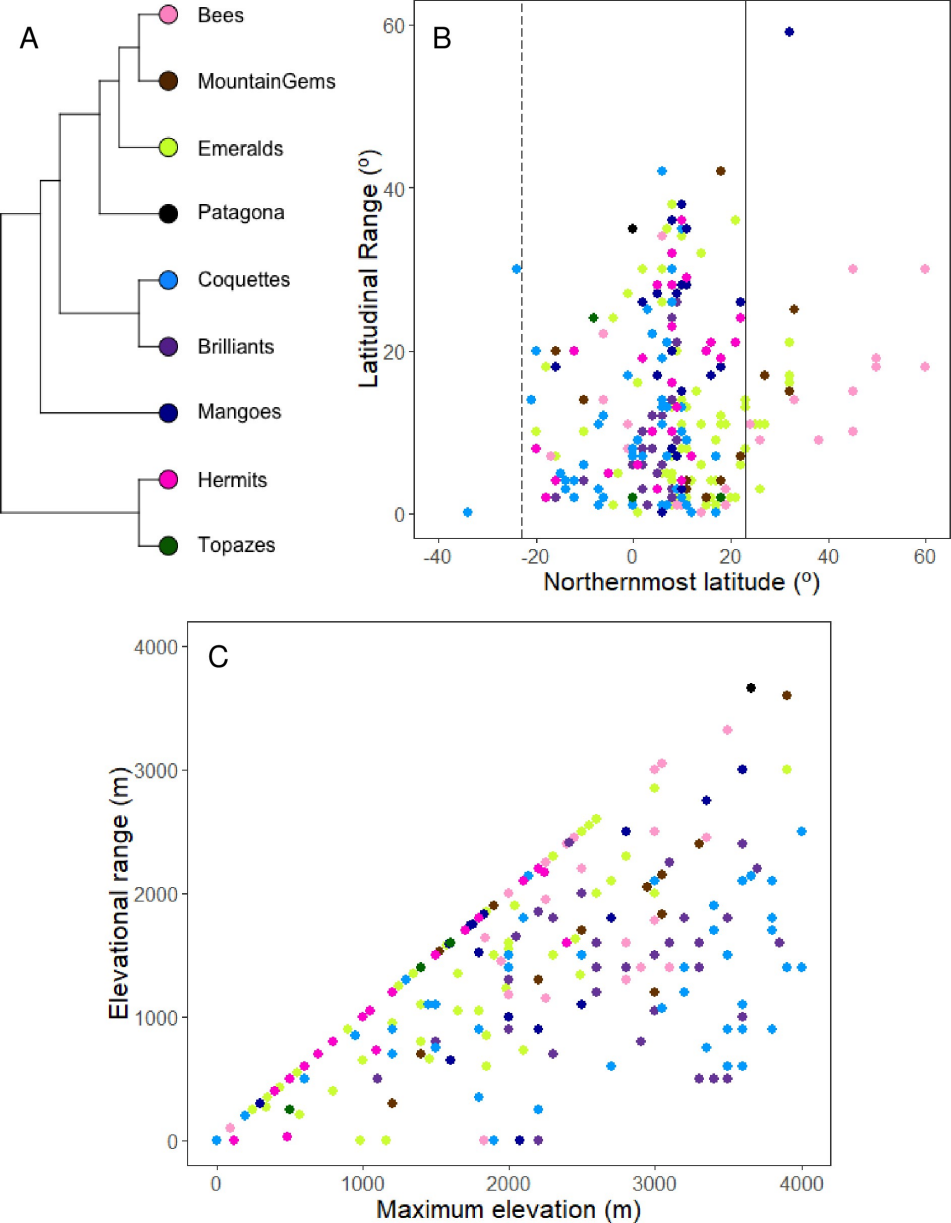
Hummingbird latitudinal and elevational ranges. (A) Phylogenetic tree representing the hummingbird clades. The colors next to the clades match the colors in the plots, identifying the clade to which each species belongs. (B) Latitudinal range of hummingbird species (dots); the x-axis shows the northernmost latitude and the y-axis the mean latitudinal range. The vertical lines cross at the approximate latitude of the Tropic of Cancer (23° N, continuous line) and Tropic of Capricorn (23° S, dashed line) representing an approximate delimitation between the Nearctic, Neotropical and Austral biogeographical regions. For further details about biogeographical regions see text. (C) Elevational range of hummingbird species; the x-axis represents the maximum elevation and the y-axis the elevational range.

Two hundred and seventy (92.5%) of the hummingbird species included in our data set were distributed in the Neotropical region (Fig 5, Table 4, S4 Table), while 13.0% of the hummingbird species (38 species) were distributed exclusively in the Nearctic (3 species) or in the Nearctic and Neotropical regions (35 species). Of these, 20 species are latitudinal migrants, and the remaining species are elevational migrants or sedentary species. Similarly, 9.3% of the hummingbird species in our data set (27 species) were completely (4 species) or partially (23 species) distributed in the Austral region, but only three of these (*Oreotrochilus leucopleurus*, *Patagona gigas*, and *Sephanoides sephanoides*) have latitudinal migrations in South America (S4 Table).

**Table 4:** Biogeographical distribution and center of diversification for hummingbird clades. For each clade, the percentage of species distributed in the two biogeographical areas is shown. Additionally, the diversification center and the mean latitudinal (Lat) and mean elevational (Elev) range of each clade (mean ± standard deviation) were estimated. See text for details on biogeographical areas and center of diversification.

Clade	% Nearctic^a^	% Neotropical^a^	% Austral^a^	Diversification Center^b^	Mean Max Lat (°)^c^	Mean Min Lat (°)^c^	Mean Lat Range (°)	Mean Elev Min (m)^d^	Mean Elev Max (m) ^d^	Mean Elev Range (m)
Bees	36.364	81.818	9.091	North America	19.300 ± 21.131	7.567 ± 19.772	11.733	551.833 ± 604.715	2327.333 ± 792.586	1775.500
Mountain Gems	40.000	100.000	0.000	North America	13.667 ± 13.308	2.667 ± 16.387	11.000	621.333 ± 548.216	2251.667 ± 922.848	1630.333
Emeralds	16.667	91.667	6.250	South America	9.878 ± 11.355	−0.011 ± 14.835	9.889	296.889 ± 408.318	1657.500 ± 874.586	1360.611
Patagona	0.000	100.000	100.000	South America	0	−35.000	35.000	0	3660	3660.000
Coquettes	1.887	94.340	24.528	South America	0.453 ± 11.360	−11.548 ± 13.929	12.000	1608.208 ± 1205.671	2807.453 ± 1344.139	1199.245
Brilliants	0.000	100.000	7.895	South America	3.579 ± 6.954	−6.895 ± 8.953	10.474	1640.789 ± 907.763	2973.947 ± 819.316	1333.158
Mangoes	7.407	81.481	3.704	South America	7.182 ± 11.839	−13.591 ± 12.308	20.773	427.500 ± 599.042	1674.318 ± 958.464	1246.818
Hermits	3.846	100.000	0.000	South America	4.538 ± 11.704	−11.923 ± 11.527	16.462	69.846 ± 186.879	1130.346 ± 648.366	1060.500
Topazes	0.000	100.000	0.000	South America	3.333 ± 13.317	18.000 ± 15.010	21.333	83.333 ± 144.338	1166.667 ± 585.946	1083.333

^a^ ebird [73], del Hoyo *et al*. [74]

^b^ McGuire *et al*.[9], [18]

^c^ Ornelas [76]

^d^ Arizmendi *et al*. [79], Bleiweiss [80], del Hoyo *et al*. [74]

In general, the hummingbird clades had large latitudinal ranges, but clades with North America as their center of diversification showed smaller mean latitudinal ranges than their southern counterparts (Table 4). All of the clades are mainly distributed in the Neotropical biogeographical region, but a significant proportion of the species belonging to the three most recent hummingbird clades are distributed in the Nearctic region (Emeralds, Bees, and Mountain Gems; Fig 5A, Table 4). Some species in these latter three clades reach the northernmost latitude (60° N), while their minimum latitudinal range is around 0° N. Specifically, *Selasphorus rufus* and *Archilochus colubris* (Bee clade) reach 60° N during the breeding season (Fig 5A, S4 Table). In South America, only two species (*Sappho sparganura* and *Sephanoides sephanoides*) are located below the minimum latitudinal range of −39° S (−40° and −54° S, respectively; S4 Table); of these latter two species, only *S*. *sephanoides* migrates latitudinally. In contrast, the Brilliant clade has a restricted distribution evidenced by its low mean latitudinal range and standard deviation (Table 4). The oldest clades (Mangoes, Hermits, and Topazes) mainly have a Neotropical distribution and are widely distributed in South America (Fig 5A, Table 4).

Regarding elevational range, Patagona has the largest range, followed by the Bee and Mountain Gem clades, while the Brilliant, Mango, Coquette, and Emerald clades have intermediate ranges (around 1300 meters above sea level) (Table 4). Hermit and Topazes were restricted to lower elevations, in contrast with Brilliants and Coquettes, which were principally related with Andean high elevations (above 1600 meters above sea level; Fig 5B, Table 4).

**Fig 6:**
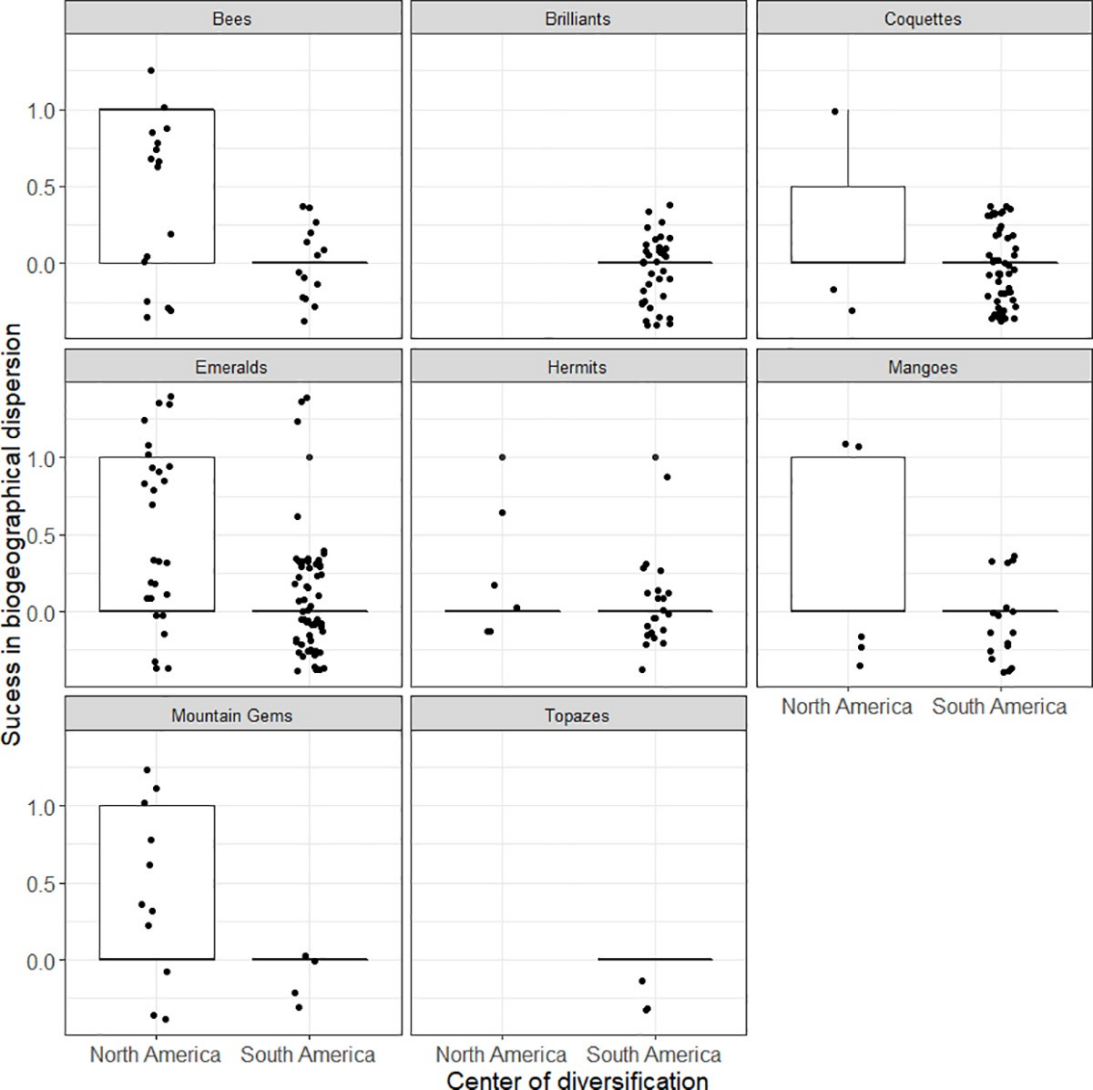
Hummingbird niche evolution and colonization. Relationship between the center of diversification and current distribution of hummingbirds in one (0 value on y axis) or in two or three (1 value on y axis) biogeographical regions. Each dot in the plots represents a hummingbird species, and these are faceting by clade. The Patagona clade was excluded because it contains only one South American species, *Patagona gigas*. The box encloses 50% of the data and is divided by the median (horizontal line); the upper and lower adjacent lines indicate the 0.95 and 0.05 quartiles, respectively.

The analysis of niche conservatism in hummingbirds revealed that the center of diversification had a significant effect on the transition of hummingbird species from one biogeographical region to another (Chi = 46.114, d.f. = 1, *P* < 0.001). The probability that a hummingbird species would colonize a different biogeographical region was higher for hummingbirds with a center of diversification in North America compared to those with a center of diversification in South America (GLM odds ratio = 9.080, Chi = 43.679, d.f. = 1, *P* < 0.001, Fig 6). Additionally, clade had a significant effect on current hummingbird distribution (Chi = 21.887, d.f. = 7, *P* = 0.003). In general, clades that diversified in North America had a higher proportion of species currently distributed in two biogeographical regions. However, a small proportion of species belonging to clades that diversified in South America (Coquettes, Hermits, and Mangoes) had a North American origin, although few of these species successfully dispersed in the Neotropical region (Fig 6).

Additionally, the two clades with species that exclusively diversified in South America (Brilliants and Topazes) did not colonize the Nearctic biogeographical region (Fig 6). Species in the Bee clade differed significantly from those in the Brilliant, Hermit, and Mango clades and also had a higher probability of colonizing new biogeographical regions (GLM odds ratio = 3.186, Chi = 9.462, d.f. = 1, *P* = 0.002; GLM odds ratio = 4.552, Chi = 8.910, d.f. = 1, *P* = 0.003; GLM odds ratio = 2.324, Chi = 4.297, d.f. = 1, *P* = 0.038, respectively; Fig 6). The same pattern was found for species of the Emerald and Mountain Gem clades, which had a higher probability of colonizing two biogeographical regions compared to those of the Brilliant clade (GLM odds ratio = 1.927, Chi = 3.904, d.f. = 1, *P* = 0.048; GLM odds ratio = 3.186, Chi = 6.870, d.f. = 1, *P* = 0.009, respectively; Fig 6). Finally, species in the Hermit clade had a lower probability of colonizing new biogeographical regions compared to those in the Emerald clade (GLM odds ratio = 2.752, Chi = 4.537, d.f. = 1, *P* = 0.033, Fig 6).

## Discussion

The network between hummingbird clades and plant families at the continental scale was nested and heterogeneous, as expected, and behaved similarly to other mutualistic networks (e.g., [13, 89]). By incorporating phylogenetic, morphological, ecological and biogeographical information, we found that low niche conservatism, low specialized morphologies and recent diversification were the determinant factors that differentiate a generalist hummingbird clade node from a specialist hummingbird clade node. We detected large-scale biogeographical patterns indicative of both niche conservatism and evolution that support the higher biodiversity found near the equator [4].

### Hummingbird-plant interaction network

Mutualistic networks have been shown to display universal patterns in architectural properties such as connectivity, nestedness and modularity. These facts imply that the mechanisms behind the establishment of plant-animal interactions are likely independent of species composition, place, and time [23–25].

Some authors suggest that selecting a suite of null models allows inferring ecological mechanisms and their role in nestedness pattern because each null model excludes and/or constricts a specific factor of the original network [23, 55, 56]. We found that our hummingbird-plant network was significantly heterogeneous and nested depending on the null model. The nested pattern between hummingbird clades and plant families was explained by the overall connectivity and generalization pattern of hummingbirds and plants (significant CE null model) but not by the number of links per node nor plant and hummingbird phylogeny (no significant FF null model). Regarding the generalization pattern, this network has a core formed by generalist nodes composed of two hummingbird clades and 13 plant families (Fig 2, S1 Fig). Some of these nodes are remarkable for their high diversity and wide distribution. The Emerald clade is a large, taxonomically complex, and widely distributed hummingbird assemblage whose diversification in South America was favored by the Andean uplift [18, 29]. With respect to plants, the Asteraceae, Fabaceae, and Rubiaceae families are recognized for their high diversity and widespread distribution (25040, 19580, and 13150 species, respectively) [90]. Because the core concentrates a high number of interactions and contributes notably to the nestedness of the network, it possibly controls the selective forces acting on the specialist nodes and plays an important role in co-evolutionary processes and network persistence [23, 91]. Additionally, competition has been described as one of the principal mechanisms influencing the interaction between hummingbirds and plants [92–96]. Nestedness reduces interspecific competition and enhances the number of co-existing species [97]. The architecture of our network indicates that species co-existence and network persistence and stability are positively favored [91, 97, 98], allowing hummingbirds to be linked with an incredibly high number of plant species.

In contrast with our predictions about network structure, the hummingbird-flower network studied herein was not significantly modular (see Results), even though different studies on hummingbird-plant networks have detected significant modularity [20, 89, 99]. Previously, an inverse relationship between modularity and nestedness was described. A highly modular network is more specialized and less tolerant to lost connections [24]. However, because our hummingbird-plant network was constructed at a coarse scale (less than 50 nodes for hummingbirds) and the majority of connections were between modules (not intra modules), modularity was not detected [58]. One relevant finding was that, even at this scale, hummingbird nodes acted as module connectors, confirming that many hummingbirds have evolved the ability to interact with multiple plant partners [27, 100]. The hummingbird clades were associated at the same module with several plant families in more than 90% of the repetitions, indicating shared preferences for plants. It is possible that a more detailed analysis (at the species or genera level) would detect modules between hummingbirds and plants, offering a finer level of resolution to this mutualism.

By excluding exotic plant species from the analyses, we assumed that the original interactions in the network would remain intact. This assumption was not validated in previous studies on several plant communities where exotic species significantly changed the connections between the native nodes [101–105]. When we compared the architecture of the networks including or excluding exotic plant species, the network including exotic plant species connected the same nine hummingbird clades with the same 100 plant families, although we also recorded more connections with several plant families such as Myrtaceae, Rutaceae, and Strelitziaceae. However, the effects of non-native plants on network measurements such as nestedness and modularity were not statistically significant (see Results), probably because of the large scale used in our study (the hummingbird clade and plant family level).

Current ecological processes define the structure of ecological networks, although other factors such as the phylogenetic history and co-evolutionary dynamics of species interacting in a network are also important and measurable by network metrics such as nestedness and modularity [14, 27, 30, 58, 98]. When phylogeny was taken into account (i.e., the phylogenetic position of nodes was introduced into the network construction), network nestedness dropped dramatically. However, an interesting phylogenetic pattern emerged in the network: The hummingbird generalist nodes (Bees and Emeralds) are recent hummingbird lineages [9] that connect an important diversity of distantly-related plant families. Additionally, the oldest nodes, Hermits and Topazes, were strongly connected to both basal and more recent plant families (Fig 3). The decrease in the nestedness value agrees with a scenario in which subsets of nodes are highly connected internally, increasing network modularity [58]. In this respect, Martín Gonzalez *et al*. [13] studied 54 hummingbird-plant community networks across the Americas and found an association between network structure and phylogenetical signal node. Notably, complementary specialization and modularity also increased when closely related hummingbird species visited different sets of plant species, suggesting a close co-evolutionary relationship between hummingbirds and their plants. The results Martín Gonzalez *et al*. [13] in addition to the change in the connection pattern found in our network when phylogeny was considered suggests that both ecological and evolutionary processes are driving the mutualistic interactions between hummingbirds and their nectar resources. In particular, for Bee and Emerald hummingbirds, important peaks in speciation rates occurred during the last 5 MYA [9, 18, 81], and the Bee clade is the most recently derived and most rapidly diversifying group of hummingbirds [9]. Thus, in our network, the most recent hummingbird clades were generalist and connected with both old and recent plant families, exemplifying how recent hummingbirds evolved at a time when plant diversity was higher and likely used this diversity as an ecological advantage [106].

Additionally, from an evolutionary perspective, the generalist plant families in our network belonged to both recent and old plant lineages (Fig 3), suggesting that the interaction of hummingbirds and plants, over evolutionary time, promoted distinct patterns of plant diversification. For example, the old family Heliconiaceae is principally distributed in tropical America, and the mutualistic relationship between species in this family and hummingbirds has been well documented [107, 108]. Meanwhile, in the old family Bromeliaceae, only more recent lineages have adapted to hummingbird visitation [19, 109]. For recent cosmopolitan and generalist plant families such as Gesneriaceae, Campanulaceae, Lamiaceae, and Acanthaceae, different studies have suggested that hummingbird pollination triggered plant diversification, which is supported by the species diversity of Paleo vs. Neotropics lineages [19, 110–112]. Our findings additionally support Jordano’s affirmation that the evolution of super-generalist nodes allows for the connection of diverse blocks that build the architecture of ecological services and biodiversity [27].

### Morphological information

Besides the aforementioned phylogenetic history, the architecture of complex networks also relies on inherited traits [14, 22]. In the particular case of hummingbird-plant networks, hummingbird traits (bill length, tongue extension, bill curvature, and body mass) and flower traits (length and curvature of corolla and nectar production) determine interaction frequencies and network structure [99, 113, 114]. We found a generalization gradient in hummingbird morphology in the studied interaction network. Hummingbirds of recent and generalist clades (Bees, Mountain Gems, and Emeralds; Fig 3) are less morphologically specialized than those of other clades, i.e., hummingbirds of generalist clades were medium-sized and had predominantly straight beaks of medium length. The opposite pattern was observed in southern and specialist clades such as Hermits, Mangoes, and Patagona, which contained larger hummingbirds with longer and more curved beaks (Table 3). With respect to floral morphology, hummingbirds intensively use plant families with floral morphologies exhibiting the ornithophilous syndrome but also visit flowers with morphologies that are not typically adapted for hummingbird pollination [100, 115] (Fig 3, Table 1). In some communities, the frequency of use of non-ornithophilous plant species reflects periods of scarcity of ornithophilous species [20]. However, some of the generalist plant nodes of this network have intermediate and non-ornithophilous flowers (such as Asteraceae, Rubiaceae, and Lamiaceae, Fig 3), and the inclusion of these nodes is consistent with their pattern and frequency of use.

It has been suggested that high phenotypic specialization in hummingbirds as well as morphological matches between plant and bird species explains the network architecture between hummingbird and flower communities at a global scale [89]. Our results partially support these suggestions. Plant species belonging to genera such as *Heliconia*, *Salvia*, *Palicourea*, and *Tillandsia* are reportedly pollinated by specialized hummingbirds [19, 110] and, as expected, many hummingbird species in our network have connections with them [116–120] (S1 Table), especially hummingbirds belonging to the oldest clades (Mangoes, Topazes, and Hermits) with curved or strongly curved beaks, as this character is related with high specificity in nectar resources [114] (Fig 1, Table 1). However, the pattern of hummingbird-plant interactions and the inclusion of plant families with a large diversity of shapes and sizes in the network (not exclusively limited to those with tubular flowers of the ornithophilous syndrome) was a consequence of the intermediate morphology of the more generalist hummingbird clades (Table 3). This generalist behavior agrees with the findings of Vitória *et al*. [14] in the Atlantic Forest of Brazil where hummingbirds interacted with plants irrespective of plants’ evolutionary history. In the case of the Bee clade, the observed morphology of these hummingbirds combined with the low variation in their body mass and wing morphology [121] was found to favor access to the nectar of flowers with and/or without hummingbird pollination adaptations, reducing the physiological restrictions imposed by size [18]. Finally, the interaction between plant species and hummingbirds with a broad range of morphologies can have important ecological implications, such as, for example, favoring the diversification of flower size, which was confirmed by Serrano-Serrano *et al*. [122] for the *Nematanthu*s clade (Gesneriaceae).

### Biogeographical distribution and center of diversification

The network between hummingbird clades and plant families offers relevant clues about the influential factors behind the continental hummingbird biodiversity pattern. Our results suggest that the connection pattern of this network was determined by phylogenetic history and morphology (as discussed before) but was also influenced by the biogeographical distribution and center of diversification of hummingbirds. The Tropical Niche Conservatism (TNC) hypothesis is one of the models used to explain large-scale biogeographical patterns and, in particular, the higher levels of biodiversity found near the equator [15]. The hummingbird diversity pattern that we found adjusted to this model, as higher hummingbird biodiversity is found in the Neotropics [7, 17]. An important assumption of the TNC model is that most extant clades originated in the tropics during the mid-Tertiary when the tropics had a greater extension compared to temperate regions. Consequently, the presence of hummingbirds for a long period of time in the tropics in conjunction with the comparatively greater area of the tropics favored a high rate of speciation in the tropics and the specialization of these species to tropical environments [15]. The niche concept is consistent with the TNC hypothesis, and tropical lineages usually present patterns of niche conservatism [16]. The stem group diversification of hummingbirds probably started in the Paleogene (around 65–22 MYA) [9, 81]. Then, the subsequent colonization of the Nearctic and/or Austral biogeographical regions by hummingbird species from northern and central South America is consistent with the prediction of niche evolution because the transition from Neotropical to Nearctic or Austral regions represents an environmental turnover from relatively constant temperatures to a seasonal environment [12, 15, 16].

The biogeographical patterns revealed for hummingbirds were also consistent at the clade level and with respect to latitudinal and elevational range. In this regard, niche conservatism is also relevant for understanding the observed connection patterns. In particular, the Bee and Mountain Gem clades, which diversified in North America in highly seasonal environments [9, 18], had a high probability of colonizing a different biogeographical area and significantly contributed to the high species diversity of North and South America (Fig 6, Table 4). Some authors have suggested that hummingbird evolution under drastic climatic conditions represents an evolutionary advantage that allowed hummingbirds to colonize and expand their distribution to places with less harsh environments such as the tropics [12, 16]. Interestingly, these latter two clades (Bees and Mountain Gems) evolved recently (Fig 3) and, as mentioned earlier, were connected with an important number of plant families favored by intermediate morphologies and low niche conservatism. As mentioned earlier, McGuire *et al*. [9] detected clade-specific processes in hummingbirds and, particularly for the Bee, Emerald, and Mountain Gem clades, reported accelerated speciation rates. These researchers concluded that the rapid rates of diversification in hummingbirds were consistent with classical examples of rapid adaptive radiation. Our data offer additional support for their conclusions: The generalist behavior of these clades, which successfully use all available nectar resources, along with their ability to colonize “new” areas such as Central America and the Andes Mountains, could have favored the recent and accelerated speciation of these clades. The recent colonization of these clades was supported also by Abrahamczyk & Renner [19], who reported that several Emerald and Mountain Gem species have extended their ranges in a northern direction during the last 100 years.

The hummingbird clades that diversified in tropical South America exhibited a lower probability of colonizing new biogeographical areas, generally had larger latitudinal ranges but smaller elevational ranges, and contributed very little to species diversity in temperate North America, with the exception of the Emerald clade (Figs 5 and 6, Table 4). The case of the South American Brilliant and Coquette clades is interesting because hummingbirds from these clades had a generalist morphology but had a small elevational and latitudinal distribution and were mainly restricted to high elevations in the Andes Mountains [7]. Our data suggested that, particularly for these species, specialization occurred at the habitat level (not morphologically), as these birds are highly adapted to high mountain ecosystems, which is consistent with the tropical niche conservatism hypothesis.

Licona-Vera & Ornelas [18] proposed that the colonization of North America and the radiation of Bee hummingbirds in this region were favored by the repeated evolution of long-distance seasonal migration in different lineages and by the availability of favorable habitats and climatic conditions related with the formation of mountain systems in Mexico and Central America. Most long-distance seasonal migrant hummingbirds belong to the Bee clade, and 11 hummingbird species with variable latitudinal ranges (from 660 km to 6000 km) migrate across the Nearctic and the Neotropical regions. Bee species such as *Archilochus colubris* make flights of around 3300 km from their wintering areas to reproduction areas, and *Selasphorus rufus* and *S*. *calliope* travel even greater distances of 6000 and 4500 km, respectively [74, 79]. Although migrant populations have been reported for six Emerald species, their latitudinal ranges are smaller compared to those of the five Bee species that migrate in the same area (S4 Table). Similarly, some species belonging to the Patagonia and Coquette clades migrate latitudinally in South America [18]. For these species, migrations of 2000 km for *Patagona gigas* and 1300 km for *Sephanoides sephanoides* were reported between wintering and summering lands in South America. Notably, for *Oreotrochilus leucopleurus*, records suggest that this species migrates elevationally, but recent records in Bolivia during the Austral winter suggest that it latitudinally migrates [74]. Abrahamczyk & Renner [19] stated that the interaction between migratory vertebrates and nectar resources is likely not the result of co-evolution, which was supported by our results: Generalist clades with long-distance migrant species and populations (such as the Bee and Emerald hummingbirds) visited a diverse array of plants during migration [94, 123, 124].

Our study provided new insight into the factors influencing the interaction between hummingbirds and their nectar resources at a continental scale. Besides their recognized and relevant role as pollinators, from a network perspective, hummingbirds play an important role in the structure and maintenance of ecosystem services and biodiversity, acting as generalist and super-generalist nodes that connect with an amazingly high number of plant species [27, 100]. Given the current rates of habitat modification and the predicted changes in the distributional range of many species as a result of climate change, the loss of species as well as the mutualistic interactions that maintain different processes in natural ecosystems seems inevitable [36]. Understanding the pattern of contemporary specialization between hummingbirds and plants used by hummingbirds as nectar resources at a continental scale can provide insight into how hummingbird lineages with different biogeographical and evolutionary histories might respond to the biodiversity crisis and how the connection pattern between hummingbirds and their nectar resources might be affected by habitat modification and climate change.

It is relevant to further explore the approximations proposed herein at a smaller scale (genus, species), as the necessary information is currently available. Future studies can use quantitative matrices and dated phylogenies for hummingbirds and plants to explore plant niche evolution and to measure phylogenetic signaling in hummingbirds and plants with respect to morphological traits in order to disentangle the evolutionary, colonization, and connection patterns of hummingbird-plant mutualisms in greater detail.

## Supporting information

S1 Fig Network of hummingbirds and their floral nectar resources with nodes ordered by number of links. Ecological network of hummingbird clades (left) and plant families (right) (non-native plant species were excluded). Nodes were ordered by intensity, with the generalist nodes at the top and the specialist nodes at the bottom. Node size is proportional to the number of species with which each species interacts. Plants and interactions (lines) were classified by floral morphology as ornithophilous (red), intermediate (yellow), or non-ornithophilous (gray).
(TIF)

S2 Fig Degree distribution for the number of interactions. This graph shows the cumulative frequency distribution (P (*k*)) of the number of links (*k*) in the hummingbird-plant network (without non-native plant species). The graph in the log-log plot combines plant and hummingbird interactions. The original data (circles) were adjusted to three distributions: (1) power-law function (pow.), (2) exponential (exp.), and (3) truncated power-law (pow.trun.). The network has the best fit with the power-law function (AIC exp. = 658.581, AIC pow. = 565.342, AIC pow.trun. = 656.354).
(TIFF)

S1 Table Plant families and genera visited by hummingbirds. Plant species were grouped by family (FAMILY) and genus (GENUS). For each family, the number of recorded interactions (total number of 1s in the qualitative matrix for each family) (INTER.FAM), visiting hummingbird species (HUMM.FAM), and visiting hummingbird clades (CLAD.FAM) is indicated. For each genus, the number of species used by hummingbirds (SPECIES) and recorded interactions (total number of 1s in the qualitative matrix for each genus) (INTER.GEN) is indicated. Depending on the morphology of the plant species, we classified the pollination syndrome of each family (SYND) as ornithophilous (O), intermediate (I), or non-ornithophilous (NT). See Table 1 in the text for details on the characteristics of each category.
(DOCX)

S2 Table Binary matrix of the interaction network between hummingbird clades and their nectar family plants. Hummingbird clades are in columns and plant families in rows; native and non-native plant species were included. In this binary matrix, 1 indicates an interaction between a hummingbird clade and a plant family, and 0 otherwise. Nodes are ordered by number of links.
(DOCX)

S3 Table Plant families and hummingbird clades associated by modularity analysis. Plant families associated with hummingbird clades in more than 90% of the modularity analyses. We used the network with nodes ordered phylogenetically and only included native plant species. Numbers in parenthesis correspond to the number of analyses in which the plant family belonged to the same module as the hummingbird clade. The text color represents the pollination syndrome of each family: red (ornithophilous), blue (intermediate), and black (non-ornithophilous). See Table 2 in the text for details on the characteristics of each pollination syndrome category.
(DOCX)

S4 Table Biogeographical, elevational, latitudinal, and morphological information and center of diversification for the hummingbird species included in this study. The source(s) of information for each category is (are) shown in the REF column and the Supplementary References section (see below). For the biogeographical distribution regions, the value 1 represents the presence of a hummingbird species in this (these) region(s); in contrast, 0 represents the lack of records. The latitudinal and elevational range columns are the difference between the minimum and maximum value for each category. The asterisk (*) in the Geographical Area column means that the center of diversification of these hummingbird species was indirectly inferred based on the closest sister species whose center of diversification has been explicitly detected. Also, the inference method used in the different studies to establish the center of diversification area is shown. For the definition of each biogeographical region and geographical area, see the text. For exposed culmen, weight, and wing morphological information, the mean and standard deviation (s.d.) of each hummingbird species are shown. The definition of each bill curvature category is explained in the text.
(DOCX)

S1 Appendix Network analysis of the matrix including native and exotic plant species. Results for the degree distribution, nestedness, and modularity analyses of the mutualistic network of hummingbird clades and their nectar plants. In this network, we included all records independently of plant origin (native or exotic to the American continent).
(DOCX)
