# Supplementary figures and images for: Are hummingbirds generalists or specialists? Using network analysis to explore the mechanisms influencing their interaction with nectar resources
Source: PLoS One. 2019 Feb 27;14(2):e0211855. doi: 10.1371/journal.pone.0211855 (PMC6392410; doi:10.1371/journal.pone.0211855)

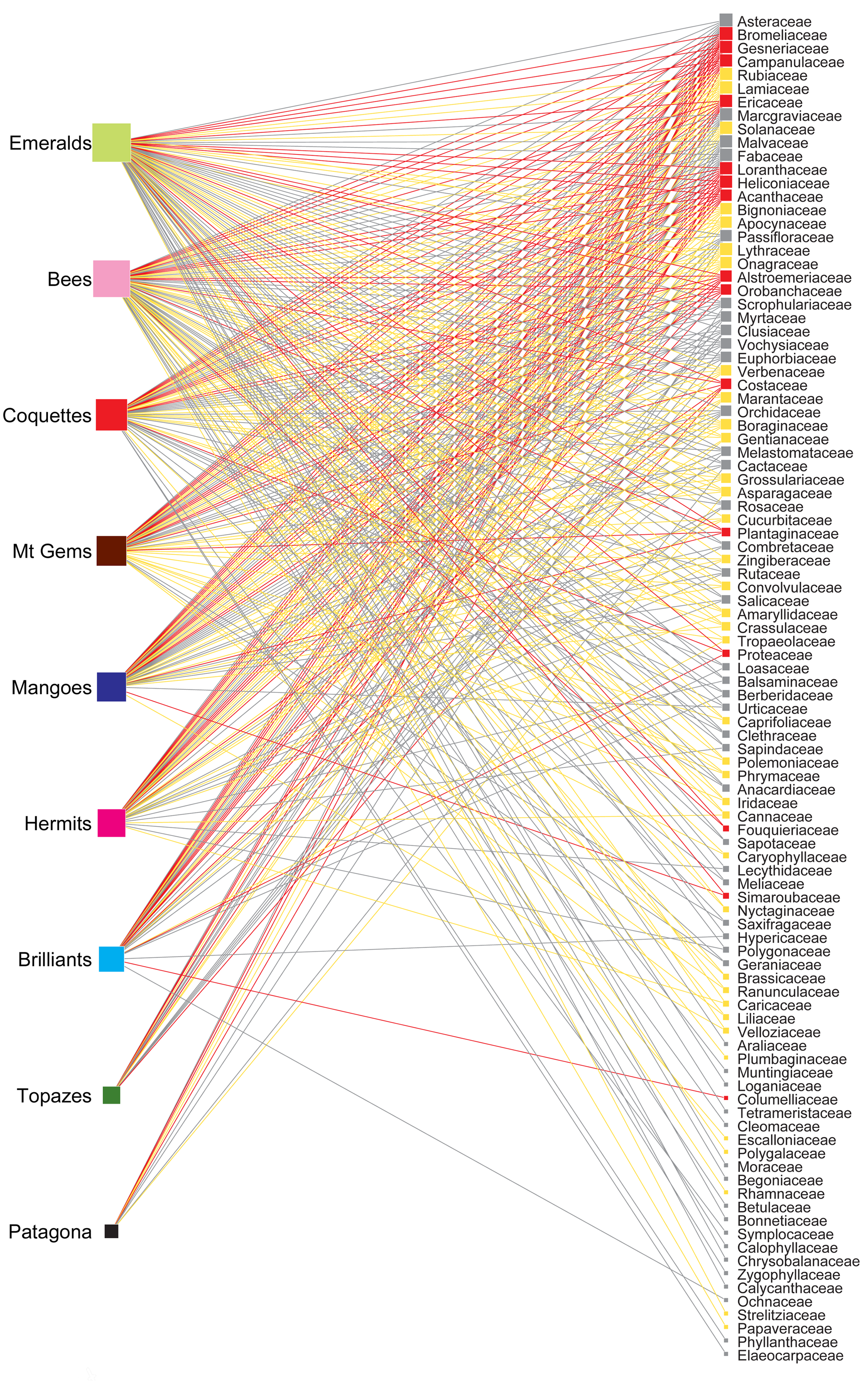

Supplement: S1 Fig — Ecological network of hummingbird clades (left) and plant families (right) (non-native plant species were excluded). Nodes were ordered by intensity, with the generalist nodes at the top and the specialist nodes at the bottom. Node size is proportional to the number of species with which each species interacts. Plants and interactions (lines) were classified by floral morphology as ornithophilous (red), intermediate (yellow), or non-ornithophilous (gray). (TIF) [file pone.0211855.s001.tif]

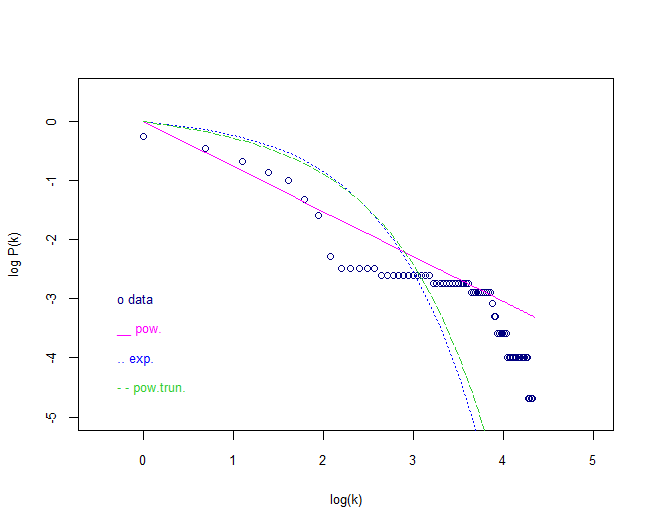

Supplement: S2 Fig — This graph shows the cumulative frequency distribution (P (k)) of the number of links (k) in the hummingbird-plant network (without non-native plant species). The graph in the log-log plot combines plant and hummingbird interactions. The original data (circles) were adjusted to three distributions: (1) power-law function (pow.), (2) exponential (exp.), and (3) truncated power-law (pow.trun.). The network has the best fit with the power-law function (AIC exp. = 658.581, AIC pow. = 565.342, AIC pow.trun. = 656.354). (TIFF) [file pone.0211855.s002.tiff]
