# Supplementary material for: Are hummingbirds generalists or specialists? Using network analysis to explore the mechanisms influencing their interaction with nectar resources
Source: PLoS One. 2019 Feb 27;14(2):e0211855. doi: 10.1371/journal.pone.0211855 (PMC6392410; doi:10.1371/journal.pone.0211855)
Supplement: S1 Table — Plant species were grouped by family (FAMILY) and genus (GENUS). For each family, the number of recorded interactions (total number of 1s in the qualitative matrix for each family) (INTER.FAM), visiting hummingbird species (HUMM.FAM), and visiting hummingbird clades (CLAD.FAM) is indicated. For each genus, the number of species used by hummingbirds (SPECIES) and recorded interactions (total number of 1s in the qualitative matrix for each genus) (INTER.GEN) is indicated. Depending on the morphology of the plant species, we classified the pollination syndrome of each family (SYND) as ornithophilous (O), intermediate (I), or non-ornithophilous (NT). See Table 1 in the text for details on the characteristics of each category. (DOCX) [file pone.0211855.s003.docx]

**S1 Table. Plant families and genera visited by hummingbirds.** Plant species were grouped by family (FAMILY) and genus (GENUS). For each family, the number of recorded interactions (total number of 1s in the qualitative matrix for each family) (INTER.FAM), visiting hummingbird species (HUMM.FAM), and visiting hummingbird clades (CLAD.FAM) is indicated. For each genus, the number of species used by hummingbirds (SPECIES) and recorded interactions (total number of 1s in the qualitative matrix for each genus) (INTER.GEN) is indicated. Depending on the morphology of the plant species, we classified the pollination syndrome of each family (SYND) as ornithophilous (O), intermediate (I), or non-ornithophilous (NT). See Table 1 in the text for details on the characteristics of each category.

**S1 Table**

| **FAMILY** | **INTER.FAM** | **HUMM.FAM** | **CLAD.FAM** | **SYND** | **GENUS** | **SPECIES** | **INTER.GEN** |
| --- | --- | --- | --- | --- | --- | --- | --- |
| Acanthaceae | 152 | 81 | 7 | O | *Anisacanthus* | 6 | 16 |
|  |  |  |  |  | *Aphelandra* | 4 | 14 |
|  |  |  |  |  | *Asystasia* | 1 | 3 |
|  |  |  |  |  | *Avicennia* | 1 | 1 |
|  |  |  |  |  | *Barleria* | 1 | 3 |
|  |  |  |  |  | *Beloperone* | 1 | 1 |
|  |  |  |  |  | *Bravaisia* | 1 | 1 |
|  |  |  |  |  | *Dicliptera* | 2 | 2 |
|  |  |  |  |  | *Graptophyllum* | 1 | 1 |
|  |  |  |  |  | *Habracanthus* | 1 | 3 |
|  |  |  |  |  | *Hansteinia* | 1 | 1 |
|  |  |  |  |  | *Jacobinia* | 1 | 1 |
|  |  |  |  |  | *Justicia* | 8 | 23 |
|  |  |  |  |  | *Mendoncia* | 2 | 2 |
|  |  |  |  |  | *Odontonema* | 1 | 2 |
|  |  |  |  |  | *Pachystachys* | 4 | 19 |
|  |  |  |  |  | *Razisea* | 2 | 5 |
|  |  |  |  |  | *Ruellia* | 4 | 7 |
|  |  |  |  |  | *Sanchezia* | 3 | 10 |
|  |  |  |  |  | *Strobilanthes* | 1 | 2 |
|  |  |  |  |  | *Thunbergia* | 3 | 11 |
|  |  |  |  |  | *Trichanthera* | 2 | 9 |
| Alstroemeriaceae | 44 | 37 | 7 | O | *Alstroemeria* | 3 | 6 |
|  |  |  |  |  | *Bomarea* | 8 | 38 |
| Amaryllidaceae | 5 | 5 | 4 | I | *Hippeastrum* | 2 | 2 |
|  |  |  |  |  | *Manfreda* | 1 | 1 |
| Anacardiaceae | 10 | 9 | 3 | NT | *Anacardium* | 2 | 6 |
|  |  |  |  |  | *Toxicodendron* | 1 | 3 |
| Apocynaceae | 42 | 30 | 7 | I | *Allamanda* | 2 | 2 |
|  |  |  |  |  | *Asclepias* | 3 | 10 |
|  |  |  |  |  | *Mandevilla* | 6 | 13 |
|  |  |  |  |  | *Nerium* | 1 | 1 |
|  |  |  |  |  | *Prestonia* | 1 | 5 |
|  |  |  |  |  | *Tabernaemontana* | 3 | 6 |
|  |  |  |  |  | *Thevetia* | 1 | 1 |
|  |  |  |  |  | *Urechites* | 1 | 2 |
| Araliaceae | 4 | 2 | 1 | NT | *Dendropanax* | 1 | 2 |
|  |  |  |  |  | *Schefflera* | 1 | 2 |
| Asparagaceae | 65 | 29 | 5 | I | *Agave* | 15 | 60 |
|  |  |  |  |  | *Bessera* | 1 | 1 |
|  |  |  |  |  | *Dracaena* | 1 | 1 |
|  |  |  |  |  | *Taetsia* | 1 | 1 |
|  |  |  |  |  | *Yucca* | 1 | 1 |
| Asteraceae | 119 | 63 | 9 | NT | *Ageratum* | 1 | 1 |
|  |  |  |  |  | *Aspilia* | 2 | 3 |
|  |  |  |  |  | *Barnadesia* | 2 | 10 |
|  |  |  |  |  | *Bidens* | 2 | 2 |
|  |  |  |  |  | *Chuquiraga* | 3 | 5 |
|  |  |  |  |  | *Cirsium* | 9 | 31 |
|  |  |  |  |  | *Clibadium* | 1 | 1 |
|  |  |  |  |  | *Dasyphyllum* | 2 | 4 |
|  |  |  |  |  | *Dendroseris* | 2 | 2 |
|  |  |  |  |  | *Eremanthus* | 2 | 7 |
|  |  |  |  |  | *Espeletia* | 2 | 5 |
|  |  |  |  |  | *Gynoxys* | 1 | 1 |

**S1 Table** (continued)

| **FAMILY** | **INTER.FAM** | **HUMM.FAM** | **CLAD.FAM** | **SYND** | **GENUS** | **SPECIES** | **INTER.GEN** |
| --- | --- | --- | --- | --- | --- | --- | --- |
| Asteraceae |  |  |  |  | *Heliomeris* | 1 | 1 |
|  |  |  |  |  | *Hololepis* | 1 | 1 |
|  |  |  |  |  | *Lagascea* | 1 | 2 |
|  |  |  |  |  | *Lepidaploa* | 1 | 1 |
|  |  |  |  |  | *Lessingianthus* | 1 | 1 |
|  |  |  |  |  | *Mutisia* | 3 | 11 |
|  |  |  |  |  | *Piptocarpha* | 2 | 2 |
|  |  |  |  |  | *Roldana* | 1 | 5 |
|  |  |  |  |  | *Stifftia* | 1 | 7 |
|  |  |  |  |  | *Vernonanthura* | 1 | 5 |
|  |  |  |  |  | *Vernonia* | 2 | 3 |
|  |  |  |  |  | *Zinnia* | 1 | 1 |
| Balsaminaceae | 4 | 4 | 3 | NT | *Impatiens* | 2 | 4 |
| Begoniaceae | 1 | 1 | 1 | NT | *Begonia* | 1 | 1 |
| Berberidaceae | 9 | 9 | 3 | NT | *Berberis* | 2 | 9 |
| Betulaceae | 1 | 1 | 1 | NT | *Alnus* | 1 | 1 |
| Bignoniaceae | 128 | 47 | 7 | I | *Arrabidaea* | 1 | 2 |
|  |  |  |  |  | *Bignonia* | 1 | 1 |
|  |  |  |  |  | *Campsis* | 2 | 3 |
|  |  |  |  |  | *Chilopsis* | 2 | 7 |
|  |  |  |  |  | *Clytostoma* | 1 | 1 |
|  |  |  |  |  | *Dolichandra* | 1 | 4 |
|  |  |  |  |  | *Fridericia* | 1 | 2 |
|  |  |  |  |  | *Handroanthus* | 2 | 8 |
|  |  |  |  |  | *Jacaranda* | 1 | 2 |
|  |  |  |  |  | *Memora* | 1 | 2 |
|  |  |  |  |  | *Piriadacus* | 1 | 4 |
|  |  |  |  |  | *Pleonotoma* | 1 | 1 |
|  |  |  |  |  | *Pyrostegia* | 1 | 5 |
|  |  |  |  |  | *Saritaea* | 1 | 2 |
|  |  |  |  |  | *Setilobus* | 1 | 7 |
|  |  |  |  |  | *Spathodea* | 2 | 20 |
|  |  |  |  |  | *Tabebuia* | 11 | 39 |
|  |  |  |  |  | *Tecoma* | 3 | 9 |
|  |  |  |  |  | *Zeyheria* | 1 | 1 |
| Bonnetiaceae | 1 | 1 | 1 | NT | *Bonnetia* | 1 | 1 |
| Boraginaceae | 48 | 32 | 6 | I | *Bourreria* | 2 | 3 |
|  |  |  |  |  | *Cordia* | 12 | 35 |
|  |  |  |  |  | *Cynoglossum* | 1 | 1 |
|  |  |  |  |  | *Ehretia* | 2 | 2 |
|  |  |  |  |  | *Hydrophyllum* | 1 | 1 |
|  |  |  |  |  | *Lithospermum* | 1 | 1 |
|  |  |  |  |  | *Macromeria* | 1 | 1 |
|  |  |  |  |  | *Mertensia* | 1 | 1 |
|  |  |  |  |  | *Phacelia* | 1 | 1 |
|  |  |  |  |  | *Turricula* | 1 | 1 |
|  |  |  |  |  | *Wigandia* | 1 | 1 |
| Brassicaceae | 6 | 6 | 2 | I | *Draba* | 2 | 2 |
|  |  |  |  |  | *Nasturtium* | 1 | 1 |
|  |  |  |  |  | *Stanleya* | 1 | 3 |
| Bromeliaceae | 244 | 113 | 9 | O | *Aechmea* | 7 | 29 |
|  |  |  |  |  | *Billbergia* | 4 | 7 |
|  |  |  |  |  | *Bromelia* | 2 | 8 |
|  |  |  |  |  | *Canistrum* | 1 | 1 |
|  |  |  |  |  | *Dyckia* | 1 | 1 |

**S1 Table** (continued)

| **FAMILY** | **INTER.FAM** | **HUMM.FAM** | **CLAD.FAM** | **SYND** | **GENUS** | **SPECIES** | **INTER.GEN** |
| --- | --- | --- | --- | --- | --- | --- | --- |
| Bromeliaceae |  |  |  |  | *Guzmania* | 15 | 46 |
|  |  |  |  |  | *Hohenbergia* | 3 | 5 |
|  |  |  |  |  | *Mezobromelia* | 1 | 1 |
|  |  |  |  |  | *Neoregelia* | 2 | 3 |
|  |  |  |  |  | *Nidularium* | 2 | 4 |
|  |  |  |  |  | *Orthophytum* | 1 | 2 |
|  |  |  |  |  | *Pitcairnia* | 9 | 21 |
|  |  |  |  |  | *Puya* | 2 | 16 |
|  |  |  |  |  | *Quesnelia* | 3 | 7 |
|  |  |  |  |  | *Racinaea* | 2 | 2 |
|  |  |  |  |  | *Tillandsia* | 16 | 46 |
|  |  |  |  |  | *Vriesea* | 9 | 20 |
|  |  |  |  |  | *Werauhia* | 1 | 1 |
| Cactaceae | 64 | 39 | 5 | NT | *Carnegiea* | 1 | 1 |
|  |  |  |  |  | *Cereus* | 1 | 1 |
|  |  |  |  |  | *Cylindropuntia* | 2 | 2 |
|  |  |  |  |  | *Echinocereus* | 1 | 4 |
|  |  |  |  |  | *Lemaireocereus* | 2 | 7 |
|  |  |  |  |  | *Melocactus* | 2 | 9 |
|  |  |  |  |  | *Neobuxbaumia* | 1 | 4 |
|  |  |  |  |  | *Opuntia* | 7 | 26 |
|  |  |  |  |  | *Stenocereus* | 2 | 2 |
| Calophyllaceae | 1 | 1 | 1 | NT | *Calophyllum* | 1 | 1 |
| Calycanthaceae | 1 | 1 | 1 | NT | *Beureria* | 1 | 1 |
| Campanulaceae | 121 | 75 | 8 | O | *Burmeistera* | 5 | 5 |
|  |  |  |  |  | *Centropogon* | 12 | 56 |
|  |  |  |  |  | *Lobelia* | 5 | 37 |
|  |  |  |  |  | *Siphocampylus* | 7 | 17 |
| Cannaceae | 9 | 7 | 3 | I | *Canna* | 2 | 8 |
| Caprifoliaceae | 19 | 10 | 3 | I | *Lonicera* | 7 | 15 |
|  |  |  |  |  | *Symphoricarpos* | 3 | 4 |
| Caricaceae | 4 | 4 | 2 | I | *Carica* | 1 | 4 |
| Caryophyllaceae | 8 | 5 | 2 | I | *Silene* | 5 | 8 |
| Chrysobalanaceae | 1 | 1 | 1 | NT | *Hirtella* | 1 | 1 |
| Cleomaceae | 5 | 4 | 1 | NT | *Cleome* | 2 | 3 |
|  |  |  |  |  | *Peritoma* | 2 | 2 |
| Clethraceae | 5 | 5 | 3 | NT | *Clethra* | 2 | 5 |
| Clusiaceae | 40 | 33 | 6 | NT | *Clusia* | 4 | 27 |
|  |  |  |  |  | *Symphonia* | 2 | 13 |
| Columelliaceae | 1 | 1 | 1 | O | *Desfontainia* | 1 | 1 |
| Combretaceae | 22 | 18 | 4 | NT | *Combretum* | 5 | 18 |
|  |  |  |  |  | *Laguncularia* | 2 | 4 |
| Convolvulaceae | 51 | 32 | 4 | I | *Bonamia* | 1 | 1 |
|  |  |  |  |  | *Convolvulus* | 1 | 2 |
|  |  |  |  |  | *Exogonium* | 1 | 3 |
|  |  |  |  |  | *Ipomoea* | 10 | 39 |
|  |  |  |  |  | *Jacquemontia* | 1 | 2 |
| Costaceae | 51 | 34 | 6 | O | *Costus* | 8 | 51 |
| Crassulaceae | 13 | 12 | 4 | I | *Dudleya* | 2 | 2 |
|  |  |  |  |  | *Echeveria* | 4 | 6 |
|  |  |  |  |  | *Kalanchoe* | 3 | 4 |
| Cucurbitaceae | 30 | 19 | 4 | I | *Gurania* | 5 | 30 |
| Elaeocarpaceae | 5 | 5 | 1 | NT | *Vallea* | 1 | 5 |
| Ericaceae | 238 | 95 | 8 | O | *Agarista* | 2 | 2 |
|  |  |  |  |  | *Arbutus* | 3 | 4 |

**S1 Table** (continued)

| **FAMILY** | **INTER.FAM** | **HUMM.FAM** | **CLAD.FAM** | **SYND** | **GENUS** | **SPECIES** | **INTER.GEN** |
| --- | --- | --- | --- | --- | --- | --- | --- |
| Ericaceae |  |  |  |  | *Arctostaphylos* | 4 | 8 |
|  |  |  |  |  | *Bejaria* | 1 | 5 |
|  |  |  |  |  | *Cavendishia* | 9 | 61 |
|  |  |  |  |  | *Comarostaphylis* | 1 | 1 |
|  |  |  |  |  | *Disterigma* | 1 | 8 |
|  |  |  |  |  | *Gaultheria* | 6 | 10 |
|  |  |  |  |  | *Gaylussacia* | 1 | 1 |
|  |  |  |  |  | *Killipiella* | 1 | 2 |
|  |  |  |  |  | *Macleania* | 4 | 37 |
|  |  |  |  |  | *Menziesia* | 1 | 1 |
|  |  |  |  |  | *Pernettya* | 1 | 2 |
|  |  |  |  |  | *Psammisia* | 5 | 26 |
|  |  |  |  |  | *Sarcodes* | 1 | 1 |
|  |  |  |  |  | *Satyria* | 2 | 8 |
|  |  |  |  |  | *Sphyrospermum* | 1 | 2 |
|  |  |  |  |  | *Thibaudia* | 4 | 10 |
|  |  |  |  |  | *Vaccinium* | 3 | 9 |
| Escalloniaceae | 1 | 1 | 1 | I | *Escallonia* | 1 | 1 |
| Euphorbiaceae | 26 | 20 | 6 | NT | *Acalypha* | 2 | 3 |
|  |  |  |  |  | *Croton* | 3 | 3 |
|  |  |  |  |  | *Euphorbia* | 7 | 13 |
|  |  |  |  |  | *Jatropha* | 1 | 1 |
|  |  |  |  |  | *Manihot* | 1 | 1 |
|  |  |  |  |  | *Pedilanthus* | 1 | 2 |
|  |  |  |  |  | *Poinsettia* | 1 | 1 |
| Fabaceae | 482 | 174 | 8 | NT | *Abarema* | 1 | 3 |
|  |  |  |  |  | *Acacia* | 3 | 5 |
|  |  |  |  |  | *Acrocarpus* | 1 | 2 |
|  |  |  |  |  | *Anneslia* | 1 | 1 |
|  |  |  |  |  | *Bauhinia* | 6 | 27 |
|  |  |  |  |  | *Brownea* | 2 | 4 |
|  |  |  |  |  | *Caesalpinia* | 4 | 21 |
|  |  |  |  |  | *Cajanus* | 2 | 3 |
|  |  |  |  |  | *Calliandra* | 14 | 55 |
|  |  |  |  |  | *Calopogonium* | 1 | 1 |
|  |  |  |  |  | *Camptosema* | 1 | 1 |
|  |  |  |  |  | *Canavalia* | 2 | 5 |
|  |  |  |  |  | *Caragana* | 1 | 1 |
|  |  |  |  |  | *Cassia* | 1 | 2 |
|  |  |  |  |  | *Centrosema* | 2 | 3 |
|  |  |  |  |  | *Cercidium* | 1 | 1 |
|  |  |  |  |  | *Cercis* | 2 | 2 |
|  |  |  |  |  | *Chaetocalyx* | 1 | 1 |
|  |  |  |  |  | *Chloroleucon* | 1 | 2 |
|  |  |  |  |  | *Clathrotropis* | 1 | 2 |
|  |  |  |  |  | *Clitoria* | 1 | 1 |
|  |  |  |  |  | *Collaea* | 2 | 2 |
|  |  |  |  |  | *Crotalaria* | 3 | 6 |
|  |  |  |  |  | *Dahlstedtia* | 2 | 8 |
|  |  |  |  |  | *Delonix* | 2 | 13 |
|  |  |  |  |  | *Dioclea* | 2 | 3 |
|  |  |  |  |  | *Dipteryx* | 1 | 1 |
|  |  |  |  |  | *Ebenopsis* | 1 | 1 |
|  |  |  |  |  | *Erythrina* | 12 | 85 |
|  |  |  |  |  | *Gliricidia* | 1 | 3 |

**S1 Table** (continued)

| **FAMILY** | **INTER.FAM** | **HUMM.FAM** | **CLAD.FAM** | **SYND** | **GENUS** | **SPECIES** | **INTER.GEN** |
| --- | --- | --- | --- | --- | --- | --- | --- |
| Fabaceae |  |  |  |  | *Havardia* | 1 | 1 |
|  |  |  |  |  | *Hymenolobium* | 1 | 1 |
|  |  |  |  |  | *Inga* | 10 | 109 |
|  |  |  |  |  | *Leucaena* | 2 | 2 |
|  |  |  |  |  | *Lonchocarpus* | 1 | 2 |
|  |  |  |  |  | *Lotus* | 1 | 1 |
|  |  |  |  |  | *Lupinus* | 3 | 7 |
|  |  |  |  |  | *Mimosa* | 2 | 4 |
|  |  |  |  |  | *Mucuna* | 2 | 3 |
|  |  |  |  |  | *Neorudolphia* | 1 | 4 |
|  |  |  |  |  | *Olneya* | 2 | 2 |
|  |  |  |  |  | *Parkinsonia* | 1 | 1 |
|  |  |  |  |  | *Periandra* | 1 | 2 |
|  |  |  |  |  | *Phaseolus* | 2 | 14 |
|  |  |  |  |  | *Pithecellobium* | 1 | 7 |
|  |  |  |  |  | *Prosopis* | 2 | 3 |
|  |  |  |  |  | *Robinia* | 2 | 3 |
|  |  |  |  |  | *Sabinea* | 2 | 4 |
|  |  |  |  |  | *Samanea* | 2 | 10 |
|  |  |  |  |  | *Sophora* | 1 | 1 |
|  |  |  |  |  | *Strongylodon* | 1 | 1 |
|  |  |  |  |  | *Tachigali* | 1 | 1 |
|  |  |  |  |  | *Tamarindus* | 2 | 4 |
|  |  |  |  |  | *Vicia* | 1 | 2 |
|  |  |  |  |  | *Vigna* | 1 | 1 |
| Fouquieriaceae | 18 | 9 | 2 | O | *Fouquieria* | 5 | 18 |
| Gentianaceae | 15 | 12 | 5 | I | *Chelonanthus* | 1 | 3 |
|  |  |  |  |  | *Gentiana* | 1 | 2 |
|  |  |  |  |  | *Lisianthius* | 1 | 1 |
|  |  |  |  |  | *Macrocarpaea* | 3 | 5 |
|  |  |  |  |  | *Swertia* | 1 | 1 |
|  |  |  |  |  | *Symbolanthus* | 2 | 3 |
| Geraniaceae | 2 | 2 | 2 | NT | *Geranium* | 2 | 2 |
| Gesneriaceae | 240 | 81 | 8 | O | *Alloplectus* | 1 | 9 |
|  |  |  |  |  | *Besleria* | 8 | 56 |
|  |  |  |  |  | *Columnea* | 9 | 37 |
|  |  |  |  |  | *Drymonia* | 8 | 24 |
|  |  |  |  |  | *Gasteranthus* | 2 | 3 |
|  |  |  |  |  | *Gesneria* | 1 | 1 |
|  |  |  |  |  | *Glossoloma* | 3 | 12 |
|  |  |  |  |  | *Heppiella* | 1 | 4 |
|  |  |  |  |  | *Kohleria* | 8 | 45 |
|  |  |  |  |  | *Moussonia* | 1 | 1 |
|  |  |  |  |  | *Nematanthus* | 4 | 10 |
|  |  |  |  |  | *Paliavana* | 1 | 4 |
|  |  |  |  |  | *Rechsteineria* | 1 | 1 |
|  |  |  |  |  | *Reldia* | 1 | 2 |
|  |  |  |  |  | *Rhytidophyllum* | 1 | 1 |
|  |  |  |  |  | *Sinningia* | 4 | 7 |
| Grossulariaceae | 24 | 14 | 5 | I | *Escallonia* | 1 | 1 |
|  |  |  |  |  | *Ribes* | 7 | 23 |
| Heliconiaceae | 183 | 83 | 8 | O | *Heliconia* | 34 | 176 |
| Hypericaceae | 2 | 2 | 2 | NT | *Vismia* | 1 | 2 |
| Iridaceae | 9 | 8 | 3 | NT | *Gladiolus* | 1 | 1 |
|  |  |  |  |  | *Iris* | 2 | 2 |

**S1 Table** (continued)

| **FAMILY** | **INTER.FAM** | **HUMM.FAM** | **CLAD.FAM** | **SYND** | **GENUS** | **SPECIES** | **INTER.GEN** |
| --- | --- | --- | --- | --- | --- | --- | --- |
| Iridaceae |  |  |  |  | *Tigridia* | 1 | 6 |
| Lamiaceae | 204 | 83 | 8 | I | *Aegiphila* | 2 | 3 |
|  |  |  |  |  | *Amasonia* | 1 | 3 |
|  |  |  |  |  | *Clinopodium* | 1 | 1 |
|  |  |  |  |  | *Coleus* | 1 | 2 |
|  |  |  |  |  | *Condea* | 1 | 2 |
|  |  |  |  |  | *Cornutia* | 2 | 2 |
|  |  |  |  |  | *Holmskioldia* | 2 | 2 |
|  |  |  |  |  | *Hyptis* | 1 | 2 |
|  |  |  |  |  | *Leonotis* | 3 | 9 |
|  |  |  |  |  | *Leonurus* | 2 | 3 |
|  |  |  |  |  | *Lepechinia* | 1 | 1 |
|  |  |  |  |  | *Leptohyptis* | 1 | 2 |
|  |  |  |  |  | *Monarda* | 4 | 4 |
|  |  |  |  |  | *Monardella* | 1 | 1 |
|  |  |  |  |  | *Origanum* | 1 | 1 |
|  |  |  |  |  | *Plectranthus* | 1 | 1 |
|  |  |  |  |  | *Prunella* | 1 | 5 |
|  |  |  |  |  | *Rhabdocaulon* | 1 | 1 |
|  |  |  |  |  | *Salvia* | 28 | 121 |
|  |  |  |  |  | *Satureja* | 1 | 1 |
|  |  |  |  |  | *Scutellaria* | 2 | 2 |
|  |  |  |  |  | *Stachys* | 4 | 19 |
|  |  |  |  |  | *Trichostema* | 3 | 3 |
|  |  |  |  |  | *Vitex* | 4 | 8 |
| Lecythidaceae | 3 | 3 | 2 | NT | *Bertholletia* | 1 | 2 |
|  |  |  |  |  | *Couroupita* | 1 | 1 |
| Liliaceae | 6 | 3 | 2 | I | *Erythronium* | 1 | 2 |
|  |  |  |  |  | *Lilium* | 2 | 2 |
| Loasaceae | 7 | 6 | 3 | NT | *Caiophora* | 1 | 3 |
|  |  |  |  |  | *Eucnide* | 1 | 1 |
|  |  |  |  |  | *Loasa* | 1 | 3 |
| Loganiaceae | 1 | 1 | 1 | NT |  | 0 | 1 |
| Loranthaceae | 93 | 70 | 8 | O | *Aetanthus* | 2 | 5 |
|  |  |  |  |  | *Gaiadendron* | 2 | 4 |
|  |  |  |  |  | *Phrygilanthus* | 1 | 1 |
|  |  |  |  |  | *Psittacanthus* | 9 | 56 |
|  |  |  |  |  | *Struthanthus* | 1 | 1 |
|  |  |  |  |  | *Tripodanthus* | 1 | 1 |
|  |  |  |  |  | *Tristerix* | 2 | 6 |
| Lythraceae | 28 | 23 | 7 | I | *Cuphea* | 7 | 23 |
|  |  |  |  |  | *Lafoensia* | 2 | 2 |
|  |  |  |  |  | *Lagerstroemia* | 2 | 2 |
|  |  |  |  |  | *Punica* | 1 | 1 |
| Malvaceae | 201 | 102 | 8 | NT | *Abutilon* | 2 | 10 |
|  |  |  |  |  | *Bombax* | 1 | 1 |
|  |  |  |  |  | *Byttneria* | 1 | 2 |
|  |  |  |  |  | *Ceiba* | 2 | 9 |
|  |  |  |  |  | *Chiranthodendron* | 1 | 1 |
|  |  |  |  |  | *Dombeya* | 2 | 7 |
|  |  |  |  |  | *Goethea* | 1 | 1 |
|  |  |  |  |  | *Hampea* | 2 | 5 |
|  |  |  |  |  | *Helicteres* | 4 | 18 |
|  |  |  |  |  | *Hibiscus* | 4 | 47 |
|  |  |  |  |  | *Luehea* | 2 | 2 |

**S1 Table** (continued)

| **FAMILY** | **INTER.FAM** | **HUMM.FAM** | **CLAD.FAM** | **SYND** | **GENUS** | **SPECIES** | **INTER.GEN** |
| --- | --- | --- | --- | --- | --- | --- | --- |
| Malvaceae |  |  |  |  | *Malva* | 1 | 1 |
|  |  |  |  |  | *Malvaviscus* | 3 | 47 |
|  |  |  |  |  | *Melochia* | 1 | 1 |
|  |  |  |  |  | *Pachira* | 1 | 2 |
|  |  |  |  |  | *Pavonia* | 3 | 12 |
|  |  |  |  |  | *Pseudobombax* | 2 | 2 |
|  |  |  |  |  | *Quararibea* | 1 | 5 |
|  |  |  |  |  | *Trichospermum* | 1 | 1 |
|  |  |  |  |  | *Triumfetta* | 1 | 1 |
|  |  |  |  |  | *Urena* | 1 | 2 |
| Marantaceae | 20 | 15 | 6 | I | *Calathea* | 5 | 5 |
|  |  |  |  |  | *Ischnosiphon* | 2 | 2 |
|  |  |  |  |  | *Maranta* | 1 | 1 |
|  |  |  |  |  | *Monotagma* | 2 | 2 |
|  |  |  |  |  | *Saranthe* | 1 | 1 |
|  |  |  |  |  | *Thalia* | 2 | 5 |
| Marcgraviaceae | 37 | 25 | 8 | NT | *Marcgravia* | 8 | 16 |
|  |  |  |  |  | *Marcgraviastrum* | 1 | 1 |
|  |  |  |  |  | *Norantea* | 2 | 10 |
|  |  |  |  |  | *Sarcopera* | 1 | 5 |
| Melastomataceae | 53 | 35 | 5 | NT | *Brachyotum* | 3 | 14 |
|  |  |  |  |  | *Conostegia* | 2 | 2 |
|  |  |  |  |  | *Heterotrichum* | 1 | 1 |
|  |  |  |  |  | *Huilaea* | 2 | 3 |
|  |  |  |  |  | *Mecranium* | 1 | 1 |
|  |  |  |  |  | *Meriania* | 3 | 4 |
|  |  |  |  |  | *Miconia* | 6 | 19 |
|  |  |  |  |  | *Tibouchina* | 1 | 2 |
| Meliaceae | 6 | 6 | 3 | NT | *Cedrela* | 1 | 1 |
|  |  |  |  |  | *Guarea* | 2 | 2 |
|  |  |  |  |  | *Melia* | 1 | 3 |
| Moraceae | 1 | 1 | 1 | NT | *Ficus* | 1 | 1 |
| Muntingiaceae | 2 | 2 | 1 | NT | *Muntingia* | 1 | 2 |
| Musaceae | 32 | 30 | 6 | O | *Musa* | 4 | 29 |
| Myrtaceae | 79 | 60 | 9 | NT | *Callistemon* | 2 | 4 |
|  |  |  |  |  | *Eucalyptus* | 3 | 36 |
|  |  |  |  |  | *Eugenia* | 2 | 8 |
|  |  |  |  |  | *Myrciaria* | 1 | 1 |
|  |  |  |  |  | *Psidium* | 2 | 3 |
|  |  |  |  |  | *Syzygium* | 3 | 15 |
| Nyctaginaceae | 10 | 10 | 2 | I | *Bougainvillea* | 3 | 5 |
|  |  |  |  |  | *Mirabilis* | 2 | 5 |
| Ochnaceae | 1 | 1 | 1 | NT | *Tyleria* | 1 | 1 |
| Oleaceae | 2 | 1 | 1 | I | *Nyctanthes* | 2 | 2 |
| Onagraceae | 82 | 53 | 7 | I | *Epilobium* | 4 | 10 |
|  |  |  |  |  | *Fuchsia* | 12 | 67 |
|  |  |  |  |  | *Ludwigia* | 1 | 1 |
|  |  |  |  |  | *Zauschneria* | 1 | 1 |
| Orchidaceae | 27 | 18 | 6 | NT | *Dilomilis* | 1 | 1 |
|  |  |  |  |  | *Elleanthus* | 4 | 15 |
|  |  |  |  |  | *Epidendrum* | 1 | 2 |
|  |  |  |  |  | *Odontoglossum* | 1 | 1 |
|  |  |  |  |  | *Prosthechea* | 1 | 1 |
|  |  |  |  |  | *Rhynchostele* | 1 | 1 |
|  |  |  |  |  | *Spiranthes* | 1 | 1 |

**S1 Table** (continued)

| **FAMILY** | **INTER.FAM** | **HUMM.FAM** | **CLAD.FAM** | **SYND** | **GENUS** | **SPECIES** | **INTER.GEN** |
| --- | --- | --- | --- | --- | --- | --- | --- |
| Orchidaceae |  |  |  |  | *Stenorrhynchos* | 1 | 3 |
| Orobanchaceae | 75 | 34 | 6 | O | *Agalinis* | 1 | 1 |
|  |  |  |  |  | *Bartsia* | 1 | 1 |
|  |  |  |  |  | *Castilleja* | 18 | 59 |
|  |  |  |  |  | *Esterhazya* | 2 | 2 |
|  |  |  |  |  | *Lamourouxia* | 4 | 7 |
|  |  |  |  |  | *Macranthera* | 2 | 2 |
|  |  |  |  |  | *Pedicularis* | 2 | 3 |
| Papaveraceae | 1 | 1 | 1 | I | *Corydalis* | 1 | 1 |
| Passifloraceae | 59 | 41 | 7 | NT | *Passiflora* | 19 | 51 |
| Phrymaceae | 18 | 8 | 3 | I | *Diplacus* | 2 | 4 |
|  |  |  |  |  | *Erythranthe* | 1 | 1 |
|  |  |  |  |  | *Mimulus* | 5 | 13 |
| Phyllanthaceae | 1 | 1 | 1 | NT | *Phyllanthus* | 1 | 1 |
| Pittosporaceae | 3 | 3 | 2 | NT | *Pittosporum* | 1 | 3 |
| Plantaginaceae | 95 | 39 | 4 | O | *Digitalis* | 2 | 2 |
|  |  |  |  |  | *Keckiella* | 3 | 4 |
|  |  |  |  |  | *Linaria* | 2 | 2 |
|  |  |  |  |  | *Maurandya* | 1 | 1 |
|  |  |  |  |  | *Penstemon* | 17 | 64 |
|  |  |  |  |  | *Plantago* | 1 | 1 |
|  |  |  |  |  | *Russelia* | 3 | 21 |
| Plumbaginaceae | 4 | 3 | 2 | I | *Plumbago* | 2 | 4 |
| Polemoniaceae | 18 | 11 | 3 | I | *Gilia* | 1 | 3 |
|  |  |  |  |  | *Ipomopsis* | 2 | 7 |
|  |  |  |  |  | *Loeselia* | 1 | 7 |
|  |  |  |  |  | *Polemonium* | 1 | 1 |
| Polygalaceae | 2 | 1 | 1 | I | *Securidaca* | 2 | 2 |
| Polygonaceae | 4 | 4 | 2 | NT | *Antigonon* | 2 | 3 |
|  |  |  |  |  | *Coccoloba* | 1 | 1 |
| Proteaceae | 7 | 6 | 4 | O | *Embothrium* | 2 | 5 |
|  |  |  |  |  | *Grevillea* | 1 | 1 |
| Ranunculaceae | 33 | 12 | 2 | I | *Aconitum* | 1 | 1 |
|  |  |  |  |  | *Aquilegia* | 6 | 15 |
|  |  |  |  |  | *Delphinium* | 7 | 17 |
| Rhamnaceae | 1 | 1 | 1 | I | *Ceanothus* | 1 | 1 |
| Rosaceae | 27 | 22 | 5 | NT | *Eriobotrya* | 1 | 1 |
|  |  |  |  |  | *Hesperomeles* | 1 | 2 |
|  |  |  |  |  | *Polylepis* | 1 | 2 |
|  |  |  |  |  | *Prunus* | 1 | 2 |
|  |  |  |  |  | *Rubus* | 6 | 20 |
| Rubiaceae | 384 | 154 | 8 | I | *Aegiphila* | 1 | 2 |
|  |  |  |  |  | *Augusta* | 1 | 1 |
|  |  |  |  |  | *Bouvardia* | 2 | 18 |
|  |  |  |  |  | *Calycophyllum* | 1 | 1 |
|  |  |  |  |  | *Cinchona* | 1 | 1 |
|  |  |  |  |  | *Coffea* | 2 | 9 |
|  |  |  |  |  | *Coussarea* | 1 | 3 |
|  |  |  |  |  | *Crusea* | 1 | 8 |
|  |  |  |  |  | *Duggena* | 1 | 4 |
|  |  |  |  |  | *Duroia* | 1 | 5 |
|  |  |  |  |  | *Ernodea* | 1 | 1 |
|  |  |  |  |  | *Faramea* | 2 | 7 |
|  |  |  |  |  | *Genipa* | 2 | 14 |
|  |  |  |  |  | *Hamelia* | 4 | 53 |

**S1 Table** (continued)

| **FAMILY** | **INTER.FAM** | **HUMM.FAM** | **CLAD.FAM** | **SYND** | **GENUS** | **SPECIES** | **INTER.GEN** |
| --- | --- | --- | --- | --- | --- | --- | --- |
| Rubiaceae |  |  |  |  | *Hillia* | 1 | 1 |
|  |  |  |  |  | *Isertia* | 2 | 9 |
|  |  |  |  |  | *Ixora* | 3 | 8 |
|  |  |  |  |  | *Manettia* | 4 | 10 |
|  |  |  |  |  | *Morinda* | 2 | 2 |
|  |  |  |  |  | *Palicourea* | 16 | 111 |
|  |  |  |  |  | *Pentas* | 1 | 1 |
|  |  |  |  |  | *Posoqueria* | 1 | 1 |
|  |  |  |  |  | *Psychotria* | 12 | 59 |
|  |  |  |  |  | *Rondeletia* | 1 | 1 |
|  |  |  |  |  | *Sabicea* | 2 | 3 |
|  |  |  |  |  | *Simira* | 1 | 1 |
|  |  |  |  |  | *Sommera* | 1 | 1 |
|  |  |  |  |  | *Warszewiczia* | 2 | 13 |
| Rutaceae | 27 | 27 | 6 | NT | *Citrus* | 2 | 19 |
|  |  |  |  |  | *Decagonocarpus* | 1 | 2 |
|  |  |  |  |  | *Murraya* | 1 | 1 |
| Salicaceae | 6 | 6 | 4 | NT | *Ryania* | 1 | 4 |
|  |  |  |  |  | *Salix* | 1 | 2 |
| Sapindaceae | 16 | 10 | 3 | NT | *Aesculus* | 3 | 4 |
|  |  |  |  |  | *Paullinia* | 3 | 5 |
|  |  |  |  |  | *Serjania* | 2 | 5 |
|  |  |  |  |  | *Ungnadia* | 2 | 2 |
| Sapotaceae | 5 | 5 | 2 | NT | *Bumelia* | 1 | 5 |
| Saxifragaceae | 2 | 2 | 2 | NT | *Heuchera* | 2 | 2 |
| Scrophulariaceae | 16 | 12 | 6 | NT | *Buddleja* | 4 | 12 |
|  |  |  |  |  | *Scrophularia* | 3 | 4 |
| Simaroubaceae | 2 | 2 | 2 | O | *Quassia* | 1 | 2 |
| Solanaceae | 63 | 40 | 8 | I | *Acnistus* | 2 | 4 |
|  |  |  |  |  | *Brugmansia* | 1 | 1 |
|  |  |  |  |  | *Cestrum* | 5 | 13 |
|  |  |  |  |  | *Datura* | 2 | 2 |
|  |  |  |  |  | *Dunalia* | 1 | 2 |
|  |  |  |  |  | *Dyssochroma* | 1 | 1 |
|  |  |  |  |  | *Iochroma* | 1 | 1 |
|  |  |  |  |  | *Lycium* | 5 | 6 |
|  |  |  |  |  | *Nicotiana* | 4 | 26 |
|  |  |  |  |  | *Salpichroa* | 1 | 2 |
|  |  |  |  |  | *Sessea* | 1 | 1 |
|  |  |  |  |  | *Solanum* | 2 | 2 |
|  |  |  |  |  | *Streptosolen* | 1 | 1 |
|  |  |  |  |  | *Witheringia* | 1 | 1 |
| Strelitziaceae | 2 | 2 | 2 | I | *Strelitzia* | 1 | 1 |
| Symplocaceae | 1 | 1 | 1 | NT | *Symplocos* | 1 | 1 |
| Tetrameristaceae | 4 | 3 | 1 | NT | *Pelliciera* | 2 | 4 |
| Tropaeolaceae | 5 | 5 | 3 | I | *Tropaeolum* | 1 | 5 |
| Urticaceae | 6 | 6 | 3 | NT | *Cecropia* | 2 | 5 |
| Velloziaceae | 7 | 3 | 2 | I | *Barbacenia* | 5 | 6 |
| Verbenaceae | 101 | 67 | 6 | I | *Citharexylum* | 1 | 1 |
|  |  |  |  |  | *Duranta* | 3 | 9 |
|  |  |  |  |  | *Holmskioldia* | 1 | 2 |
|  |  |  |  |  | *Lantana* | 4 | 32 |
|  |  |  |  |  | *Lippia* | 2 | 3 |
|  |  |  |  |  | *Petrea* | 2 | 3 |
|  |  |  |  |  | *Rhaphithamnus* | 2 | 2 |

**S1 Table** (continued)

| **FAMILY** | **INTER.FAM** | **HUMM.FAM** | **CLAD.FAM** | **SYND** | **GENUS** | **SPECIES** | **INTER.GEN** |
| --- | --- | --- | --- | --- | --- | --- | --- |
| Verbenaceae |  |  |  |  | *Stachytarpheta* | 6 | 36 |
| Vitaceae | 1 | 1 | 1 | NT | *Cayratia* | 1 | 1 |
| Vochysiaceae | 23 | 20 | 6 | NT | *Vochysia* | 4 | 13 |
| Xanthorrhoeaceae | 7 | 6 | 2 | I | *Aloe* | 2 | 4 |
|  |  |  |  |  | *Kniphofia* | 1 | 2 |
|  |  |  |  |  | *Phormium* | 1 | 1 |
| Zingiberaceae | 35 | 30 | 5 | I | *Alpinia* | 2 | 7 |
|  |  |  |  |  | *Etlingera* | 1 | 2 |
|  |  |  |  |  | *Hedychium* | 1 | 3 |
|  |  |  |  |  | *Renealmia* | 3 | 16 |
| Zygophyllaceae | 2 | 1 | 1 | NT | *Larrea* | 2 | 2 |

**Supporting References**

1. Aldrich EC (1945) Nesting of the Allen Hummingbird. The Condor 47: 137–148.

2. Aldridge G, Campbell DR (2007) Variation in pollinator preference between two *Ipomopsis* contact sites that differ in hybridization rate. Evolution 61: 99–110.

3. Álvarez Vargas GP, Suarez Díaz N (2011) Riqueza y visitantes florales de la Familia Gesneriaceae en un bosque montano del Departamento del Quindío. Armenia: Universidad del Quindío. 58 p.

4. Amaya-Márquez M, Stiles FG, Rangel O (2001) Interacción planta-colibrí en Amacayacu (Amazonas, Colombia): Una perspectiva palinológica. Caldasia 23: 301–322.

5. Araujo AC, Fischer EA, Sazima M(1994) Floração seqüencial e polinização de três espécies de *Vriesea* (Bromeliaceae) na região da Juréia, sudeste do Brasil. Revista Brasileira de Botânica 17: 113–118.

6. Araujo AC, Sazima M (2003) The assemblage of flowers visited by hummingbirds in the "capões" of Southern Pantanal, Mato Grosso do Sul, Brazil. Flora - Morphology, Distribution, Functional Ecology of Plants 198: 427–435.

7. Arizmendi MC (2001) Multiple ecological interactions: nectar robbers and hummingbirds in a highland forest in Mexico. Canadian Journal of Zoology 79: 997–1006.

8. Arizmendi MC, Dominguez CA, Dirzo R (1996) The role of an avian nectar robber and of hummingbird pollinators in the reproduction of two plant species. Functional Ecology 10: 119–127.

9. Arizmendi MC, Ornelas JF (1990) Hummingbirds and their floral resources in a tropical dry forest in Mexico. Biotropica 22: 172–180.

10. Arizmendi MC, López-Saut E, Monterrubio-Solis C, Juarez L, Flores-Moreno I, et al. (2008) Efecto de la presencia de bebederos artificiales sobre la diversidad y abundancia de los colibríes y el éxito reproductivo de dos especies de plantas en un parque suburbano de la Ciudad de México. Ornitologia Neotropical 19: 491–500.

11. Arriaga L, Rodriguez-Estrella R, Ortega-Rubio A (1990) Endemic hummingbirds and madrones of Baja: Are they mutually dependent? The Southwestern Naturalist 35: 76–79.

12. Asociación Bogotana de Ornitología (2000) Aves de la Sabana de Bogotá, guía de campo. Bogotá: ABO, CAR.

13. Azpeitia F, Lara C (2006) Reproductive biology and pollination of the parasitic plant *Psittacanthus calyculatus* (Loranthaceae) in central Mexico. The Journal of the Torrey Botanical Society 133: 429–438.

14. Baltosser WH (1989) Nectar availability and habitat selection by hummingbirds in Guadalupe Canyon. The Wilson Bulletin 101: 559–578.

15. Baza Mendonça L, dos Anjos L (2005) Beija-flores (Aves, Trochilidae) e seus recursos florais em uma área urbana do Sul do Brasil. Revista Brasileira de Zoologia 22: 51–59.

16. Bent AC (1940) Life histories of North American Cuckoos, Goatsuckers, Hummingbirds, and their Allies. US National Museum Bulletin 176: 1-506.

17. Borgella RJ, Snow AA, Gavin TA (2001) Species richness and pollen loads of hummingbirds using forest fragments in southern Costa Rica. Biotropica 33: 90–109.

18. Bosch M, Waser NM (2001) Experimental manipulation of plant density and its effect on pollination and reproduction of two confamilial montane herbs. Oecologia 126: 76–83.

19. Buzato S, Sazima M, Sazima I (2000) Hummingbird-pollinated floras at three Atlantic forest sites1. Biotropica 32: 824–841.

20. Calviño Cancela M (2006) Time-activity budgets and behaviour of the Amazilia hummingbird (*Amazilia amazilia*, Apodiformes: Throchilidae) in an urban environment. Biología Tropical 54: 873–878.

21. Canela MBF, Sazima M (2003) Florivory by the crab *Armases angustipes* (Grapsidae) influences hummingbird visits to *Aechmea pectinata* (Bromeliaceae)1. Biotropica 35: 289–294.

22. Chaves-Portilla G, Cortés-Herrera O (2006) Nueva localidad para la Quincha de Soatá (*Amazilia castaneiventris*) en el Municipio de San Gil, Santander, Colombia. Boletín SAO 16: 01–06.

23. Clark CJ, Feo TJ, Escalante I (2011) Courtship displays and natural history of Scintillant (*Selasphorus scintilla*) and Volcano (*S. flammula*) Hummingbirds. The Wilson Journal of Ornithology 123: 218–228.

24. Cortés-Herrera O, Hernández-Jaramillo A, Briceño-Buitrago E (2004) Redescubrimiento del colibri *Amazilia castaneiventris*, una especie endémica y amenazada de Colombia. Ornitología Colombiana 02: 47–49.

25. Cotton PA (1998) Temporal partitioning of a floral resource by territorial hummingbirds. Ibis 140: 647–653.

26. Cotton PA (1998) The hummingbird community of a lowland Amazonian rainforest. Ibis 140: 512–521.

27. Cotton PA (2001) The behavior and interactions of birds visiting *Erythrina fusca* flowers in the Colombian Amazon. Biotropica 33: 662–669.

28. DeGraaf RM, Rappole JH (1995) Neotropical Migratory Birds. Natural History, Distribution, and Population Change. Ithaca and London: Comstock Publishing Associates. 676 p.

29. Des Granges J-L (1979) Organization of a tropical nectar feeding bird guild in a variable tropical environment. Living Bird 17: 199–236.

30. Des Granges J-L, Grant PR (1980) Migrant hummingbirds' accommodation into tropical communities. In: Keast A, Morton ES, editors. Migrant birds in the Neotropics: ecology, behavior, distribution, and conservation. Washington, D.C.: Smithsonian Institution Press. pp. 576.

31. Feinsinger P (1976) Organization of a tropical guild of nectarivorous birds. Ecological Monographs 46: 257–291.

32. Feinsinger P, Wolfe JA, Swarm LA (1982) Island ecology: reduced hummingbird diversity and the pollination biology of plants, Trinidad and Tobago, West Indies. Ecology 63: 494–506.

33. Ferreira VM, Lombardi JA (2001) Hummingbirds and their flowers in the campos rupestres of Southern Espinhaco Range, Brazil. Melopsittacus 4: 3–30.

34. Fetscher E, Rupert S, Kohn J (2002) Hummingbird foraging position is altered by the touch-sensitive stigma of bush monkeyflower. Oecologia 133: 551–558.

35. Fischer EA (1994) Polinização, fenologia e distribuição espacial de Bromeliaceae numa comunidade de Mata Atlântica, litoral sul de São Paulo. Campinas: Universidade Estadual de Campinas. 80 p.

36. Fischer EA, Araujo AC (1995) Spatial organization of a bromeliad community in the Atlantic rainforest, south-eastern Brazil. Journal of Tropical Ecology 11: 559–567.

37. Fraga RM (1989) Interactions between nectarivorous birds and the flowers of *Aphelandra sinclairiana* in Panama. Journal of Tropical Ecology 5: 19–26.

38. Franco Saldarriaga A, Restrepo Chica M (2009) Biología floral y visitantes florales de *Cavendishia nitida* (Kunth) A. C. Smith en la Reserva Natural la Montaña, Salento, Quindío. Armenia: Universidad del Quindío. 75 p.

39. Franco ALMSB (1992) Biologia floral de *Nematanthus fritschii* (Gesneriaceae). Revista Brasileira de Biologia 52: 661–666.

40. Freitas L, Sazima M (2001) Nectar features in *Esterhazya macrodonta*, a hummingbird-pollinated Scrophulariaceae in Southeastern Brazil. Journal of Plant Research 114: 187–191.

41. Freitas L, Sazima M (2006) Pollination biology in a tropical high-altitude grassland in Brazil: interactions at the community level. Annals of the Missouri Botanical Garden 93: 465–516.

42. Freitas L, Galetto L, Sazima M (2006) Pollination by hummingbirds and bees in eight syntopic species and a putative hybrid of Ericaceae in Southeastern Brazil. Plant Systematics and Evolution 258: 49–61.

43. García-Franco JG, Martinez Burgoa D, Pérez TM (2001) Hummingbird flower mites and *Tillandsia* spp. (Bromeliaceae): polyphagy in a cloud forest of Veracruz, Mexico. Biotropica 33: 538–542.

44. Gill FB (1987) Ecological fitting: use of floral nectar in *Heliconia stilesii* Daniels by three species of hermit hummingbirds. The Condor 89: 779–787.

45. Gill FB, Mack AL, Ray RT (1982) Competition between hermit hummingbirds Phaethorninae and insects for nectar un a Costa Rican Rain Forest. Ibis 124: 44–49.

46. Gómez Méndez JP, Quintana Ángel EK (2011) Comunidad de planta ornitófilas y aves asociadas en un bosque montano del Departamento de Quindío. Armenia: Universidad del Quindío. 64 p.

47. Granja Barros M, Rico-Gray V, Díaz-Castelazo C (2001) Sincronia de Floração entre *Lantana camara* L. (Verbenaceae) e *Psittacanthus calyculatus* (DC.) G. Don (Loranthaceae) ocorrentes nas dunas de la Mancha, Veracruz, México. Acta Botánica Mexicana 57: 1–14.

48. Gutierrez Z. A (2008) Las interacciones ecológicas y estructura de una comunidad altoandina de colibríes y flores en la Cordillera Oriental de Colombia. Ornitología Colombiana 07: 17–42.

49. Gutierrez Z. A, Rojas-Nossa SV, Stiles FG (2004) Dinámica anual de la interacción colibrí-flor en ecosistemas altoandinos. Neotropical Ornithology 15: 1–9.

50. Gutiérrez-Z. A, Carrillo E, Rojas S (2004) Guía ilustrada de los colibríes de la Reserva Natural Río Ñambí. Bogotá: FFA, FELCA, ECOTONO. 156 p.

51. Hernández Hernández LI (2011) Polinización de *Fouquieria formosa* Kunth por colibríes (Familia: Trochilidae) en Santa María Tecomavaca, Oaxaca. México D. F.: Universidad Nacional Autónoma de México. 50 p.

52. Hilty SL (1975) Year-round attendance of White-Whiskered and Little Hermits, *Phaethornis* sp. At singing assemblies in Colombia. Ibis 117: 382-384.

53. Hilty SL, Brown WL (1986) A Guide to the Birds of Colombia: Princeton University Press. 996 p.

54. Irwin RE (2000) Hummingbird avoidance of nectar-robbed plants: spatial location or visual cues. Oikos 91: 499–506.

55. Jacobi CM, Antonini Y (2008) Pollinators and defence of *Stachytarpheta glabra* (Verbenaceae) nectar resources by the hummingbird *Colibri serrirostris* (Trochilidae) on ironstone outcrops in south-east Brazil. Journal of Tropical Ecology 24: 301–308.

56. Jaimes Garduño MA (2008) Efecto de los ladrones de néctar (Acari: Ascidae: Mesostigmata) sobre la biología reproductiva de *Euphorbia cymbifera* (Schltdl) V.W. Steinm 2003, en Santa María Tecomavaca, Oaxaca. México, D. F.: Universidad Nacional Autónoma de México. 54 p.

57. Johnsgard PA (1997) The hummingbirds of North America. Washington, D.C.: Smithsonian Institution Press. 278 p.

58. Juiña ME, Harris JBC, Greeney HF, Hickman BR (2010) Descripción del nido y cuido parental de la Estrellita Esmeraldeña (*Chaethocercus berlepschi*) en el Occidente de Ecuador. Neotropical Ornithology 21: 313–322.

59. Kaufman K (1996) Lives of North American Birds. Boston and New York: Houghton Mifflin Harcourt. 704 p.

60. Kay E (2001) Observations on the pollination of *Passiflora penduliflora*. Biotropica 33: 709–713.

61. Lara C (2006) Temporal dynamics of flower use by hummingbirds in a highland temperate forest in Mexico. Ecosience 13: 23–29.

62. Lara C, Ornelas JF (2001) Preferential nectar robbing of flowers with long corollas: experimental studies of two hummingbird species visiting three plant species. Oecologia 128: 263–273.

63. Lara C, Ornelas JF (2001) Nectar ‘theft’ by hummingbird flower mites and its consequences for seed set in *Moussonia deppeana*. Functional Ecology 15: 78–84.

64. Lara C, Lumbreras K, González M (2009) Niche partitioning among hummingbirds foraging on *Penstemon roseus* (Plantaginaceae) in central Mexico. Neotropical Ornithology 20: 73–83.

65. Lasso E, Naranjo ME (2003) Effect of pollinators and nectar robbers on nectar production and pollen deposition in *Hamelia patens* (Rubiaceae). Biotropica 35: 57–66.

66. Leck CF (1979) Avian extinctions in an isolated tropical wet-forest preserve, Ecuador. The Auk 96: 343–352.

67. Legg K, Pitelka FA (1956) Ecologic overlap of Allen and Anna Hummingbirds nesting at Santa Cruz, California. The Condor 58: 393–405.

68. Lindberg AB, Olesen JM (2001) The fragility of extreme specialization: *Passiflora mixta* and its pollinating hummingbird *Ensifera ensifera*. Journal of Tropical Ecology 17: 323–329.

69. Linhart YB, Busby WH, Beach JH, Feinsinger P (1987) Forager behavior, pollen dispersal, and inbreeding in two species of hummingbird-pollinated plants. Evolution 41: 679–682.

70. López Saut EG (2007) Efecto de la presencia de bebederos artificiales sobre los colibríes en el Parque Ecológico de la de la Ciudad de México, Ajusco medio, México D.F.: Universidad Nacional Autónoma de México. 44 p.

71. Lyon DL (1976) A montane hummingbird territorial system in Oaxaca, Mexico. The Wilson Bulletin 88: 280–299.

72. Machado CG (2009) Beija-flores (Aves: Trochilidae) e seus recursos florais em uma área de caatinga da Chapada Diamantina, Bahia, Brasil. Zoologia 26: 255–265.

73. Magaña KC, Vázquez-Garcia JA, González Gallegos JG, Reyna Bustos O (2008) Visitantes florales en *Agave valenciana* Cházaro & A. Vázquez (Agavaceae) en Mascota, Jalisco, México. Scientia-CUCBA 10: 1–6.

74. Mendonça LB, dos Anjos L (2006) Feeding behavior of hummingbirds and perching birds on *Erythrina speciosa* Andrews (Fabaceae) flowers in an urban area, Lodrina, Panamá y Brazil. Revista Brasileira de Zoologia 23: 42–49.

75. Muchhala N (2007) Adaptive Trade-off in floral morphology mediates specialization for flowers pollinated by bats and hummingbirds. The American Naturalist 169: 494–504.

76. Nattero J, Cocucci AA (2007) Geographical variation in floral traits of the tree tobacco in relation to its hummingbird pollinator fauna. Biological Journal of the Linnean Society 90: 657–667.

77. Ornelas JF, Ordano M, Hernández A, Carlos López J, Mendoza L, et al. (2002) Nectar oasis produced by *Agave marmorata* Roezl. (Agavaceae) lead to spatial and temporal segregation among nectarivores in the Tehuacán Valley, México. Journal of Arid Environments 52: 37–51.

78. Ornelas JF, Jimenez L, Gonzalez C, Hernandez A (2004) Reproductive ecology of distylous *Palicourea padifolia* (Rubiaceae) in a tropical montane cloud forest. I. Hummingbirds' effectiveness as pollen vectors. American Journal of Botany 91: 1052–1060.

79. Ortiz-Pulido R, Diaz R (2001) Distribución de colibríes en la zona baja del centro de Veracruz, México. Neotropical Ornithology 12: 297–317.

80. Ortiz-Pulido R, Flores Ceballos E, Ortíz Pulido R (1998) Descripción del nido de *Doricha eliza* y ampliación de su rango. Neotropical Ornithology 9: 223–224.

81. Ortiz-Pulido R, Peterson AT, Robbins MB, Díaz R, Navarro-Sigüenza AG, et al. (2002) The Mexican Sheartail (*Doricha eliza*): morphology, behavior, distribution, and endangered status. The Wilson Bulletin 114: 153–160.

82. Partida Lara R (2011) Abundancia de colibríes y el uso de flores en la Reserva Ecológica Huitepec. San Cristobal de las Casas: El Colegio de la Frontera Sur. 78 p.

83. Pohl N, Carvallo G, Botto-Mahan C, Medel R (2006) Nonadditive efects of flower damage and hummingbird pollination on the fecundity of *Mimulus luteus*. Oecologia 149: 648–655.

84. Poole A, editor (2005) The Birds of North America Online. Ithaca, NY.: Cornell Laboratory of Ornithology.

85. Quesada Tyrrel E, Tyrrel RA (1990) Hummingbirds of the Caribbean. New York: Crown Publishers, Inc. 238 p.

86. Ramirez B. W (2006) Hibridación interespecífica en *Passiflora* (Passifloraceae), mediante polinización manual, y características florales para la polinización. Lankesteriana 6: 123–131.

87. Ramírez N (2007) Biología reproductiva de *Amasonia campestris* (AUBL.) Moldenke (Verbenaceae) en los llanos centrales de Venezuela. Acta Botanica Venezolana 30: 385–414.

88. Ramirez-Burbano MB, Sandoval-Sierra JV, Gomez-Bernal LG (2007) Uso de recursos florales por el Zamarrito Multicolor *Eriocnemis mirabilis* (Trochilidae) en el Parque Nacional Natural Munchique, Colombia. Ornitología Colombiana 05: 64–77.

89. Ridgely RS, Gwynne Jr. JA (1989) A guide to the birds of Panama with Costa Rica, Nicaragua, and Honduras. Londres: Princenton.

90. Rodríguez-Flores C (2002) Organización de la comunidad de colibríes ermitaños (Trochilidae: Phaethorninae) y sus flores en bosques de tierra firme del Parque Nacional Natural Amacayacu (Amazonas, Colombia). Bogotá: Universidad Nacional de Colombia. 125 p.

91. Rodríguez-Flores C (2009) Dinámica de las estrategias de forrajeo por néctar en colibríes (Aves: Trochilidae) en la Reserva de la Biosfera Sierra de Manantlán (Jalisco, México). Ciudad de México: Universidad Nacional Autónoma de México. 75 p.

92. Rosero LL, Sazima M (2004) Interacciones planta-colibrí en tres comunidades vegetales de la parte Suroriental del Parque Nacional Natural Chiribiquete, Colombia". Neotropical Ornithology 15: 183–190.

93. Ruschi A (1961) Algumas observações sobre: *Phaethornis yaruqui yarqui* (Bourcier), *Boissonneaua jardinii* (Bourcier), *Doryfera ludoviciae ludoviciae* (Bourcier & Mulsant), *Heliangelus viola* (Gould), *Colibri coruscans coruscans* (Gould)*, Helianthea torquata fulgidugula* (Gould), *Aglaiocercus kingii mocoa* (DeLattre & Bourcier) e *Aglaiocercus kingii margarethae* (Heine). Boletim do Museu de Biologia Mello Leitão 27: 1-21.

94. Sazima I, Buzato S, Sazima M (1995) The Saw-billed Hermit *Ramphodon naevius* and its flowers in southeastern Brazil. Journal of Ornithology 136: 195–206.

95. Sazima M, Vogel S, do Prado AL, de Oliveira DM, Franz G, et al. (2001) The sweet jelly of *Combretum lanceolatum* flowers (Combretaceae): a cornucopia resource for bird pollinators in the Pantanal, western Brazil. Plant Systematics and Evolution 227: 195–208.

96. Schuchmann KL (1999) Family Trochilidae (Hummingbirds). In: del Hoyo J, Elliott A, Sargatal J, editors. Handbook of the birds of the world. Barcelona: Lynx Edicions. pp. 468–680.

97. Scobell SA, Scott PE (2002) Visitors and floral traits of a hummingbird-adapted cactus (*Echinocereus coccineus*) show only minor variation along an elevational gradient. The American Midland Naturalist 147: 1–15.

98. Singer RB (2003) Orchid Pollination: Recent developments from Brazil. Lankesteriana 07: 111–114.

99. Singer RB, Sazima M (2000) The pollination of *Stenorrhynchos lanceolatus* (Aublet) L. C. Rich. (Orchidaceae: Spiranthinae) by hummingbirds in southeastern Brazil. Plant Systematics and Evolution 223: 221–227.

100. Skutch AF (1964) Life histories of hermit hummingbirds. The Auk 81: 5–25.

101. Smith-Ramirez C, Armesto JJ (2003) Foraging behaviour of bird pollinators on *Embothrium coccineum* (Proteaceae) trees in forest fragments and pastures in southern Chile. Austral Ecology 28: 53–60.

102. Snow BK (1973) Social organization of the Hairy Hermit, *Glaucis hirsuta*. Ardea 61: 94–105.

103. Snow BK (1973) The behavior and ecology of hermit hummingbirds in the Kanaku Mountains, Guyana. The Wilson Bulletin 85: 163–177.

104. Snow BK (1974) Lek behaviour and breeding of Guy's Hermit Hummingbird *Phaethornis guy*. Ibis 116: 278–297.

105. Snow BK (1977) Feeding behavior of two hummingbirds in a Costa Rican montane forest. The Wilson Bulletin 89: 613–616.

106. Snow BK (1981) Relationships between hermit hummingbirds and their food plants in eastern Ecuador. Bulletin of the British Ornithologists' Club 101: 387–396.

107. Snow BK, Snow DW (1972) Feeding niches of hummingbirds in a Trinidad Valley. Journal of Animal Ecology 41: 471–485.

108. Snow DW, Snow BK (1980) Relationships between hummingbirds and flowers in the Andes of Colombia. Bulletin of the British Museum (Natural History) Zoology 38: 105–139.

109. Stein BA (1992) Sicklebill hummingbirds, ants, and flowers. Bioscience 42: 27–33.

110. Stiles FG (1975) Ecology, flowering phenology, and hummingbird pollination of some Costa Rican *Heliconia* species. Ecology 56: 285–301.

111. Stiles FG (1979) Notes on the natural history of *Heliconia* (Musaceae) in Costa Rica. Brenesia 15: 151–180.

112. Stiles FG (1980) The annual cycle in a tropical wet forest hummingbird community. Ibis 122: 322–343.

113. Stiles FG (1981) Geographical aspects of bird-flower coevolution, with particular reference to Central America. Annals of the Missouri Botanical Garden 68: 323–351.

114. Stiles FG (1985) Seasonal patterns and coevolution in the hummingbird-flower community of a Costa Rican subtropical forest. Ornithological Monographs 36: 757-787.

115. Stiles FG (1995) Behavioral, ecological and morphological correlates of foraging for arthropods by the hummingbirds of a tropical west forest. The Condor 97: 853–878.

116. Stiles FG, Skutch AF (1987) A Guide to the Birds of Costa Rica London: Christopher Helm.

117. Streisfeld MA, Konh JR (2006) Environment and pollinator-mediated selection on parapatric floral races of *Mimulus aurantiacus*. Journal of Evolutionary Biology 20: 122–132.

118. Temeles E, Shaw K, Kudla A, Sander S (2006) Traplining by purple-throated carib hummingbirds: behavioral responses to competition and nectar availability. Behavioral Ecology and Sociobiology 61: 163–172.

119. Temeles EJ, Koulouris CR, Sander SE, Kress WJ (2009) Effect of flower shape and size on foraging performance and trade-offs in a tropical hummingbird. Ecology 90: 1147–1161.

120. Temeles EJ, Kress WJ (2003) Adaptation in a Plant-Hummingbird Association. Science 300: 630-633.

121. Valenzuela Galván D, Mariano NA, Osorio Berinstain M, Urbina F (2006) First record of the sparkling-tailed humminbird *Tilmatura dupontii*, Trochilidae for Sierra de Huautla Biosphere Reserve, Morelos, México. Huitzil Revista de Ornitología Mexicana 7: 15–17.

122. Van Devender TR, Calder WA, Krebbs K, Reina AL, Russell SM, et al. (2004) Hummingbird plants and potential nectar corridors for the Rufous hummingbird in Sonora, Mexico. In: Nabhan GP, editor. Conserving Migratory Pollinators and Nectar Corridors in Western North America. Tucson, Arizona: University of Arizona Press. pp. 96-121.

123. Varassin IG, Trigo JR, Sazima M (2001) The role of nectar production, flower pigments and odour in the pollination of four species of *Passiflora* (Passifloraceae) in south-eastern Brazil. Botanical Journal of the Linnean Society 136: 139–152.

124. Wester P, Claßen-Bockhoff R (2006) Hummingbird pollination in *Salvia haenkei* (Lamiaceae) lacking the typical lever mechanism. Plant Systematics and Evolution 257: 133–146.
