# Supplementary material for: Are hummingbirds generalists or specialists? Using network analysis to explore the mechanisms influencing their interaction with nectar resources
Source: PLoS One. 2019 Feb 27;14(2):e0211855. doi: 10.1371/journal.pone.0211855 (PMC6392410; doi:10.1371/journal.pone.0211855)
Supplement: S2 Table — Hummingbird clades are in columns and plant families in rows; native and non-native plant species were included. In this binary matrix, 1 indicates an interaction between a hummingbird clade and a plant family, and 0 otherwise. Nodes are ordered by number of links. (DOCX) [file pone.0211855.s004.docx]

**S2 Table. Binary matrix of the interaction network between hummingbird clades and their nectar family plants.** Hummingbird clades are in columns and plant families in rows; native and non-native plant species were included. In this binary matrix, 1 indicates an interaction between a hummingbird clade and a plant family, and 0 otherwise. Nodes are ordered by number of links.

**S2 Table**

|  | Emeralds | Bees | Coquettes | MtGems | Mangoes | Hermits | Brilliants | Topazes | Patagona |
| --- | --- | --- | --- | --- | --- | --- | --- | --- | --- |
| Asteraceae | 1 | 1 | 1 | 1 | 1 | 1 | 1 | 1 | 1 |
| Bromeliaceae | 1 | 1 | 1 | 1 | 1 | 1 | 1 | 1 | 1 |
| Myrtaceae | 1 | 1 | 1 | 1 | 1 | 1 | 1 | 1 | 1 |
| Lamiaceae | 1 | 1 | 1 | 1 | 1 | 1 | 1 | 0 | 1 |
| Ericaceae | 1 | 1 | 1 | 1 | 1 | 1 | 1 | 1 | 0 |
| Campanulaceae | 1 | 1 | 1 | 1 | 1 | 1 | 1 | 0 | 1 |
| Fabaceae | 1 | 1 | 1 | 1 | 1 | 1 | 1 | 1 | 0 |
| Gesneriaceae | 1 | 1 | 1 | 1 | 1 | 1 | 1 | 1 | 0 |
| Loranthaceae | 1 | 1 | 1 | 1 | 1 | 1 | 1 | 0 | 1 |
| Solanaceae | 1 | 1 | 1 | 1 | 1 | 1 | 1 | 0 | 1 |
| Heliconiaceae | 1 | 1 | 1 | 1 | 1 | 1 | 1 | 1 | 0 |
| Malvaceae | 1 | 1 | 1 | 1 | 1 | 1 | 1 | 1 | 0 |
| Marcgraviaceae | 1 | 1 | 1 | 1 | 1 | 1 | 1 | 1 | 0 |
| Rubiaceae | 1 | 1 | 1 | 1 | 1 | 1 | 1 | 1 | 0 |
| Acanthaceae | 1 | 1 | 1 | 1 | 1 | 1 | 1 | 0 | 0 |
| Apocynaceae | 1 | 1 | 1 | 1 | 1 | 1 | 0 | 1 | 0 |
| Alstroemeriaceae | 1 | 1 | 1 | 1 | 1 | 1 | 1 | 0 | 0 |
| Passifloraceae | 1 | 1 | 0 | 1 | 1 | 1 | 1 | 0 | 1 |
| Bignoniaceae | 1 | 1 | 1 | 1 | 1 | 1 | 0 | 1 | 0 |
| Lythraceae | 1 | 1 | 1 | 1 | 1 | 1 | 1 | 0 | 0 |
| Onagraceae | 1 | 1 | 1 | 1 | 1 | 1 | 1 | 0 | 0 |
| Marantaceae | 1 | 1 | 1 | 0 | 1 | 1 | 1 | 0 | 0 |
| Boraginaceae | 1 | 1 | 1 | 1 | 1 | 1 | 0 | 0 | 0 |
| Euphorbiaceae | 1 | 1 | 1 | 1 | 1 | 0 | 0 | 1 | 0 |
| Clusiaceae | 1 | 0 | 1 | 1 | 1 | 0 | 1 | 1 | 0 |
| Vochysiaceae | 1 | 1 | 1 | 1 | 1 | 0 | 0 | 1 | 0 |
| Verbenaceae | 1 | 1 | 1 | 1 | 1 | 1 | 0 | 0 | 0 |
| Orobanchaceae | 1 | 1 | 1 | 1 | 1 | 0 | 1 | 0 | 0 |
| Costaceae | 1 | 1 | 0 | 1 | 0 | 1 | 1 | 1 | 0 |
| Rutaceae | 1 | 1 | 1 | 0 | 1 | 1 | 1 | 0 | 0 |
| Musaceae | 1 | 1 | 0 | 1 | 1 | 1 | 1 | 0 | 0 |
| Orchidaceae | 1 | 0 | 1 | 1 | 1 | 1 | 1 | 0 | 0 |
| Scrophulariaceae | 1 | 1 | 1 | 1 | 1 | 0 | 0 | 0 | 1 |
| Cactaceae | 1 | 1 | 0 | 1 | 1 | 0 | 0 | 0 | 1 |
| Zingiberaceae | 1 | 0 | 0 | 1 | 1 | 1 | 1 | 0 | 0 |
| Gentianaceae | 1 | 1 | 1 | 0 | 0 | 1 | 1 | 0 | 0 |
| Grossulariaceae | 1 | 1 | 1 | 1 | 1 | 0 | 0 | 0 | 0 |
| Rosaceae | 1 | 1 | 1 | 0 | 0 | 1 | 1 | 0 | 0 |
| Asparagaceae | 1 | 1 | 1 | 1 | 0 | 0 | 0 | 0 | 1 |
| Melastomataceae | 1 | 1 | 1 | 0 | 1 | 0 | 1 | 0 | 0 |
| Combretaceae | 1 | 1 | 0 | 0 | 1 | 1 | 0 | 0 | 0 |
| Convolvulaceae | 1 | 1 | 0 | 1 | 1 | 0 | 0 | 0 | 0 |

**S2 Table** (continued)

|  | Emeralds | Bees | Coquettes | MtGems | Mangoes | Hermits | Brilliants | Topazes | Patagona |
| --- | --- | --- | --- | --- | --- | --- | --- | --- | --- |
| Cucurbitaceae | 1 | 0 | 0 | 1 | 1 | 1 | 0 | 0 | 0 |
| Crassulaceae | 1 | 1 | 1 | 0 | 1 | 0 | 0 | 0 | 0 |
| Proteaceae | 1 | 1 | 1 | 0 | 0 | 0 | 1 | 0 | 0 |
| Plantaginaceae | 1 | 1 | 0 | 1 | 1 | 0 | 0 | 0 | 0 |
| Amaryllidaceae | 1 | 1 | 1 | 0 | 0 | 1 | 0 | 0 | 0 |
| Salicaceae | 1 | 1 | 0 | 0 | 1 | 1 | 0 | 0 | 0 |
| Tropaeolaceae | 0 | 1 | 0 | 1 | 0 | 0 | 1 | 0 | 0 |
| Berberidaceae | 0 | 1 | 1 | 0 | 0 | 0 | 1 | 0 | 0 |
| Balsaminaceae | 1 | 1 | 0 | 0 | 0 | 1 | 0 | 0 | 0 |
| Loasaceae | 0 | 1 | 1 | 0 | 0 | 0 | 1 | 0 | 0 |
| Urticaceae | 1 | 0 | 1 | 0 | 1 | 0 | 0 | 0 | 0 |
| Caprifoliaceae | 1 | 1 | 0 | 1 | 0 | 0 | 0 | 0 | 0 |
| Anacardiaceae | 1 | 1 | 1 | 0 | 0 | 0 | 0 | 0 | 0 |
| Cannaceae | 1 | 1 | 0 | 0 | 0 | 1 | 0 | 0 | 0 |
| Iridaceae | 1 | 1 | 0 | 1 | 0 | 0 | 0 | 0 | 0 |
| Phrymaceae | 0 | 1 | 1 | 1 | 0 | 0 | 0 | 0 | 0 |
| Meliaceae | 1 | 0 | 1 | 0 | 0 | 1 | 0 | 0 | 0 |
| Clethraceae | 1 | 1 | 1 | 0 | 0 | 0 | 0 | 0 | 0 |
| Polemoniaceae | 1 | 1 | 0 | 1 | 0 | 0 | 0 | 0 | 0 |
| Sapindaceae | 1 | 1 | 0 | 0 | 0 | 1 | 0 | 0 | 0 |
| Caricaceae | 1 | 0 | 0 | 0 | 0 | 1 | 0 | 0 | 0 |
| Liliaceae | 0 | 1 | 0 | 1 | 0 | 0 | 0 | 0 | 0 |
| Ranunculaceae | 0 | 1 | 0 | 1 | 0 | 0 | 0 | 0 | 0 |
| Fouquieriaceae | 1 | 1 | 0 | 0 | 0 | 0 | 0 | 0 | 0 |
| Polygonaceae | 1 | 0 | 0 | 0 | 0 | 1 | 0 | 0 | 0 |
| Lecythidaceae | 1 | 0 | 0 | 0 | 0 | 1 | 0 | 0 | 0 |
| Nyctaginaceae | 1 | 1 | 0 | 0 | 0 | 0 | 0 | 0 | 0 |
| Simaroubaceae | 1 | 0 | 0 | 0 | 1 | 0 | 0 | 0 | 0 |
| Pittosporaceae | 1 | 1 | 0 | 0 | 0 | 0 | 0 | 0 | 0 |
| Hypericaceae | 1 | 0 | 0 | 0 | 0 | 0 | 1 | 0 | 0 |
| Plumbaginaceae | 1 | 1 | 0 | 0 | 0 | 0 | 0 | 0 | 0 |
| Sapotaceae | 1 | 1 | 0 | 0 | 0 | 0 | 0 | 0 | 0 |
| Geraniaceae | 0 | 0 | 1 | 1 | 0 | 0 | 0 | 0 | 0 |
| Strelitziaceae | 1 | 1 | 0 | 0 | 0 | 0 | 0 | 0 | 0 |
| Xanthorrhoeaceae | 1 | 1 | 0 | 0 | 0 | 0 | 0 | 0 | 0 |
| Caryophyllaceae | 0 | 1 | 0 | 1 | 0 | 0 | 0 | 0 | 0 |
| Brassicaceae | 0 | 1 | 1 | 0 | 0 | 0 | 0 | 0 | 0 |
| Saxifragaceae | 0 | 1 | 0 | 1 | 0 | 0 | 0 | 0 | 0 |
| Velloziaceae | 1 | 0 | 0 | 0 | 1 | 0 | 0 | 0 | 0 |
| Betulaceae | 0 | 1 | 0 | 0 | 0 | 0 | 0 | 0 | 0 |
| Columelliaceae | 0 | 0 | 0 | 0 | 0 | 0 | 1 | 0 | 0 |

**S2 Table** (continued)

|  | Emeralds | Bees | Coquettes | MtGems | Mangoes | Hermits | Brilliants | Topazes | Patagona |
| --- | --- | --- | --- | --- | --- | --- | --- | --- | --- |
| Elaeocarpaceae | 0 | 0 | 1 | 0 | 0 | 0 | 0 | 0 | 0 |
| Araliaceae | 1 | 0 | 0 | 0 | 0 | 0 | 0 | 0 | 0 |
| Cleomaceae | 0 | 1 | 0 | 0 | 0 | 0 | 0 | 0 | 0 |
| Begoniaceae | 1 | 0 | 0 | 0 | 0 | 0 | 0 | 0 | 0 |
| Muntingiaceae | 0 | 1 | 0 | 0 | 0 | 0 | 0 | 0 | 0 |
| Bonnetiaceae | 1 | 0 | 0 | 0 | 0 | 0 | 0 | 0 | 0 |
| Calycanthaceae | 1 | 0 | 0 | 0 | 0 | 0 | 0 | 0 | 0 |
| Calophyllaceae | 0 | 1 | 0 | 0 | 0 | 0 | 0 | 0 | 0 |
| Loganiaceae | 1 | 0 | 0 | 0 | 0 | 0 | 0 | 0 | 0 |
| Moraceae | 1 | 0 | 0 | 0 | 0 | 0 | 0 | 0 | 0 |
| Symplocaceae | 0 | 0 | 1 | 0 | 0 | 0 | 0 | 0 | 0 |
| Oleaceae | 0 | 1 | 0 | 0 | 0 | 0 | 0 | 0 | 0 |
| Papaveraceae | 0 | 1 | 0 | 0 | 0 | 0 | 0 | 0 | 0 |
| Tetrameristaceae | 1 | 0 | 0 | 0 | 0 | 0 | 0 | 0 | 0 |
| Ochnaceae | 0 | 0 | 0 | 0 | 0 | 0 | 1 | 0 | 0 |
| Vitaceae | 1 | 0 | 0 | 0 | 0 | 0 | 0 | 0 | 0 |
| Rhamnaceae | 0 | 1 | 0 | 0 | 0 | 0 | 0 | 0 | 0 |
| Escalloniaceae | 0 | 0 | 1 | 0 | 0 | 0 | 0 | 0 | 0 |
| Phyllanthaceae | 0 | 0 | 1 | 0 | 0 | 0 | 0 | 0 | 0 |
| Polygalaceae | 0 | 1 | 0 | 0 | 0 | 0 | 0 | 0 | 0 |
| Zygophyllaceae | 0 | 1 | 0 | 0 | 0 | 0 | 0 | 0 | 0 |
| Chrysobalanaceae | 1 | 0 | 0 | 0 | 0 | 0 | 0 | 0 | 0 |
