# Supplementary material for: Are hummingbirds generalists or specialists? Using network analysis to explore the mechanisms influencing their interaction with nectar resources
Source: PLoS One. 2019 Feb 27;14(2):e0211855. doi: 10.1371/journal.pone.0211855 (PMC6392410; doi:10.1371/journal.pone.0211855)
Supplement: S3 Table — Plant families associated with hummingbird clades in more than 90% of the modularity analyses. We used the network with nodes ordered phylogenetically and only included native plant species. Numbers in parenthesis correspond to the number of analyses in which the plant family belonged to the same module as the hummingbird clade. The text color represents the pollination syndrome of each family: red (ornithophilous), blue (intermediate), and black (non-ornithophilous). See Table 2 in the text for details on the characteristics of each pollination syndrome category. (DOCX) [file pone.0211855.s005.docx]

**S3 Table. Plant families and hummingbird clades associated by modularity analysis.** Plant families associated with hummingbird clades in more than 90% of the modularity analyses. We used the network with nodes ordered phylogenetically and only included native plant species. Numbers in parenthesis correspond to the number of analyses in which the plant family belonged to the same module as the hummingbird clade. The text color represents the pollination syndrome of each family: red (ornithophilous), blue (intermediate), and black (non-ornithophilous). See Table 2 in the text for details on the characteristics of each pollination syndrome category.

.

| Bees | Mountain Gems | Emeralds | Patagona | Coquettes | Brilliants | Mangoes | Hermits | Topazes |
| --- | --- | --- | --- | --- | --- | --- | --- | --- |
| Betulaceae (50) | Asparagaceae (45) | Araliaceae (50) | Campanulaceae (50) | Berberidaceae (47) | Columelliaceae (50) |  |  | Ericaceae (50) |
| Caprifoliaceae (48) | Cactaceae (45) | Begoniaceae (50) | Lamiaceae (50) | Elaeocarpaceae (50) | Ochnaceae (50) |  |  | Fabaceae (50) |
| Caryophyllaceae (50) | Convolvulaceae (46) | Bonnetiaceae (50) | Loranthaceae (50) | Escalloniaceae (50) |  |  |  | Gesneriaceae (50) |
| Cleomaceae (50) | Grossulariaceae (46) | Calycanthaceae (50) | Passifloraceae (45) | Loasaceae (47) |  |  |  | Heliconiaceae (50) |
| Iridaceae (46) | Plantaginaceae (46) | Caricaceae (48) | Scrophulariaceae (45) | Melastomataceae (50) |  |  |  | Malvaceae (50) |
| Liliaceae (50) |  | Chrysobalanaceae (50) | Solanaceae (50) | Phyllanthaceae (50) |  |  |  | Marcgraviaceae (50) |
| Muntingiaceae (50) |  | Lecythidaceae (48) |  | Proteaceae (47) |  |  |  | Rubiaceae (50) |
| Papaveraceae (50) |  | Loganiaceae (50) |  | Symplocaceae (50) |  |  |  |  |
| Phrymaceae (49) |  | Moraceae (50) |  |  |  |  |  |  |
| Plumbaginaceae (50) |  | Polygonaceae (50) |  |  |  |  |  |  |
| Polemoniaceae (47) |  | Strelitziaceae (50) |  |  |  |  |  |  |
| Polygalaceae (50) |  | Tetrameristaceae (50) |  |  |  |  |  |  |
| Ranunculaceae (50) |  |  |  |  |  |  |  |  |
| Rhamnaceae (50) |  |  |  |  |  |  |  |  |
| Saxifragaceae (50) |  |  |  |  |  |  |  |  |
| Tropaeolaceae (48) |  |  |  |  |  |  |  |  |
| Zygophyllaceae (50) |  |  |  |  |  |  |  |  |
