# Supplementary material for: Are hummingbirds generalists or specialists? Using network analysis to explore the mechanisms influencing their interaction with nectar resources
Source: PLoS One. 2019 Feb 27;14(2):e0211855. doi: 10.1371/journal.pone.0211855 (PMC6392410; doi:10.1371/journal.pone.0211855)
Supplement: S4 Table — The source(s) of information for each category is (are) shown in the REF column and the Supplementary References section (see below). For the biogeographical distribution regions, the value 1 represents the presence of a hummingbird species in this (these) region(s); in contrast, 0 represents the lack of records. The latitudinal and elevational range columns are the difference between the minimum and maximum value for each category. The asterisk (*) in the Geographical Area column means that the center of diversification of these hummingbird species was indirectly inferred based on the closest sister species whose center of diversification has been explicitly detected. Also, the inference method used in the different studies to establish the center of diversification area is shown. For the definition of each biogeographical region and geographical area, see the text. For exposed culmen, weight, and wing morphological information, the mean and standard deviation (s.d.) of each hummingbird species are shown. The definition of each bill curvature category is explained in the text. (DOCX) [file pone.0211855.s006.docx]

**S4 Table. Biogeographical, elevational, latitudinal, and morphological information and center of diversification for the hummingbird species included in this study.** The source(s) of information for each category is (are) shown in the REF column and the Supplementary References section (see below). For the biogeographical distribution regions, the value 1 represents the presence of a hummingbird species in this (these) region(s); in contrast, 0 represents the lack of records. The latitudinal and elevational range columns are the difference between the minimum and maximum value for each category. The asterisk (*) in the Geographical Area column means that the center of diversification of these hummingbird species was indirectly inferred based on the closest sister species whose center of diversification has been explicitly detected. Also, the inference method used in the different studies to establish the center of diversification area is shown. For the definition of each biogeographical region and geographical area, see the text. For exposed culmen, weight, and wing morphological information, the mean and standard deviation (s.d.) of each hummingbird species are shown. The definition of each bill curvature category is explained in the text.

|  |  | Biogeographical Region | | | | | Latitude Distribution (°) | | | | Elevation Distribution (m.a.s.l.) | | | | Center of Diversification | | | Culmen (mm) | | Weight (g) | | Wing (mm) | | Curvature | Morphology |
| --- | --- | --- | --- | --- | --- | --- | --- | --- | --- | --- | --- | --- | --- | --- | --- | --- | --- | --- | --- | --- | --- | --- | --- | --- | --- |
| Clade | Species | Neartic | Neotropical | Caribbean | Austral | REF | High | Low | Range | REF | Minimum | Maximum | Range | REF | Geographical Area* | Inference | REF | Mean | s.d. | Mean | s.d. | Mean | s.d. | Mean | REF |
| Bees | *Archilochus alexandri* | 1 | 1 | 0 | 0 | [1, 2] | 50 | 32 | 18 | [3] | 0 | 2000 | 2000 | [4] | North America | Ancestral State Reconstruction | [5] | 17.673 | 1.254 | 2.950 | 0.286 | 41.145 | 2.292 | 2 | [6], [3], Núñez L. (unpublished data), Rodríguez-Flores (unpublished data) |
| Bees | *Archilochus colubris* | 1 | 1 | 0 | 0 | [1, 2] | 60 | 30 | 30 | [3] | 0 | 2450 | 2450 | [7] | North America | Ancestral State Reconstruction | [5] | 17.763 | 1.384 | 2.977 | 0.493 | 43.269 | 2.249 | 2 | [6], [3], Núñez L. (unpublished data) |
| Bees | *Atthis ellioti* | 0 | 1 | 0 | 0 | [1, 2] | 14 | 14 | 0.2 | [3] | 900 | 3300 | 2400 | [7] | North America | Ancestral State Reconstruction | [5] |  |  | 2.300 | 0.424 |  |  | 2 | [6] |
| Bees | *Atthis heloisa* | 1 | 1 | 0 | 0 | [1, 2] | 26 | 17 | 9 | [3] | 1500 | 2900 | 1400 | [7] | North America | Ancestral State Reconstruction | [5] | 13.033 | 0.357 | 2.132 | 0.192 | 36.458 | 3.035 | 2 | [6], [3], Núñez L. (unpublished data) |
| Bees | *Calliphlox amethystina* | 0 | 1 | 0 | 0 | [1, 2] | 6 | -28 | 34 | [3] | 0 | 1500 | 1500 | [7] | South America | Ancestral State Reconstruction | [5] |  |  | 2.400 | 0.141 |  |  | 2 | [6] |
| Bees | *Calliphlox bryantae* | 0 | 1 | 0 | 0 | [1, 2] | 10 | 9 | 1 | [3] | 1830 | 1830 | 0 | [7] | South America | Ancestral State Reconstruction | [5] | 19.900 | 0.300 | 3.343 | 0.067 | 41.700 | 0.300 | 2 | [6], Núñez L. (unpublished data) |
| Bees | *Calliphlox evelynae* | 0 | 0 | 1 | 0 | [1, 2] | 27 | 21 | 6 | [3] | 0 | 100 | 100 | [2] | Caribbean | Ancestral State Reconstruction | [5] | 15.750 | 0.350 | 2.850 | 0.370 | 40.050 | 0.750 | 3 | [6], Núñez L. (unpublished data) |
| Bees | *Calliphlox mitchellii* | 0 | 1 | 0 | 0 | [1, 2] | 9 | 0 | 9 | [3] | 0 | 1900 | 1900 | [7] | South America | Ancestral State Reconstruction | [5] |  |  | 3.150 | 0.212 |  |  | 2 | [6] |
| Bees | *Calothorax lucifer* | 1 | 1 | 0 | 0 | [1, 2] | 33 | 19 | 14 | [3] | 1100 | 2250 | 1150 | [7] | North America | Ancestral State Reconstruction | [5] | 12.350 | 0.050 | 3.275 | 0.287 | 36.350 | 1.350 | 3 | [6], Núñez L. (unpublished data) |
| Bees | *Calothorax pulcher* | 1 | 1 | 0 | 0 | [1, 2] | 19 | 16 | 3 | [3] | 1000 | 2000 | 1000 | [7] | North America | Ancestral State Reconstruction | [5] |  |  | 2.850 | 0.354 |  |  | 3 | [6] |
| Bees | *Calypte anna* | 1 | 0 | 0 | 0 | [1, 2] | 45 | 30 | 15 | [3] | 0 | 1800 | 1800 | [7] | North America | Ancestral State Reconstruction | [5] | 18.500 | 0.300 | 4.275 | 0.955 | 49.650 | 0.050 | 2 | [6], Núñez L. (unpublished data) |
| Bees | *Calypte costae* | 1 | 1 | 0 | 0 | [1, 2] | 38 | 29 | 9 | [3] | 0 | 1500 | 1500 | [7] | North America | Ancestral State Reconstruction | [5] | 18.160 | 0.755 | 2.783 | 0.361 | 42.984 | 2.372 | 2 | [6], [3], Núñez L. (unpublished data) |
| Bees | *Chaetocercus astreans* | 0 | 1 | 0 | 0 | [1, 2] | 11 | 10 | 1 | [3] | 825 | 2000 | 1175 | [2] | South America* | Ancestral State Reconstruction | [8] |  |  |  |  |  |  | 2 | [6] |
| Bees | *Chaetocercus berlepschi* | 0 | 1 | 0 | 0 | [1, 2] | 2 | -5 | 7 | [3] | 0 | 500 | 500 | [2] | South America* | Ancestral State Reconstruction | [8] |  |  |  |  |  |  | 2 | [6] |
| Bees | *Chaetocercus bombus* | 0 | 1 | 0 | 0 | [1, 2] | -1 | -12 | 11 | [3] | 0 | 3000 | 3000 | [2] | South America | Ancestral State Reconstruction | [5] |  |  |  |  |  |  | 2 | [6] |
| Bees | *Chaetocercus heliodor* | 0 | 1 | 0 | 0 | [1, 2] | 10 | 0 | 10 | [3] | 500 | 3000 | 2500 | [7] | South America* | Ancestral State Reconstruction | [8] |  |  |  |  |  |  | 2 | [6] |
| Bees | *Chaetocercus jourdanii* | 0 | 1 | 0 | 0 | [1, 2] | 11 | 7 | 3 | [3] | 900 | 3000 | 2100 | [7] | South America* | Ancestral State Reconstruction | [8] |  |  |  |  |  |  | 2 | [6] |
| Bees | *Chaetocercus mulsant* | 0 | 1 | 0 | 0 | [1, 2] | 4 | -18 | 22 | [3] | 1500 | 2800 | 1300 | [7] | South America | Ancestral State Reconstruction | [5] |  |  | 3.800 |  |  |  | 2 | [6] |
| Bees | *Doricha eliza* | 0 | 1 | 0 | 0 | [1, 2] | 19 | 18 | 1 | [3] | 300 | 2250 | 1950 | [7] | North America | Ancestral State Reconstruction | [5] | 21.850 | 0.550 | 2.475 | 0.150 | 37.900 | 0.800 | 4 | [6] |
| Bees | *Eulidia yarrellii* | 0 | 0 | 0 | 1 | [1, 2] | -17 | -24 | 7 | [3] | 200 | 1840 | 1640 | [2] | South America | Ancestral State Reconstruction | [5] |  |  | 2.450 | 0.212 |  |  | 2 | [6] |
| Bees | *Mellisuga helenae* | 0 | 0 | 1 | 0 | [1, 2] | 23 | 19 | 4 | [3] | 0 | 500 | 500 | [7] | Caribbean* | Ancestral State Reconstruction | [5] |  |  | 1.750 | 0.212 |  |  | 2 | [6] |
| Bees | *Mellisuga minima* | 0 | 0 | 1 | 0 | [1, 2] | 20 | 18 | 2 | [3] | 0 | 1600 | 1600 | [2] | Caribbean | Ancestral State Reconstruction | [5] |  |  | 3.000 | 0.424 |  |  | 2 | [6], Núñez L. (unpublished data) |
| Bees | *Myrmia micrura* | 0 | 1 | 0 | 0 | [1, 2] | -1 | -9 | 8 | [3] | 0 | 100 | 100 | [7] | South America | Ancestral State Reconstruction | [5] |  |  | 3.000 | 0.424 |  |  | 3 | [6] |
| Bees | *Myrtis fanny* | 0 | 1 | 0 | 0 | [1, 2] | -1 | -12 | 11 | [3] | 1200 | 2800 | 1600 | [7] | South America | Ancestral State Reconstruction | [5] |  |  | 2.375 | 0.087 |  |  | 2 | [6], Núñez L. (unpublished data) |
| Bees | *Rhodopis vesper* | 0 | 1 | 0 | 1 | [1, 2] | -6 | -28 | 22 | [3] | 0 | 3050 | 3050 | [7] | South America | Ancestral State Reconstruction | [5] |  |  | 3.900 | 0.424 |  |  | 3 | [6] |
| Bees | *Selasphorus calliope* | 1 | 1 | 0 | 0 | [1, 2] | 50 | 31 | 19 | [3] | 180 | 3500 | 3320 | [7] | North America | Ancestral State Reconstruction | [5] | 14.506 | 0.953 | 2.270 | 0.386 | 39.906 | 2.044 | 2 | [6], [3], Núñez L. (unpublished data) |
| Bees | *Selasphorus flammula* | 0 | 1 | 0 | 0 | [1, 2] | 11 | 8 | 3 | [3] | 1700 | 3100 | 1400 | [7] | North America | Ancestral State Reconstruction | [5] | 12.325 | 0.575 | 2.650 | 0.173 | 40.995 | 0.955 | 2 | [6], Núñez L. (unpublished data) |
| Bees | *Selasphorus platycercus* | 1 | 1 | 0 | 0 | [1, 2] | 45 | 15 | 30 | [3] | 900 | 3350 | 2450 | [7] | North America | Ancestral State Reconstruction | [5] | 18.233 | 1.503 | 2.909 | 0.431 | 45.828 | 4.038 | 2 | [6], [3], Núñez L. (unpublished data) |
| Bees | *Selasphorus rufus* | 1 | 1 | 0 | 0 | [1, 2] | 60 | 42 | 18 | [3] | 0 | 2250 | 2250 | [7] | North America | Ancestral State Reconstruction | [5] | 17.327 | 1.450 | 2.948 | 0.440 | 42.452 | 2.022 | 2 | [6], [3], Núñez L. (unpublished data) |
| Bees | *Selasphorus sasin* | 1 | 0 | 0 | 0 | [1, 2] | 45 | 35 | 10 | [3] | 300 | 2500 | 2200 | [7] | North America | Ancestral State Reconstruction | [5] | 16.233 | 1.167 | 3.134 | 0.468 | 38.800 | 2.007 | 2 | [6], [3], Núñez L. (unpublished data) |
| Bees | *Selasphorus scintilla* | 0 | 1 | 0 | 0 | [1, 2] | 9 | 8 | 1 | [3] | 1220 | 3000 | 1780 | [7] | North America | Ancestral State Reconstruction | [5] | 11.350 | 0.600 | 2.213 | 0.119 | 34.265 | 1.555 | 2 | [6], Núñez L. (unpublished data) |
| Bees | *Thaumastura cora* | 0 | 1 | 0 | 1 | [1, 2] | -6 | -20 | 14 | [3] | 0 | 2400 | 2400 | [2] | South America | Ancestral State Reconstruction | [5] |  |  | 2.250 | 0.354 |  |  | 2 | [6] |
| Bees | *Tilmatura dupontii* | 1 | 1 | 0 | 0 | [1, 2] | 24 | 13 | 11 | [3] | 500 | 1950 | 1450 | [7] | North America | Ancestral State Reconstruction | [5] | 14.700 | 1.200 | 2.650 | 0.472 | 34.650 | 0.950 | 2 | [6], [3] |
| Brilliants | *Aglaeactis aliciae* | 0 | 1 | 0 | 0 | [1, 2] | -6 | -8 | 2 | [3] | 3000 | 3500 | 500 | [2] | South America* | Ancestral State Reconstruction | [8] |  |  | 7.800 | 0.707 |  |  | 2 | [6] |
| Brilliants | *Aglaeactis castelnaudii* | 0 | 1 | 0 | 0 | [1, 2] | -10 | -14 | 4 | [3] | 3500 | 4300 | 800 | [2] | South America | Ancestral State Reconstruction | [8] |  |  | 7.750 | 0.612 |  |  | 2 | [6], Núñez L. (unpublished data) |
| Brilliants | *Aglaeactis cupripennis* | 0 | 1 | 0 | 0 | [1, 2] | 8 | -16 | 24 | [3] | 2900 | 3400 | 500 | [7] | South America | Ancestral State Reconstruction | [8] |  |  | 7.517 | 0.426 |  |  | 2 | [6], Núñez L. (unpublished data) |
| Brilliants | *Aglaeactis pamela* | 0 | 1 | 0 | 0 | [1, 2] | -16 | -18 | 2 | [3] | 3000 | 3500 | 500 | [2] | South America* | Ancestral State Reconstruction | [8] |  |  | 7.625 | 0.675 |  |  | 2 | [6], Núñez L. (unpublished data) |
| Brilliants | *Boissonneaua flavescens* | 0 | 1 | 0 | 1 | [1, 2] | 9 | 0 | 9 | [3] | 1400 | 2800 | 1400 | [7] | South America | Ancestral State Reconstruction | [8] | 17.252 | 1.009 | 8.275 | 0.377 | 75.618 | 3.420 | 2 | [6], [3], Núñez L. (unpublished data) |
| Brilliants | *Boissonneaua jardini* | 0 | 1 | 0 | 1 | [1, 2] | 6 | 0 | 6 | [3] | 350 | 2200 | 1850 | [7] | South America* | Ancestral State Reconstruction | [8] | 18.450 | 0.250 | 8.615 | 0.580 | 73.800 | 2.800 | 2 | [6], Núñez L. (unpublished data) |
| Brilliants | *Clytolaema rubricauda* | 0 | 1 | 0 | 0 | [1, 2] | -12 | -32 | 20 | [3] | 0 | 2410 | 2410 | [7] | South America* | Ancestral State Reconstruction | [8] |  |  | 7.567 | 1.277 |  |  | 2 | [6], Núñez L. (unpublished data) |
| Brilliants | *Coeligena bonapartei* | 0 | 1 | 0 | 0 | [1, 2] | 9 | 6 | 3 | [3] | 1400 | 3200 | 1800 | [7] | South America* | Ancestral State Reconstruction | [8] | 29.003 | 1.957 | 6.650 | 0.354 | 71.738 | 3.037 | 2 | [6], [3] |
| Brilliants | *Coeligena coeligena* | 0 | 1 | 0 | 0 | [1, 2] | 10 | -18 | 28 | [3] | 1000 | 2600 | 1600 | [7] | South America | Ancestral State Reconstruction | [8] | 29.807 | 1.565 | 6.975 | 0.888 | 70.910 | 3.286 | 2 | [6], [3], Núñez L. (unpublished data) |
| Brilliants | *Coeligena helianthea* | 0 | 1 | 0 | 0 | [1, 2] | 8 | 6 | 2 | [3] | 1900 | 3300 | 1400 | [7] | South America* | Ancestral State Reconstruction | [8] | 30.079 | 1.638 | 6.762 | 0.593 | 70.687 | 2.147 | 2 | [6], [3], Núñez L. (unpublished data) |
| Brilliants | *Coeligena iris* | 0 | 1 | 0 | 0 | [1, 2] | 0 | -6 | 6 | [3] | 1700 | 3500 | 1800 | [7] | South America* | Ancestral State Reconstruction | [8] | 28.726 | 0.662 | 8.400 | 0.361 | 77.242 | 4.569 | 2 | [6], [3] |
| Brilliants | *Coeligena lutetiae* | 0 | 1 | 0 | 0 | [1, 2] | 4 | -8 | 12 | [3] | 2600 | 3600 | 1000 | [7] | South America | Ancestral State Reconstruction | [8] | 32.462 | 2.718 | 6.680 | 0.342 | 73.099 | 3.302 | 2 | [6], [3], Núñez L. (unpublished data) |
| Brilliants | *Coeligena prunellei* | 0 | 1 | 0 | 0 | [1, 2] | 8 | 5 | 3 | [3] | 1400 | 2600 | 1200 | [7] | South America* | Ancestral State Reconstruction | [8] | 27.325 | 1.952 | 6.800 | 0.283 | 70.797 | 0.894 | 2 | [6], [3] |
| Brilliants | *Coeligena torquata* | 0 | 1 | 0 | 1 | [1, 2] | 8 | -18 | 26 | [3] | 1500 | 3000 | 1500 | [7] | South America | Ancestral State Reconstruction | [8] | 33.575 | 1.915 | 7.043 | 0.444 | 73.955 | 3.379 | 2 | [6], [3], Núñez L. (unpublished data) |
| Brilliants | *Coeligena violifer* | 0 | 1 | 0 | 0 | [1, 2] | -14 | -18 | 4 | [3] | 2800 | 3300 | 500 | [2] | South America | Ancestral State Reconstruction | [8] | 33.008 | 2.214 | 7.606 | 0.846 | 75.321 | 4.043 | 2 | [6], [3], Núñez L. (unpublished data) |
| Brilliants | *Coeligena wilsoni* | 0 | 1 | 0 | 0 | [1, 2] | 6 | 0 | 6 | [3] | 700 | 2000 | 1300 | [7] | South America | Ancestral State Reconstruction | [8] | 32.510 | 1.060 | 7.405 | 1.284 | 68.005 | 2.435 | 2 | [6], Núñez L. (unpublished data) |
| Brilliants | *Ensifera ensifera* | 0 | 1 | 0 | 0 | [1, 2] | 8 | -18 | 26 | [3] | 1700 | 3300 | 1600 | [7] | South America | Ancestral State Reconstruction | [8] |  |  | 13.500 | 2.121 |  |  | 1 | [6] |
| Brilliants | *Eriocnemis cupreoventris* | 0 | 1 | 0 | 0 | [1, 2] | 8 | 6 | 2 | [3] | 1950 | 3000 | 1050 | [7] | South America* | Ancestral State Reconstruction | [8] | 17.785 | 0.105 | 5.303 | 0.335 | 58.400 | 1.130 | 2 | [6], Núñez L. (unpublished data) |
| Brilliants | *Eriocnemis derbyi* | 0 | 1 | 0 | 0 | [1, 2] | 6 | 0 | 6 | [3] | 2500 | 3600 | 1100 | [7] | South America* | Ancestral State Reconstruction | [8] |  |  |  |  |  |  | 2 | [6] |
| Brilliants | *Eriocnemis luciani* | 0 | 1 | 0 | 0 | [1, 2] | 2 | -8 | 10 | [3] | 2800 | 4800 | 2000 | [7] | South America | Ancestral State Reconstruction | [8] |  |  | 6.075 | 0.457 |  |  | 2 | [6], Núñez L. (unpublished data) |
| Brilliants | *Eriocnemis mirabilis* | 0 | 1 | 0 | 0 | [1, 2] | 3 | 2 | 1 | [3] | 2200 | 2200 | 0 | [7] | South America* | Ancestral State Reconstruction | [8] |  |  |  |  |  |  | 2 | [6] |
| Brilliants | *Eriocnemis mosquera* | 0 | 1 | 0 | 0 | [1, 2] | 6 | 0 | 6 | [3] | 1200 | 3600 | 2400 | [7] | South America | Ancestral State Reconstruction | [8] |  |  | 5.500 | 0.424 |  |  | 2 | [6] |
| Brilliants | *Eriocnemis nigrivestis* | 0 | 1 | 0 | 0 | [1, 2] | 2 | -5 | 7 | [3] | 2750 | 4700 | 1950 | [7] | South America* | Ancestral State Reconstruction | [8] |  |  | 4.450 | 0.212 |  |  | 2 | [6] |
| Brilliants | *Eriocnemis vestita* | 0 | 1 | 0 | 0 | [1, 2] | 8 | -6 | 14 | [3] | 2250 | 3850 | 1600 | [7] | South America | Ancestral State Reconstruction | [8] | 18.610 | 0.930 | 4.660 | 0.368 | 56.505 | 0.855 | 2 | [6], Núñez L. (unpublished data) |
| Brilliants | *Haplophaedia aureliae* | 0 | 1 | 0 | 0 | [1, 2] | 9 | -17 | 26 | [3] | 1500 | 3100 | 1600 | [7] | South America | Ancestral State Reconstruction | [8] | 17.855 | 0.115 | 5.003 | 1.020 | 56.985 | 1.765 | 2 | [6], Núñez L. (unpublished data) |
| Brilliants | *Haplophaedia lugens* | 0 | 1 | 0 | 0 | [1, 2] | 2 | -1 | 3 | [3] | 1100 | 2000 | 900 | [7] | South America | Ancestral State Reconstruction | [8] | 20.250 | 0.250 | 5.500 | 0.707 | 63.500 | 1.600 | 2 | [6], Núñez L. (unpublished data) |
| Brilliants | *Heliodoxa gularis* | 0 | 1 | 0 | 0 | [1, 2] | 2 | -4 | 6 | [3] | 600 | 1100 | 500 | [7] | South America* | Ancestral State Reconstruction | [8] | 25.298 | 1.135 |  |  | 61.093 | 3.033 | 3 | [6], [3] |
| Brilliants | *Heliodoxa imperatrix* | 0 | 1 | 0 | 0 | [1, 2] | 5 | 0 | 5 | [3] | 400 | 2050 | 1650 | [7] | South America | Ancestral State Reconstruction | [8] | 24.193 | 0.337 | 8.074 | 0.956 | 64.863 | 4.650 | 2 | [6], [3], Núñez L. (unpublished data) |
| Brilliants | *Heliodoxa jacula* | 0 | 1 | 0 | 0 | [1, 2] | 10 | 0 | 10 | [3] | 500 | 2300 | 1800 | [7] | South America | Ancestral State Reconstruction | [8] | 21.748 | 0.886 | 7.228 | 0.621 | 68.128 | 5.330 | 2 | [6], [3], Núñez L. (unpublished data) |
| Brilliants | *Heliodoxa rubinoides* | 0 | 1 | 0 | 0 | [1, 2] | 6 | -6 | 12 | [3] | 1200 | 2600 | 1400 | [7] | South America | Ancestral State Reconstruction | [8] | 22.200 | 1.300 | 8.450 | 0.354 | 68.960 | 0.900 | 3 | [6], Núñez L. (unpublished data) |
| Brilliants | *Heliodoxa xanthogonys* | 0 | 1 | 0 | 0 | [1, 2] | 7 | 0 | 7 | [3] | 700 | 2000 | 1300 | [7] | South America | Ancestral State Reconstruction | [8] | 18.752 | 1.881 | 6.817 | 0.426 | 65.348 | 2.608 | 2 | [6], [3] |
| Brilliants | *Lafresnaya lafresnayi* | 0 | 1 | 0 | 0 | [1, 2] | 9 | -12 | 21 | [3] | 1500 | 3700 | 2200 | [7] | South America | Ancestral State Reconstruction | [8] |  |  | 5.400 | 0.735 |  |  | 4 | [6], Núñez L. (unpublished data) |
| Brilliants | *Loddigesia mirabilis* | 0 | 1 | 0 | 0 | [1, 2] | -6 | -8 | 2 | [3] | 2100 | 2900 | 800 | [2] | South America* | Ancestral State Reconstruction | [8] |  |  | 3.000 |  |  |  | 2 | [6] |
| Brilliants | *Ocreatus underwoodii* | 0 | 1 | 0 | 0 | [1, 2] | 11 | -18 | 29 | [3] | 850 | 3100 | 2250 | [7] | South America | Ancestral State Reconstruction | [8] | 13.495 | 0.335 | 2.850 | 0.253 | 41.325 | 1.025 | 2 | [6], Núñez L. (unpublished data) |
| Brilliants | *Pterophanes cyanopterus* | 0 | 1 | 0 | 0 | [1, 2] | 8 | -18 | 26 | [3] | 2600 | 3600 | 1000 | [7] | South America | Ancestral State Reconstruction | [8] |  |  | 10.375 | 0.767 |  |  | 2 | [6], Núñez L. (unpublished data) |
| Brilliants | *Urochroa bougueri* | 0 | 1 | 0 | 0 | [1, 2] | 4 | -4 | 8 | [3] | 500 | 2500 | 2000 | [7] | South America | Ancestral State Reconstruction | [8] |  |  | 8.850 | 0.495 |  |  | 2 | [6] |
| Brilliants | *Urosticte benjamini* | 0 | 1 | 0 | 0 | [1, 2] | 6 | 0 | 6 | [3] | 700 | 1500 | 800 | [7] | South America | Ancestral State Reconstruction | [8] |  |  | 4.000 | 0.283 |  |  | 2 | [6] |
| Brilliants | *Urosticte ruficrissa* | 0 | 1 | 0 | 0 | [1, 2] | 2 | -6 | 8 | [3] | 1600 | 2300 | 700 | [7] | South America* | Ancestral State Reconstruction | [8] |  |  | 4.100 | 0.141 |  |  | 2 | [6] |
| Coquettes | *Adelomyia melanogenys* | 0 | 1 | 0 | 1 | [1, 2] | 10 | -25 | 35 | [3] | 1000 | 2500 | 1500 | [7] | South America | Ancestral State Reconstruction | [8] | 13.815 | 0.025 | 4.100 | 0.625 | 51.175 | 2.075 | 2 | [6], Núñez L. (unpublished data) |
| Coquettes | *Aglaiocercus berlepschi* | 0 | 1 | 0 | 0 | [1, 2] | 12 | 12 | 0.2 | [3] | 1450 | 1800 | 350 | [2] | South America* | Ancestral State Reconstruction | [8] |  |  | 5.000 | 0.707 |  |  | 2 | [6] |
| Coquettes | *Aglaiocercus coelestis* | 0 | 1 | 0 | 0 | [1, 2] | 10 | 0 | 10 | [3] | 300 | 2100 | 1800 | [7] | South America | Ancestral State Reconstruction | [8] | 15.135 | 0.135 | 5.125 | 0.709 | 61.860 | 5.860 | 2 | [6], Núñez L. (unpublished data) |
| Coquettes | *Aglaiocercus kingi* | 0 | 1 | 0 | 0 | [1, 2] | 10 | -18 | 28 | [3] | 900 | 3000 | 2100 | [7] | South America | Ancestral State Reconstruction | [8] |  |  | 5.540 | 0.639 |  |  | 2 | [6], Núñez L. (unpublished data) |
| Coquettes | *Chalcostigma herrani* | 0 | 1 | 0 | 0 | [1, 2] | 6 | -7 | 13 | [3] | 2700 | 3600 | 900 | [7] | South America | Ancestral State Reconstruction | [8] |  |  | 5.950 | 0.636 |  |  | 2 | [6] |
| Coquettes | *Chalcostigma heteropogon* | 0 | 1 | 0 | 0 | [1, 2] | 7 | 6 | 1 | [3] | 2900 | 3500 | 600 | [7] | South America* | Ancestral State Reconstruction | [8] |  |  | 6.100 | 0.283 |  |  | 2 | [6] |
| Coquettes | *Chalcostigma olivaceum* | 0 | 1 | 0 | 0 | [1, 2] | -12 | -16 | 4 | [3] | 3600 | 4600 | 1000 | [2] | South America* | Ancestral State Reconstruction | [8] |  |  | 8.500 | 0.707 |  |  | 2 | [6] |
| Coquettes | *Chalcostigma ruficeps* | 0 | 1 | 0 | 0 | [1, 2] | 4 | -18 | 22 | [3] | 2100 | 2700 | 600 | [7] | South America | Ancestral State Reconstruction | [8] |  |  | 3.600 | 0.424 |  |  | 2 | [6] |
| Coquettes | *Chalcostigma stanleyi* | 0 | 1 | 0 | 0 | [1, 2] | -1 | -18 | 17 | [3] | 3500 | 4500 | 1000 | [7] | South America* | Ancestral State Reconstruction | [8] |  |  | 5.350 | 1.202 |  |  | 2 | [6] |
| Coquettes | *Discosura conversii* | 0 | 1 | 0 | 0 | [1, 2] | 10 | -3 | 13 | [3] | 0 | 1400 | 1400 | [7] | South America | Ancestral State Reconstruction | [8] |  |  | 3.000 |  |  |  | 2 | [6] |
| Coquettes | *Discosura langsdorffi* | 0 | 1 | 0 | 0 | [1, 2] | 3 | -22 | 25 | [3] | 0 | 300 | 300 | [7] | South America* | Ancestral State Reconstruction | [8] | 11.550 | 0.350 | 2.733 | 0.503 |  |  | 2 | [6], Núñez L. (unpublished data) |
| Coquettes | *Discosura longicaudus* | 0 | 1 | 0 | 0 | [1, 2] | 8 | -22 | 30 | [3] | 0 | 200 | 200 | [7] | South America* | Ancestral State Reconstruction | [8] |  |  | 3.350 | 0.495 |  |  | 2 | [6] |
| Coquettes | *Discosura popelairii* | 0 | 1 | 0 | 0 | [1, 2] | 6 | -13 | 19 | [3] | 500 | 1200 | 700 | [7] | South America | Ancestral State Reconstruction | [8] |  |  | 2.500 |  |  |  | 2 | [6] |
| Coquettes | *Heliangelus amethysticollis* | 0 | 1 | 0 | 0 | [1, 2] | 9 | -18 | 27 | [3] | 1800 | 3200 | 1400 | [2] | South America | Ancestral State Reconstruction | [8] | 17.901 | 0.730 | 5.550 | 0.590 | 64.714 | 3.096 | 2 | [6], [3], Núñez L. (unpublished data) |
| Coquettes | *Heliangelus exortis* | 0 | 1 | 0 | 0 | [1, 2] | 7 | -6 | 13 | [3] | 1500 | 3400 | 1900 | [7] | South America* | Ancestral State Reconstruction | [8] | 15.672 | 0.761 | 4.240 | 0.611 | 60.635 | 4.422 | 2 | [6], [3], Núñez L. (unpublished data) |
| Coquettes | *Heliangelus mavors* | 0 | 1 | 0 | 0 | [1, 2] | 10 | 6 | 4 | [3] | 2000 | 3200 | 1200 | [7] | South America* | Ancestral State Reconstruction | [8] | 14.247 | 0.402 | 4.150 | 0.354 | 60.543 | 3.504 | 2 | [6], [3] |
| Coquettes | *Heliangelus regalis* | 0 | 1 | 0 | 0 | [1, 2] | -6 | -8 | 2 | [3] | 1950 | 2200 | 250 | [7] | South America* | Ancestral State Reconstruction | [8] |  |  | 4.000 | 0.707 |  |  | 2 | [6] |
| Coquettes | *Heliangelus viola* | 0 | 1 | 0 | 0 | [1, 2] | 0 | -7 | 7 | [3] | 2000 | 3500 | 1500 | [7] | South America* | Ancestral State Reconstruction | [8] | 15.055 | 0.425 | 6.010 | 0.800 | 65.470 | 2.030 | 2 | [6], [3] |
| Coquettes | *Lesbia nuna* | 0 | 1 | 0 | 1 | [1, 2] | 8 | -18 | 26 | [3] | 2000 | 3800 | 1800 | [7] | South America | Ancestral State Reconstruction | [8] | 17.050 | 0.450 | 3.800 |  | 53.750 | 2.150 | 2 | [6], Núñez L. (unpublished data) |
| Coquettes | *Lesbia victoriae* | 0 | 1 | 0 | 1 | [1, 2] | 7 | -14 | 21 | [3] | 2600 | 4000 | 1400 | [7] | South America | Ancestral State Reconstruction | [8] | 18.900 | 0.200 | 4.900 | 0.346 | 57.600 | 2.300 | 2 | [6], Núñez L. (unpublished data) |
| Coquettes | *Lophornis adorabilis* | 0 | 1 | 0 | 0 | [1, 2] | 10 | 8 | 2 | [3] | 300 | 1200 | 900 | [2] | North America* | Ancestral State Reconstruction | [8] | 10.828 | 0.384 | 2.700 |  | 37.500 | 1.470 | 2 | [6], [3] |
| Coquettes | *Lophornis brachylophus* | 0 | 1 | 0 | 0 | [1, 2] | 17 | 17 | 0.2 | [3] | 900 | 1800 | 900 | [2] | North America* | Ancestral State Reconstruction | [8] | 9.967 | 0.499 | 2.667 | 0.306 | 44.700 | 2.112 | 2 | [6], [3] |
| Coquettes | *Lophornis chalybeus* | 0 | 1 | 0 | 0 | [1, 2] | 6 | -36 | 42 | [3] | 100 | 600 | 500 | [7] | South America* | Ancestral State Reconstruction | [8] | 13.334 | 0.756 | 2.800 | 0.346 | 41.898 | 1.905 | 2 | [6], [3], Núñez L. (unpublished data) |
| Coquettes | *Lophornis delattrei* | 0 | 1 | 0 | 0 | [1, 2] | 8 | -18 | 26 | [3] | 600 | 2000 | 1400 | [7] | South America | Ancestral State Reconstruction | [8] | 11.600 | 0.200 | 2.800 | 0.200 | 37.500 | 0.600 | 2 | [6], Núñez L. (unpublished data) |
| Coquettes | *Lophornis helenae* | 1 | 1 | 0 | 0 | [1, 2] | 17 | 10 | 7 | [3] | 350 | 1450 | 1100 | [7] | North America* | Ancestral State Reconstruction | [8] | 11.455 | 0.503 | 2.700 | 0.141 | 40.873 | 0.954 | 2 | [6], [3] |
| Coquettes | *Lophornis magnificus* | 0 | 1 | 0 | 0 | [1, 2] | -15 | -20 | 5 | [3] | 0 | 1000 | 1000 | [2] | South America* | Ancestral State Reconstruction | [8] |  |  | 3.000 |  |  |  | 2 | [6] |
| Coquettes | *Lophornis ornatus* | 0 | 1 | 0 | 0 | [1, 2] | 11 | 2 | 9 | [3] | 100 | 950 | 850 | [7] | South America* | Ancestral State Reconstruction | [8] |  |  | 2.550 | 0.354 |  |  | 2 | [6] |
| Coquettes | *Lophornis pavoninus* | 0 | 1 | 0 | 0 | [1, 2] | 6 | 5 | 1 | [3] | 500 | 2000 | 1500 | [7] | South America | Ancestral State Reconstruction | [8] |  |  |  |  |  |  | 2 | [6] |
| Coquettes | *Lophornis stictolophus* | 0 | 1 | 0 | 0 | [1, 2] | 6 | -5 | 11 | [3] | 0 | 1300 | 1300 | [7] | South America* | Ancestral State Reconstruction | [8] | 10.230 | 0.784 |  |  | 36.118 | 1.319 | 2 | [6], [3] |
| Coquettes | *Metallura aeneocauda* | 0 | 1 | 0 | 0 | [1, 2] | -14 | -18 | 4 | [3] | 3000 | 3600 | 600 | [2] | South America | Ancestral State Reconstruction | [8] |  |  | 5.300 | 0.141 |  |  | 2 | [6] |
| Coquettes | *Metallura baroni* | 0 | 1 | 0 | 0 | [1, 2] | 2 | -5 | 7 | [3] | 1900 | 1900 | 0 | [7] | South America* | Ancestral State Reconstruction | [8] |  |  | 4.350 | 0.212 |  |  | 2 | [6] |
| Coquettes | *Metallura odomae* | 0 | 1 | 0 | 0 | [1, 2] | -7 | -8 | 1 | [3] | 2600 | 3350 | 750 | [2] | South America* | Ancestral State Reconstruction | [8] |  |  | 5.000 | 0.283 |  |  | 2 | [6] |
| Coquettes | *Metallura phoebe* | 0 | 1 | 0 | 1 | [1, 2] | -7 | -18 | 11 | [3] | 1980 | 3050 | 1070 | [7] | South America | Ancestral State Reconstruction | [8] |  |  | 5.750 | 0.212 |  |  | 2 | [6] |
| Coquettes | *Metallura theresiae* | 0 | 1 | 0 | 0 | [1, 2] | 0 | -8 | 8 | [3] | 2900 | 3800 | 900 | [2] | South America* | Ancestral State Reconstruction | [8] |  |  | 4.900 | 0.141 |  |  | 2 | [6] |
| Coquettes | *Metallura tyrianthina* | 0 | 1 | 0 | 1 | [1, 2] | 10 | -18 | 28 | [3] | 1700 | 3800 | 2100 | [7] | South America | Ancestral State Reconstruction | [8] |  |  | 3.500 | 0.141 |  |  | 2 | [6] |
| Coquettes | *Metallura williami* | 0 | 1 | 0 | 0 | [1, 2] | 7 | 0 | 7 | [3] | 2100 | 3800 | 1700 | [7] | South America | Ancestral State Reconstruction | [8] |  |  | 4.525 | 0.247 |  |  | 2 | [6], Núñez L. (unpublished data) |
| Coquettes | *Opisthoprora euryptera* | 0 | 1 | 0 | 0 | [1, 2] | 6 | -8 | 14 | [3] | 2500 | 3600 | 1100 | [7] | South America | Ancestral State Reconstruction | [8] |  |  | 6.300 | 0.416 |  |  | 3 | [6] |
| Coquettes | *Oreonympha nobilis* | 0 | 1 | 0 | 0 | [1, 2] | -12 | -14 | 2 | [3] | 2500 | 3900 | 1400 | [2] | South America | Ancestral State Reconstruction | [8] |  |  | 9.000 |  |  |  | 2 | [6] |
| Coquettes | *Oreotrochilus adela* | 0 | 0 | 0 | 1 | [1, 2] | -16 | -20 | 4 | [3] | 2600 | 4000 | 1400 | [2] | South America* | Ancestral State Reconstruction | [8] |  |  | 7.850 | 0.636 |  |  | 3 | [6] |
| Coquettes | *Oreotrochilus chimborazo* | 0 | 1 | 0 | 0 | [1, 2] | 0 | -1 | 1 | [3] | 5300 | 5300 | 0 | [7] | South America | Ancestral State Reconstruction | [8] |  |  | 7.950 | 0.212 |  |  | 3 | [6] |
| Coquettes | *Oreotrochilus estella* | 0 | 1 | 0 | 1 | [1, 2] | -6 | -18 | 12 | [3] | 1830 | 4300 | 2470 | [7] | South America | Ancestral State Reconstruction | [8] |  |  | 8.400 | 0.566 |  |  | 3 | [6] |
| Coquettes | *Oreotrochilus leucopleurus* | 0 | 1 | 0 | 1 | [1, 2] | -21 | -35 | 14 | [3] | 1525 | 3660 | 2135 | [7] | South America* | Ancestral State Reconstruction | [8] |  |  | 8.150 | 0.354 |  |  | 3 | [6] |
| Coquettes | *Oreotrochilus melanogaster* | 0 | 1 | 0 | 1 | [1, 2] | -12 | -14 | 2 | [3] | 3500 | 4400 | 900 | [2] | South America* | Ancestral State Reconstruction | [8] |  |  | 8.400 |  |  |  | 3 | [6] |
| Coquettes | *Oxypogon guerinii* | 0 | 1 | 0 | 0 | [1, 2] | 9 | 6 | 3 | [3] | 3200 | 5200 | 2000 | [7] | South America | Ancestral State Reconstruction | [8] |  |  | 5.175 | 0.634 |  |  | 2 | [6], Núñez L. (unpublished data) |
| Coquettes | *Phlogophilus harterti* | 0 | 1 | 0 | 0 | [1, 2] | -10 | -16 | 6 | [3] | 750 | 1500 | 750 | [2] | South America* | Ancestral State Reconstruction | [8] |  |  | 2.450 | 0.204 |  |  | 2 | [6], Núñez L. (unpublished data) |
| Coquettes | *Phlogophilus hemileucurus* | 0 | 1 | 0 | 0 | [1, 2] | 1 | -8 | 9 | [3] | 400 | 1500 | 1100 | [7] | South America | Ancestral State Reconstruction | [8] |  |  | 2.600 | 0.566 |  |  | 2 | [6] |
| Coquettes | *Polyonymus caroli* | 0 | 1 | 0 | 1 | [1, 2] | -14 | -17 | 3 | [3] | 1500 | 3600 | 2100 | [2] | South America* | Ancestral State Reconstruction | [8] |  |  | 5.350 | 0.354 |  |  | 2 | [6] |
| Coquettes | *Ramphomicron dorsale* | 0 | 1 | 0 | 0 | [1, 2] | 11 | 10 | 1 | [3] | 2000 | 4500 | 2500 | [7] | South America* | Ancestral State Reconstruction | [8] |  |  | 3.500 |  |  |  | 2 | [6] |
| Coquettes | *Ramphomicron microrhynchum* | 0 | 1 | 0 | 0 | [1, 2] | 8 | -18 | 26 | [3] | 1700 | 3400 | 1700 | [7] | South America | Ancestral State Reconstruction | [8] |  |  | 3.500 |  |  |  | 2 | [6] |
| Coquettes | *Sappho sparganura* | 0 | 1 | 0 | 1 | [1, 2] | -20 | -40 | 20 | [3] | 1500 | 4000 | 2500 | [2] | South America* | Ancestral State Reconstruction | [8] |  |  | 5.550 | 0.495 |  |  | 2 | [6] |
| Coquettes | *Sephanoides fernandensis* | 0 | 0 | 0 | 1 | [1, 2] | -34 | -34 | 0.2 | [3] | 0 | 0 | 0 | [7] | South America | Ancestral State Reconstruction | [8] | 15.105 | 0.399 | 8.900 | 2.311 | 76.685 | 4.622 | 2 | [6], [3], Núñez L. (unpublished data) |
| Coquettes | *Sephanoides sephanoides* | 0 | 0 | 0 | 1 | [1, 2] | -24 | -54 | 30 | [3] | 0 | 2135 | 2135 | [7] | South America | Ancestral State Reconstruction | [8] | 15.524 | 0.929 | 5.225 | 0.457 | 60.580 | 2.597 | 2 | [6], [3], Núñez L. (unpublished data) |
| Coquettes | *Taphrolesbia griseiventris* | 0 | 1 | 0 | 0 | [1, 2] | -7 | -10 | 3 | [3] | 2600 | 3500 | 900 | [2] | South America* | Ancestral State Reconstruction | [8] | 2.200 | 0.000 |  |  |  |  | 2 | [6] |
| Emeralds | *Abeillia abeillei* | 0 | 1 | 0 | 0 | [1, 2] | 17 | 13 | 4 | [3] | 1000 | 1850 | 850 | [7] | North America | Ancestral State Reconstruction | [5] | 11.193 | 0.455 | 2.700 | 0.000 | 45.105 | 2.015 | 2 | [6], [3], Núñez L. (unpublished data) |
| Emeralds | *Amazilia amabilis* | 0 | 1 | 0 | 0 | [1, 2] | 13 | -2 | 15 | [3] | 0 | 1580 | 1580 | [7] | South America | Bayesian dispersal–vicariance analysis (S-DIVA) | [9] | 18.241 | 0.939 | 3.886 | 0.225 | 51.568 | 2.887 | 2 | [6], [3], Núñez L. (unpublished data) |
| Emeralds | *Amazilia amazilia* | 0 | 1 | 0 | 1 | [1, 2] | 1 | -15 | 16 | [3] | 0 | 2500 | 2500 | [7] | South America | Bayesian dispersal–vicariance analysis (S-DIVA) | [9] | 18.508 | 0.653 | 4.789 | 0.509 | 59.200 | 2.273 | 2 | [6], [3], Núñez L. (unpublished data) |
| Emeralds | *Amazilia beryllina* | 1 | 1 | 0 | 0 | [1, 2] | 32 | 15 | 17 | [3] | 0 | 3000 | 3000 | [7] | North America | Ancestral State Reconstruction | [5] | 19.273 | 1.062 | 4.434 | 0.453 | 53.903 | 2.620 | 2 | [6], [3], Núñez L. (unpublished data) |
| Emeralds | *Amazilia boucardi* | 0 | 1 | 0 | 0 | [1, 2] | 11 | 8 | 3 | [3] | 0 | 200 | 200 | [2] | North America* | Ancestral State Reconstruction | [8] | 19.280 | 0.875 | 4.500 |  | 52.581 | 1.745 | 2 | [6], [3] |
| Emeralds | *Amazilia brevirostris* | 0 | 1 | 0 | 0 | [1, 2] | 8 | 12 | 4 | [3] | 0 | 500 | 500 | [7] | South America* | Ancestral State Reconstruction | [8] | 18.923 | 1.240 | 4.500 | 0.115 | 49.066 | 2.846 | 2 | [6], [3], Núñez L. (unpublished data) |
| Emeralds | *Amazilia candida* | 1 | 1 | 0 | 0 | [1, 2] | 21 | -15 | 36 | [3] | 0 | 800 | 800 | [7] | North America | Ancestral State Reconstruction | [5] | 17.011 | 0.990 | 3.758 | 0.503 | 50.031 | 2.423 | 2 | [6], [3], Núñez L. (unpublished data) |
| Emeralds | *Amazilia castaneiventris* | 0 | 1 | 0 | 0 | [1, 2] | 10 | 9 | 1 | [3] | 150 | 2045 | 1895 | [7] | South America* | Bayesian dispersal–vicariance analysis (S-DIVA) | [9] |  |  |  |  |  |  | 2 | [6] |
| Emeralds | *Amazilia chionogaster* | 0 | 1 | 0 | 1 | [1, 2] | -4 | -28 | 24 | [3] | 450 | 2000 | 1550 | [2] | South America | Bayesian dispersal–vicariance analysis (S-DIVA) | [9] | 22.767 | 1.423 | 5.120 | 0.567 | 57.013 | 2.532 | 2 | [6], [3], Núñez L. (unpublished data) |
| Emeralds | *Amazilia cyanifrons* | 0 | 1 | 0 | 0 | [1, 2] | 8 | 3 | 5 | [3] | 400 | 2000 | 1600 | [7] | South America* | Bayesian dispersal–vicariance analysis (S-DIVA) | [9] | 17.436 | 0.730 | 5.000 |  | 52.771 | 1.880 | 2 | [6], [3] |
| Emeralds | *Amazilia cyanocephala* | 1 | 1 | 0 | 0 | [1, 2] | 23 | 15 | 8 | [3] | 0 | 2550 | 2550 | [7] | North America | Ancestral State Reconstruction | [5] | 20.086 | 1.047 | 5.462 | 0.717 | 57.632 | 2.803 | 2 | [6], [3], Núñez L. (unpublished data) |
| Emeralds | *Amazilia cyanura* | 0 | 1 | 0 | 0 | [1, 2] | 16 | 14 | 2 | [3] | 0 | 1050 | 1050 | [7] | North America | Bayesian dispersal–vicariance analysis (S-DIVA) | [9] | 18.520 | 0.971 | 3.960 | 0.351 | 52.172 | 2.490 | 2 | [6], [3] |
| Emeralds | *Amazilia decora* | 0 | 1 | 0 | 0 | [1, 2] | 9 | 7 | 2 | [3] | 0 | 1200 | 1200 | [2] | South America | Bayesian dispersal–vicariance analysis (S-DIVA) | [9] | 21.470 | 0.955 | 3.950 | 0.212 | 51.334 | 2.272 | 2 | [6], [3] |
| Emeralds | *Amazilia edward* | 0 | 1 | 0 | 0 | [1, 2] | 9 | 8 | 1 | [3] | 0 | 1830 | 1830 | [7] | North America | Bayesian dispersal–vicariance analysis (S-DIVA) | [9] | 18.066 | 0.731 | 4.700 | 0.424 | 51.496 | 2.159 | 2 | [6], [3] |
| Emeralds | *Amazilia fimbriata* | 0 | 1 | 0 | 0 | [1, 2] | 8 | -30 | 38 | [3] | 0 | 1300 | 1300 | [7] | South America | Bayesian dispersal–vicariance analysis (S-DIVA) | [9] | 19.212 | 0.991 | 4.767 | 0.201 | 52.720 | 2.444 | 2 | [6], [3], Núñez L. (unpublished data) |
| Emeralds | *Amazilia franciae* | 0 | 1 | 0 | 0 | [1, 2] | 6 | -7 | 13 | [3] | 1000 | 2000 | 1000 | [7] | South America | Bayesian dispersal–vicariance analysis (S-DIVA) | [9] | 22.772 | 0.662 | 5.134 | 0.366 | 53.724 | 2.617 | 2 | [6], [3], Núñez L. (unpublished data) |
| Emeralds | *Amazilia lactea* | 0 | 1 | 0 | 0 | [1, 2] | 6 | -24 | 30 | [3] | 300 | 1400 | 1100 | [7] | South America | Bayesian dispersal–vicariance analysis (S-DIVA) | [9] | 18.185 | 0.809 | 4.325 | 0.614 | 52.016 | 1.757 | 2 | [6], [3], Núñez L. (unpublished data) |
| Emeralds | *Amazilia leucogaster* | 0 | 1 | 0 | 0 | [1, 2] | 9 | -11 | 20 | [3] | 0 | 250 | 250 | [7] | South America* | Ancestral State Reconstruction | [8] | 21.187 | 1.030 | 4.400 | 0.115 | 51.813 | 1.818 | 3 | [6], [3], Núñez L. (unpublished data) |
| Emeralds | *Amazilia luciae* | 0 | 1 | 0 | 0 | [1, 2] | 15 | 14 | 1 | [3] | 75 | 345 | 270 | [7] | North America* | Bayesian dispersal–vicariance analysis (S-DIVA) | [9] |  |  |  |  |  |  | 2 | [6] |
| Emeralds | *Amazilia rosenbergi* | 0 | 1 | 0 | 0 | [1, 2] | 6 | 1 | 5 | [3] | 0 | 200 | 200 | [7] | South America* | Bayesian dispersal–vicariance analysis (S-DIVA) | [9] | 21.076 | 1.071 | 3.750 | 0.351 | 50.782 | 2.499 | 2 | [6], [3], Núñez L. (unpublished data) |
| Emeralds | *Amazilia rutila* | 1 | 1 | 0 | 0 | [1, 2] | 25 | 14 | 11 | [3] | 0 | 1350 | 1350 | [7] | North America | Ancestral State Reconstruction | [5] | 21.612 | 1.807 | 4.868 | 0.977 | 56.569 | 4.647 | 2 | [6], [3], Núñez L. (unpublished data) |
| Emeralds | *Amazilia saucerrottei* | 0 | 1 | 0 | 0 | [1, 2] | 12 | 8 | 4 | [3] | 0 | 3000 | 3000 | [7] | South America | Bayesian dispersal–vicariance analysis (S-DIVA) | [9] | 17.523 | 0.861 | 4.883 | 0.241 | 52.109 | 2.076 | 2 | [6], [3], Núñez L. (unpublished data) |
| Emeralds | *Amazilia tobaci* | 0 | 1 | 0 | 0 | [1, 2] | 11 | 8 | 3 | [3] | 0 | 1800 | 1800 | [7] | South America | Bayesian dispersal–vicariance analysis (S-DIVA) | [9] | 18.482 | 0.712 | 4.400 | 0.283 | 52.968 | 2.434 | 2 | [6], [3], Núñez L. (unpublished data) |
| Emeralds | *Amazilia tzacatl* | 1 | 1 | 0 | 0 | [1, 2] | 23 | 9 | 14 | [3] | 0 | 1800 | 1800 | [7] | North America | Bayesian dispersal–vicariance analysis (S-DIVA) | [9] | 20.749 | 1.308 | 4.408 | 0.543 | 55.518 | 2.903 | 2 | [6], [3], Núñez L. (unpublished data) |
| Emeralds | *Amazilia versicolor* | 0 | 1 | 0 | 0 | [1, 2] | 7 | -28 | 35 | [3] | 0 | 1700 | 1700 | [7] | South America | Bayesian dispersal–vicariance analysis (S-DIVA) | [9] | 16.762 | 0.764 | 3.519 | 0.398 | 48.247 | 1.962 | 2 | [6], [3], Núñez L. (unpublished data) |
| Emeralds | *Amazilia violiceps* | 1 | 1 | 0 | 0 | [1, 2] | 32 | 17 | 15 | [3] | 0 | 2250 | 2250 | [7] | North America | Bayesian dispersal–vicariance analysis (S-DIVA) | [9] | 22.250 | 1.116 | 5.185 | 0.516 | 56.152 | 3.818 | 2 | [6], [3], Núñez L. (unpublished data) |
| Emeralds | *Amazilia viridigaster* | 0 | 1 | 0 | 0 | [1, 2] | 8 | 0 | 8 | [3] | 0 | 2100 | 2100 | [7] | South America | Bayesian dispersal–vicariance analysis (S-DIVA) | [9] | 17.852 | 0.577 | 3.913 | 0.566 | 51.530 | 1.618 | 2 | [6], [3], Núñez L. (unpublished data) |
| Emeralds | *Amazilia yucatanensis* | 1 | 1 | 0 | 0 | [1, 2] | 27 | 16 | 11 | [3] | 0 | 600 | 600 | [7] | North America | Bayesian dispersal–vicariance analysis (S-DIVA) | [9] | 20.476 | 0.756 | 4.104 | 0.248 | 54.519 | 1.695 | 2 | [6], [3], Núñez L. (unpublished data) |
| Emeralds | *Aphantochroa cirrochloris* | 0 | 1 | 0 | 0 | [1, 2] | -10 | -20 | 10 | [3] | 800 | 1460 | 660 | [7] | South America | Ancestral State Reconstruction | [8] |  |  | 9.000 |  |  |  | 2 | [6] |
| Emeralds | *Campylopterus curvipennis* | 1 | 1 | 0 | 0 | [1, 2] | 22 | 16 | 6 | [3] | 0 | 350 | 350 | [7] | South America* | Ancestral State Reconstruction | [8] | 27.020 | 1.057 | 8.113 | 2.459 | 65.280 | 3.178 | 2 | [6], [3], Núñez L. (unpublished data) |
| Emeralds | *Campylopterus ensipennis* | 0 | 1 | 1 | 0 | [1, 2] | 11 | 10 | 1 | [3] | 700 | 2000 | 1300 | [7] | South America* | Ancestral State Reconstruction | [8] | 26.119 | 2.331 | 9.750 | 0.354 | 66.189 | 20.046 | 3 | [6], [3], Núñez L. (unpublished data) |
| Emeralds | *Campylopterus falcatus* | 0 | 1 | 0 | 0 | [1, 2] | 10 | -1 | 11 | [3] | 900 | 3000 | 2100 | [7] | South America* | Ancestral State Reconstruction | [8] | 23.880 | 1.512 | 6.927 | 1.081 | 64.797 | 2.543 | 3 | [6], [3], Núñez L. (unpublished data) |
| Emeralds | *Campylopterus hemileucurus* | 0 | 1 | 0 | 0 | [1, 2] | 17 | 8 | 9 | [3] | 0 | 2450 | 2450 | [7] | South America | Ancestral State Reconstruction | [8] | 31.050 | 4.414 | 10.133 | 1.108 | 75.583 | 2.643 | 4 | [6], [3], Núñez L. (unpublished data) |
| Emeralds | *Campylopterus largipennis* | 0 | 1 | 0 | 0 | [1, 2] | 8 | -15 | 23 | [3] | 0 | 550 | 550 | [7] | South America | Ancestral State Reconstruction | [8] | 26.695 | 1.287 | 8.529 | 1.443 | 72.914 | 2.632 | 2 | [6], [3], Núñez L. (unpublished data) |
| Emeralds | *Campylopterus phainopeplus* | 0 | 1 | 0 | 0 | [1, 2] | 10 | 2 | 8 | [3] | 1200 | 4800 | 3600 | [7] | South America* | Bayesian dispersal–vicariance analysis (S-DIVA) | [9] |  |  |  |  |  |  | 3 | [6] |
| Emeralds | *Campylopterus rufus* | 0 | 1 | 0 | 0 | [1, 2] | 16 | 12 | 4 | [3] | 1250 | 1850 | 600 | [7] | North America | Ancestral State Reconstruction | [5] | 25.915 | 1.424 | 7.580 | 1.308 | 73.648 | 1.161 | 3 | [6], [3], Núñez L. (unpublished data) |
| Emeralds | *Campylopterus villaviscensio* | 0 | 1 | 0 | 0 | [1, 2] | 0 | -1 | 1 | [3] | 400 | 1500 | 1100 | [7] | South America | Ancestral State Reconstruction | [8] | 27.950 | 0.950 | 7.400 | 1.435 |  |  | 2 | [6], Núñez L. (unpublished data) |
| Emeralds | *Chalybura buffonii* | 0 | 1 | 0 | 0 | [1, 2] | 9 | -4 | 13 | [3] | 0 | 2000 | 2000 | [7] | South America | Ancestral State Reconstruction | [8] | 23.160 | 0.250 | 6.358 | 0.620 | 65.010 | 3.090 | 2 | [6], Núñez L. (unpublished data) |
| Emeralds | *Chalybura urochrysia* | 0 | 1 | 0 | 0 | [1, 2] | 14 | 8 | 6 | [3] | 0 | 900 | 900 | [7] | South America | Ancestral State Reconstruction | [8] | 23.135 | 0.445 | 6.348 | 0.934 | 72.940 | 3.610 | 2 | [6], Núñez L. (unpublished data) |
| Emeralds | *Chlorestes notata* | 0 | 1 | 0 | 0 | [1, 2] | 6 | -20 | 26 | [3] | 0 | 1000 | 1000 | [7] | South America | Ancestral State Reconstruction | [8] | 17.450 | 0.950 | 5.838 | 2.785 |  |  | 2 | [6], Núñez L. (unpublished data) |
| Emeralds | *Chlorostilbon alice* | 0 | 1 | 0 | 0 | [1, 2] | 11 | 9 | 2 | [3] | 750 | 1800 | 1050 | [7] | South America* | Ancestral State Reconstruction | [8] |  |  | 3.500 | 0.707 |  |  | 2 | [6] |
| Emeralds | *Chlorostilbon auriceps* | 1 | 1 | 0 | 0 | [1, 2] | 26 | 15 | 11 | [4] | 0 | 1800 | 1800 | [7] | North America* | Bayesian dispersal–vicariance analysis (S-DIVA) | [9] |  |  | 3.250 | 0.354 |  |  | 2 | [6] |
| Emeralds | *Chlorostilbon canivetii* | 1 | 1 | 0 | 0 | [1, 2] | 23 | 10 | 13 | [3] | 0 | 1850 | 1850 | [7] | North America | Ancestral State Reconstruction | [5] | 14.670 | 2.047 | 2.291 | 0.488 | 45.472 | 2.276 | 2 | [6], [3], Núñez L. (unpublished data) |
| Emeralds | *Chlorostilbon gibsoni* | 0 | 1 | 0 | 0 | [1, 2] | 10 | 6 | 4 | [3] | 0 | 2300 | 2300 | [7] | South America* | Ancestral State Reconstruction | [8] |  |  | 3.250 | 0.354 |  |  | 2 | [6] |
| Emeralds | *Chlorostilbon lucidus* | 0 | 1 | 0 | 1 | [1, 2] | -16 | -23 | 7 | [3] | 1160 | 1160 | 0 | [7] | South America* | Ancestral State Reconstruction | [8] |  |  | 4.000 | 0.707 |  |  | 2 | [6] |
| Emeralds | *Chlorostilbon maugaeus* | 0 | 0 | 1 | 0 | [1, 2] | 19 | 18 | 1 | [3] | 0 | 800 | 800 | [2] | Caribbean | Ancestral State Reconstruction | [5] | 13.500 | 0.500 | 3.275 | 0.411 | 47.700 | 0.800 | 2 | [6], Núñez L. (unpublished data) |
| Emeralds | *Chlorostilbon melanorhynchus* | 0 | 1 | 0 | 0 | [1, 2] | 7 | 2 | 5 | [10] | 600 | 2700 | 2100 | [2] | South America | Ancestral State Reconstruction | [8] |  |  | 3.250 | 0.354 |  |  | 2 | [6] |
| Emeralds | *Chlorostilbon mellisugus* | 0 | 1 | 0 | 0 | [1, 2] | 11 | -2 | 13 | [3] | 0 | 2200 | 2200 | [7] | South America | Ancestral State Reconstruction | [8] | 15.650 | 0.550 | 2.938 | 0.419 | 43.400 | 0.500 | 2 | [6], Núñez L. (unpublished data) |
| Emeralds | *Chlorostilbon olivaresi* | 0 | 1 | 0 | 0 | [1, 2] | 1 | 1 | 0.2 | [3] | 360 | 570 | 210 | [7] | South America* | Ancestral State Reconstruction | [8] |  |  | 3.700 | 0.141 |  |  | 2 | [6] |
| Emeralds | *Chlorostilbon poortmani* | 0 | 1 | 0 | 0 | [1, 2] | 8 | 5 | 3 | [3] | 500 | 2800 | 2300 | [7] | South America* | Ancestral State Reconstruction | [8] |  |  | 5.500 | 2.380 |  |  | 2 | [6], Núñez L. (unpublished data) |
| Emeralds | *Chlorostilbon ricordii* | 0 | 0 | 1 | 0 | [1, 2] | 27 | 21 | 6 | [3] | 0 | 1300 | 1300 | [2] | Caribbean | Ancestral State Reconstruction | [5] | 17.600 | 0.500 | 3.598 | 0.642 | 51.500 | 0.800 | 2 | [6], Núñez L. (unpublished data) |
| Emeralds | *Chlorostilbon russatus* | 0 | 1 | 0 | 0 | [1, 2] | 10 | 6 | 4 | [3] | 600 | 2600 | 2000 | [7] | South America* | Ancestral State Reconstruction | [8] |  |  | 3.400 | 0.283 |  |  | 2 | [6] |
| Emeralds | *Chlorostilbon stenurus* | 0 | 1 | 0 | 0 | [1, 2] | 9 | 8 | 1 | [3] | 1000 | 3000 | 2000 | [7] | South America* | Ancestral State Reconstruction | [8] |  |  | 3.400 | 0.283 |  |  | 2 | [6] |
| Emeralds | *Chlorostilbon swainsonii* | 0 | 0 | 1 | 0 | [1, 2] | 20 | 18 | 2 | [3] | 500 | 2500 | 2000 | [2] | Caribbean | Ancestral State Reconstruction | [5] |  |  | 3.750 | 1.768 |  |  | 2 | [6] |
| Emeralds | *Chrysuronia oenone* | 0 | 1 | 0 | 0 | [1, 2] | 8 | -5 | 13 | [3] | 0 | 1500 | 1500 | [7] | South America | Bayesian dispersal–vicariance analysis (S-DIVA) | [9] | 20.584 | 0.941 | 4.825 | 0.499 | 51.166 | 2.072 | 2 | [6], [3], Núñez L. (unpublished data) |
| Emeralds | *Cyanophaia bicolor* | 0 | 0 | 1 | 0 | [1, 2] | 19 | 12 | 7 | [3] | 800 | 1000 | 200 | [2] | Caribbean* | Ancestral State Reconstruction | [8] |  |  | 4.750 | 0.071 |  |  | 2 | [6] |
| Emeralds | *Cynanthus latirostris* | 1 | 1 | 0 | 0 | [1, 2] | 32 | 16 | 16 | [3] | 150 | 3000 | 2850 | [7] | North America | Ancestral State Reconstruction | [5] | 20.352 | 2.074 | 3.268 | 0.561 | 52.005 | 2.857 | 2 | [6], [3], Núñez L. (unpublished data) |
| Emeralds | *Cynanthus sordidus* | 1 | 1 | 0 | 0 | [1, 2] | 20 | 18 | 2 | [3] | 900 | 2200 | 1300 | [7] | North America | Ancestral State Reconstruction | [5] | 21.036 | 1.330 | 3.444 | 0.447 | 53.777 | 2.861 | 2 | [6], [3], Núñez L. (unpublished data) |
| Emeralds | *Damophila julie* | 0 | 1 | 0 | 0 | [1, 2] | 10 | -4 | 14 | [3] | 0 | 1750 | 1750 | [7] | South America | Bayesian dispersal–vicariance analysis (S-DIVA) | [9] | 14.000 | 1.100 | 3.250 | 0.472 | 43.350 | 0.450 | 2 | [6], Núñez L. (unpublished data) |
| Emeralds | *Elvira chionura* | 0 | 1 | 0 | 0 | [1, 2] | 11 | 7 | 4 | [3] | 750 | 1980 | 1230 | [7] | North America | Ancestral State Reconstruction | [8] |  |  | 3.200 | 0.141 |  |  | 2 | [6] |
| Emeralds | *Elvira cupreiceps* | 0 | 1 | 0 | 0 | [1, 2] | 11 | 8 | 3 | [3] | 700 | 1500 | 800 | [2] | North America | Ancestral State Reconstruction | [8] | 17.785 | 0.105 | 3.250 | 0.173 | 58.400 | 1.130 | 3 | [6], Núñez L. (unpublished data) |
| Emeralds | *Eupetomena macroura* | 0 | 1 | 0 | 0 | [1, 2] | -18 | -20 | 2 | [3] | 0 | 1500 | 1500 | [2] | South America* | Ancestral State Reconstruction | [8] |  |  | 7.558 | 1.249 |  |  | 3 | [6], Núñez L. (unpublished data) |
| Emeralds | *Eupherusa cyanophrys* | 0 | 1 | 0 | 0 | [1, 2] | 17 | 16 | 1 | [3] | 1200 | 2600 | 1400 | [7] | North America* | Ancestral State Reconstruction | [8] | 19.064 | 0.889 | 4.473 | 0.487 | 58.424 | 3.406 | 2 | [6], [3], Núñez L. (unpublished data) |
| Emeralds | *Eupherusa eximia* | 1 | 1 | 0 | 0 | [1, 2] | 19 | 8 | 11 | [3] | 0 | 2500 | 2500 | [7] | North America | Ancestral State Reconstruction | [8] | 17.397 | 1.066 | 4.320 | 0.278 | 57.322 | 2.630 | 2 | [6], [3], Núñez L. (unpublished data) |
| Emeralds | *Eupherusa nigriventris* | 0 | 1 | 0 | 0 | [1, 2] | 11 | 7 | 4 | [3] | 1370 | 2100 | 730 | [7] | North America | Ancestral State Reconstruction | [8] | 15.450 | 0.050 | 3.500 | 0.231 | 48.000 | 1.500 | 2 | [6], Núñez L. (unpublished data) |
| Emeralds | *Eupherusa poliocerca* | 0 | 1 | 0 | 0 | [1, 2] | 17 | 16 | 1 | [3] | 800 | 2300 | 1500 | [7] | North America* | Ancestral State Reconstruction | [8] | 18.691 | 0.949 | 4.398 | 0.494 | 59.184 | 3.444 | 2 | [6], [3], Núñez L. (unpublished data) |
| Emeralds | *Goethalsia bella* | 0 | 1 | 0 | 0 | [1, 2] | 8 | 7 | 1 | [3] | 600 | 1650 | 1050 | [7] | South America* | Ancestral State Reconstruction | [8] |  |  | 3.500 | 0.707 |  |  | 2 | [6] |
| Emeralds | *Goldmania violiceps* | 0 | 1 | 0 | 0 | [1, 2] | 8 | 7 | 1 | [3] | 600 | 1400 | 800 | [7] | South America* | Ancestral State Reconstruction | [8] |  |  | 3.950 | 0.636 |  |  | 2 | [6] |
| Emeralds | *Hylocharis chrysura* | 0 | 1 | 0 | 1 | [1, 2] | -18 | -36 | 18 | [3] | 400 | 800 | 400 | [2] | South America | Bayesian dispersal–vicariance analysis (S-DIVA) | [9] | 20.426 | 1.496 | 4.275 | 0.250 | 52.624 | 1.706 | 2 | [6], [3], Núñez L. (unpublished data) |
| Emeralds | *Hylocharis cyanus* | 0 | 1 | 0 | 0 | [1, 2] | 10 | -24 | 34 | [3] | 0 | 1250 | 1250 | [7] | South America | Bayesian dispersal–vicariance analysis (S-DIVA) | [9] | 17.454 | 0.703 | 3.567 | 0.208 | 46.593 | 2.307 | 2 | [6], [3], Núñez L. (unpublished data) |
| Emeralds | *Hylocharis eliciae* | 0 | 1 | 0 | 0 | [1, 2] | 18 | 6 | 12 | [3] | 0 | 350 | 350 | [7] | South America | Ancestral State Reconstruction | [8] | 17.437 | 0.822 | 3.913 | 0.242 | 47.823 | 1.605 | 2 | [6], [3], Núñez L. (unpublished data) |
| Emeralds | *Hylocharis grayi* | 0 | 1 | 0 | 0 | [1, 2] | 8 | 1 | 7 | [3] | 0 | 2600 | 2600 | [7] | South America | Ancestral State Reconstruction | [8] | 21.535 | 0.776 | 6.200 | 0.465 | 58.091 | 2.352 | 2 | [6], [3], Núñez L. (unpublished data) |
| Emeralds | *Hylocharis leucotis* | 1 | 1 | 0 | 0 | [1, 2] | 32 | 11 | 21 | [3] | 900 | 3900 | 3000 | [7] | North America | Ancestral State Reconstruction | [5] | 17.154 | 0.942 | 3.274 | 0.379 | 53.751 | 3.707 | 2 | [6], [3], Núñez L. (unpublished data) |
| Emeralds | *Hylocharis sapphirina* | 0 | 1 | 0 | 0 | [1, 2] | 2 | -28 | 30 | [3] | 0 | 500 | 500 | [7] | South America | Bayesian dispersal–vicariance analysis (S-DIVA) | [9] | 19.256 | 0.667 | 4.200 | 0.210 | 50.331 | 1.849 | 2 | [6], [3], Núñez L. (unpublished data) |
| Emeralds | *Hylocharis xantusii* | 1 | 0 | 0 | 0 | [1, 2] | 26 | 23 | 3 | [3] | 0 | 1500 | 1500 | [7] | North America* | Ancestral State Reconstruction | [8] | 17.382 | 0.692 | 3.258 | 0.809 | 50.648 | 1.582 | 2 | [6], [3] |
| Emeralds | *Klais guimeti* | 0 | 1 | 0 | 0 | [1, 2] | 14 | -18 | 32 | [3] | 400 | 1900 | 1500 | [7] | South America | Ancestral State Reconstruction | [8] | 11.450 | 0.150 | 2.838 | 0.111 | 47.250 | 2.350 | 2 | [6], Núñez L. (unpublished data) |
| Emeralds | *Lepidopyga coeruleogularis* | 0 | 1 | 0 | 0 | [1, 2] | 10 | 6 | 4 | [3] | 0 | 100 | 100 | [7] | South America | Bayesian dispersal–vicariance analysis (S-DIVA) | [9] |  |  | 4.100 | 0.283 |  |  | 2 | [6] |
| Emeralds | *Lepidopyga goudoti* | 0 | 1 | 0 | 0 | [1, 2] | 11 | 10 | 1 | [3] | 0 | 1600 | 1600 | [7] | South America* | Ancestral State Reconstruction | [8] | 18.505 | 1.025 | 3.818 | 0.269 | 46.925 | 0.355 | 2 | [6], Núñez L. (unpublished data) |
| Emeralds | *Leucippus baeri* | 0 | 1 | 0 | 0 | [1, 2] | -4 | -5 | 1 | [3] | 0 | 1300 | 1300 | [2] | South America | Bayesian dispersal–vicariance analysis (S-DIVA) | [9] | 20.050 | 0.750 | 4.375 | 0.171 |  |  | 2 | [6], Núñez L. (unpublished data) |
| Emeralds | *Leucippus chlorocercus* | 0 | 1 | 0 | 0 | [1, 2] | 0 | -1 | 1 | [3] | 0 | 430 | 430 | [7] | South America* | Ancestral State Reconstruction | [8] | 17.700 | 0.000 | 5.367 | 0.603 |  |  | 2 | [6], Núñez L. (unpublished data) |
| Emeralds | *Leucippus fallax* | 0 | 1 | 0 | 0 | [1, 2] | 11 | 10 | 1 | [3] | 0 | 800 | 800 | [7] | South America* | Ancestral State Reconstruction | [8] | 21.013 | 0.994 |  |  | 59.297 | 1.682 | 2 | [6], [3] |
| Emeralds | *Leucippus taczanowskii* | 0 | 1 | 0 | 1 | [1, 2] | -6 | -8 | 2 | [3] | 350 | 1000 | 650 | [2] | South America | Bayesian dispersal–vicariance analysis (S-DIVA) | [9] | 24.171 | 1.360 | 6.950 | 0.714 | 67.386 | 2.106 | 3 | [6], [3], Núñez L. (unpublished data) |
| Emeralds | *Leucochloris albicollis* | 0 | 1 | 0 | 1 | [1, 2] | -20 | -28 | 8 | [3] | 825 | 2455 | 1630 | [7] | South America* | Bayesian dispersal–vicariance analysis (S-DIVA) | [9] | 22.096 | 0.971 | 5.125 | 0.946 | 57.990 | 2.062 | 2 | [6], [3], Núñez L. (unpublished data) |
| Emeralds | *Microchera albocoronata* | 0 | 1 | 0 | 0 | [1, 2] | 14 | 8 | 6 | [3] | 300 | 1650 | 1350 | [2] | North America | Ancestral State Reconstruction | [8] |  |  | 2.550 | 0.071 | 2.535 | 0.015 | 2 | [6], Núñez L. (unpublished data) |
| Emeralds | *Orthorhyncus cristatus* | 0 | 0 | 1 | 0 | [1, 2] | 19 | 12 | 7 | [3] | 0 | 500 | 500 | [2] | South America | Ancestral State Reconstruction | [8] | 10.200 | 0.700 | 3.275 | 0.591 | 46.650 | 0.150 | 2 | [6], Núñez L. (unpublished data) |
| Emeralds | *Phaeochroa cuvierii* | 0 | 1 | 0 | 0 | [1, 2] | 18 | 7 | 11 | [3] | 0 | 350 | 350 | [7] | South America* | Ancestral State Reconstruction | [8] | 20.050 | 0.250 | 9.000 | 0.906 |  |  | 2 | [6], Núñez L. (unpublished data) |
| Emeralds | *Stephanoxis lalandi* | 0 | 1 | 0 | 0 | [1, 2] | -20 | -30 | 10 | [3] | 1150 | 2490 | 1340 | [7] | South America* | Ancestral State Reconstruction | [8] | 14.143 | 0.960 | 2.817 | 0.508 | 47.685 | 1.238 | 2 | [6], [3], Núñez L. (unpublished data) |
| Emeralds | *Taphrospilus hypostictus* | 0 | 1 | 0 | 0 | [1, 2] | -1 | -28 | 27 | [3] | 500 | 1200 | 700 | [7] | South America | Ancestral State Reconstruction | [8] | 22.498 | 0.918 | 7.000 | 0.308 | 63.568 | 1.303 | 3 | [6], [3] |
| Emeralds | *Thalurania colombica* | 0 | 1 | 0 | 0 | [1, 2] | 14 | 3 | 11 | [3] | 0 | 1900 | 1900 | [7] | South America | Ancestral State Reconstruction | [8] | 18.752 | 0.955 | 4.255 | 0.293 | 52.793 | 3.497 | 2 | [6], [3], Núñez L. (unpublished data) |
| Emeralds | *Thalurania fannyi* | 0 | 1 | 0 | 0 | [1, 2] | 11 | 3 | 8 | [10] | 0 | 1900 | 1900 | [2] | South America* | Ancestral State Reconstruction | [8] |  |  | 4.450 | 0.354 |  |  | 2 | [6] |
| Emeralds | *Thalurania furcata* | 0 | 1 | 0 | 0 | [1, 2] | 6 | -28 | 34 | [3] | 0 | 1900 | 1900 | [7] | South America | Ancestral State Reconstruction | [8] | 20.322 | 0.717 | 4.236 | 0.448 | 53.919 | 2.489 | 2 | [6], [3], Núñez L. (unpublished data) |
| Emeralds | *Thalurania glaucopis* | 0 | 1 | 0 | 0 | [1, 2] | -20 | -28 | 8 | [3] | 980 | 980 | 0 | [7] | South America* | Ancestral State Reconstruction | [8] | 18.607 | 0.933 | 4.500 | 0.548 | 54.587 | 3.159 | 2 | [6], [3], Núñez L. (unpublished data) |
| Emeralds | *Thalurania ridgwayi* | 1 | 1 | 0 | 0 | [1, 2] | 21 | 19 | 2 | [3] | 250 | 1200 | 950 | [7] | North America* | Ancestral State Reconstruction | [8] |  |  | 3.733 | 0.404 |  |  | 2 | [6] |
| Emeralds | *Thalurania watertonii* | 0 | 1 | 0 | 0 | [1, 2] | -3 | -8 | 5 | [3] | 0 | 500 | 500 | [2] | South America* | Ancestral State Reconstruction | [8] |  |  | 4.800 | 0.163 |  |  | 2 | [6], Núñez L. (unpublished data) |
| Emeralds | *Trochilus polytmus* | 0 | 0 | 1 | 0 | [1, 2] | 19 | 18 | 1 | [3] | 0 | 1000 | 1000 | [2] | Caribbean* | Ancestral State Reconstruction | [8] | 20.397 | 1.065 | 4.729 | 0.390 | 59.895 | 4.366 | 2 | [6], [3], Núñez L. (unpublished data) |
| Emeralds | *Trochilus scitulus* | 0 | 0 | 1 | 0 | [1, 2] | 18 | 17 | 1 | [10] | 0 | 1000 | 1000 | [2] | Caribbean* | Ancestral State Reconstruction | [8] |  |  | 4.800 | 0.707 |  |  | 2 | [6] |
| Hermits | *Anopetia gounellei* | 0 | 1 | 0 | 0 | [1, 2] | -5 | -10 | 5 | [3] | 455 | 485 | 30 | [7] | South America* | Ancestral State Reconstruction | [8] |  |  | 3.000 | 0.000 |  |  | 4 | [6] |
| Hermits | *Eutoxeres aquila* | 0 | 1 | 0 | 0 | [1, 2] | 9 | -4 | 13 | [3] | 0 | 2100 | 2100 | [7] | South America | Ancestral State Reconstruction | [8] | 27.300 | 0.080 | 10.467 | 1.595 | 84.515 | 2.965 | 5 | [6], Núñez L. (unpublished data) |
| Hermits | *Eutoxeres condamini* | 0 | 1 | 0 | 0 | [1, 2] | 2 | -17 | 19 | [3] | 0 | 700 | 700 | [7] | South America | Ancestral State Reconstruction | [8] | 27.400 | 0.500 | 10.250 | 1.475 |  |  | 5 | [6], Núñez L. (unpublished data) |
| Hermits | *Glaucis aeneus* | 0 | 1 | 0 | 0 | [1, 2] | 15 | -5 | 20 | [3] | 0 | 800 | 800 | [7] | North America | Ancestral State Reconstruction | [8] | 29.491 | 0.786 | 5.465 | 0.374 | 52.783 | 3.508 | 4 | [6], [3], Núñez L. (unpublished data) |
| Hermits | *Glaucis hirsutus* | 0 | 1 | 0 | 0 | [1, 2] | 10 | -26 | 36 | [3] | 0 | 1000 | 1000 | [7] | North America | Ancestral State Reconstruction | [8] | 28.478 | 1.854 | 6.235 | 0.624 | 58.820 | 3.720 | 4 | [6], [3], [11], Núñez L. (unpublished data) |
| Hermits | *Phaethornis anthophilus* | 0 | 1 | 0 | 0 | [1, 2] | 10 | 6 | 4 | [3] | 0 | 1200 | 1200 | [7] | South America | Ancestral State Reconstruction | [8] | 34.599 | 2.187 | 4.820 | 0.560 | 55.439 | 2.175 | 3 | [6], [3], Núñez L. (unpublished data) |
| Hermits | *Phaethornis atrimentalis* | 0 | 1 | 0 | 0 | [1, 2] | 1 | -5 | 6 | [3] | 0 | 1200 | 1200 | [2] | South America | Ancestral State Reconstruction | [8] | 24.191 | 0.959 | 2.572 | 0.146 | 38.973 | 1.134 | 3 | [6], [11], Núñez L. (unpublished data) |
| Hermits | *Phaethornis bourcieri* | 0 | 1 | 0 | 0 | [1, 2] | 8 | -2 | 10 | [3] | 0 | 400 | 400 | [7] | South America | Ancestral State Reconstruction | [8] | 28.690 | 1.567 | 4.119 | 0.488 | 54.465 | 2.579 | 2 | [6], [3], [11], Núñez L. (unpublished data) |
| Hermits | *Phaethornis eurynome* | 0 | 1 | 0 | 0 | [1, 2] | -20 | -28 | 8 | [3] | 76 | 2242 | 2166 | [7] | South America* | Ancestral State Reconstruction | [8] | 32.752 | 3.781 | 4.883 | 0.708 | 58.612 | 4.587 | 3 | [6], [3], Núñez L. (unpublished data) |
| Hermits | *Phaethornis guy* | 0 | 1 | 0 | 0 | [1, 2] | 11 | -18 | 29 | [3] | 0 | 2200 | 2200 | [2] | South America | Ancestral State Reconstruction | [8] | 41.343 | 2.360 | 5.421 | 1.275 | 60.683 | 1.941 | 4 | [6], [3], Núñez L. (unpublished data) |
| Hermits | *Phaethornis hispidus* | 0 | 1 | 0 | 0 | [1, 2] | 8 | -15 | 23 | [3] | 0 | 1000 | 1000 | [7] | South America | Ancestral State Reconstruction | [8] | 31.273 | 1.556 | 5.209 | 0.254 | 55.339 | 3.126 | 4 | [6], [3], [11], Núñez L. (unpublished data) |
| Hermits | *Phaethornis idaliae* | 0 | 1 | 0 | 0 | [1, 2] | -12 | -32 | 20 | [3] | 121 | 121 | 0 | [7] | South America* | Ancestral State Reconstruction | [8] |  |  | 2.250 | 0.354 |  |  | 4 | [6] |
| Hermits | *Phaethornis longirostris* | 1 | 1 | 0 | 0 | [1, 2] | 8 | -8 | 16 | [3] | 0 | 1000 | 1000 | [7] | North America | Ancestral State Reconstruction | [8] | 37.930 | 0.710 | 5.692 | 1.234 | 61.170 | 1.290 | 4 | [6], Núñez L. (unpublished data) |
| Hermits | *Phaethornis longuemareus* | 0 | 1 | 0 | 0 | [1, 2] | 21 | 0 | 21 | [3] | 0 | 1700 | 1700 | [7] | South America | Ancestral State Reconstruction | [8] | 21.673 | 0.858 | 3.039 | 0.577 | 38.275 | 2.215 | 4 | [6], [3], Núñez L. (unpublished data) |
| Hermits | *Phaethornis malaris* | 0 | 1 | 0 | 0 | [1, 2] | 8 | -20 | 28 | [3] | 0 | 600 | 600 | [2] | South America | Ancestral State Reconstruction | [8] | 38.003 | 2.483 | 5.935 | 0.458 | 59.129 | 2.706 | 4 | [6], [3], [11], Núñez L. (unpublished data) |
| Hermits | *Phaethornis nattereri* | 0 | 1 | 0 | 0 | [1, 2] | -16 | -20 | 4 | [3] | 0 | 500 | 500 | [2] | South America* | Ancestral State Reconstruction | [8] |  |  | 2.750 | 0.354 |  |  | 4 | [6] |
| Hermits | *Phaethornis pretrei* | 0 | 1 | 0 | 0 | [1, 2] | -18 | -20 | 2 | [3] | 364 | 1091 | 727 | [7] | South America* | Ancestral State Reconstruction | [8] | 30.252 | 1.108 | 4.200 | 0.251 | 57.478 | 2.105 | 3 | [6], [3], Núñez L. (unpublished data) |
| Hermits | *Phaethornis ruber* | 0 | 1 | 0 | 0 | [1, 2] | 8 | -24 | 32 | [3] | 0 | 500 | 500 | [7] | South America | Ancestral State Reconstruction | [8] | 22.053 | 1.404 | 2.625 | 0.558 | 33.309 | 4.383 | 3 | [6], [3], [11], Núñez L. (unpublished data) |
| Hermits | *Phaethornis striigularis* | 0 | 1 | 0 | 0 | [1, 2] | 18 | -1 | 19 | [3] | 0 | 1800 | 1800 | [7] | South America* | Ancestral State Reconstruction | [8] |  |  | 2.550 | 0.497 |  |  | 3 | [6], Núñez L. (unpublished data) |
| Hermits | *Phaethornis superciliosus* | 0 | 1 | 0 | 0 | [1, 2] | 22 | -2 | 24 | [3] | 0 | 1800 | 1800 | [7] | South America* | Ancestral State Reconstruction | [8] | 41.720 | 2.757 | 6.187 | 0.505 | 64.913 | 4.161 | 4 | [6], [3], Núñez L. (unpublished data) |
| Hermits | *Phaethornis syrmatophorus* | 0 | 1 | 0 | 0 | [1, 2] | 4 | -6 | 10 | [3] | 800 | 2400 | 1600 | [7] | South America | Ancestral State Reconstruction | [8] | 39.802 | 2.594 | 5.750 | 0.870 | 58.774 | 2.554 | 4 | [6], [3], Núñez L. (unpublished data) |
| Hermits | *Phaethornis yaruqui* | 0 | 1 | 0 | 0 | [1, 2] | 12 | 5 | 7 | [3] | 0 | 1500 | 1500 | [7] | South America | Ancestral State Reconstruction | [8] | 40.307 | 2.505 | 5.450 | 1.285 | 57.916 | 3.161 | 3 | [6], [3], Núñez L. (unpublished data) |
| Hermits | *Ramphodon naevius* | 0 | 1 | 0 | 0 | [1, 2] | -12 | -32 | 20 | [3] | 0 | 500 | 500 | [2] | South America* | Ancestral State Reconstruction | [8] | 33.125 | 1.536 | 7.100 | 1.327 | 68.668 | 4.351 | 3 | [6], [3], Núñez L. (unpublished data) |
| Hermits | *Threnetes leucurus* | 0 | 1 | 0 | 0 | [1, 2] | 5 | -23 | 28 | [3] | 0 | 1000 | 1000 | [7] | North America | Ancestral State Reconstruction | [8] | 27.775 | 1.683 | 5.262 | 0.761 | 58.422 | 3.434 | 3 | [6], [3], [11], Núñez L. (unpublished data) |
| Hermits | *Threnetes niger* | 0 | 1 | 0 | 0 | [1, 2] | 5 | 2 | 3 | [3] | 0 | 500 | 500 | [2] | South America* | Ancestral State Reconstruction | [8] | 28.471 | 1.074 | 5.875 | 1.031 | 59.114 | 3.439 | 3 | [6], [3] |
| Hermits | *Threnetes ruckeri* | 0 | 1 | 0 | 0 | [1, 2] | 16 | -5 | 21 | [3] | 0 | 1050 | 1050 | [7] | North America | Ancestral State Reconstruction | [8] | 28.628 | 0.701 | 6.047 | 0.419 | 55.565 | 4.095 | 3 | [6], [3], Núñez L. (unpublished data) |
| Mangoes | *Androdon aequatorialis* | 0 | 1 | 0 | 0 | [1, 2] | 10 | -5 | 15 | [3] | 0 | 1590 | 1590 | [7] | South America | Ancestral State Reconstruction | [8] | 37.968 | 1.862 | 7.460 | 1.091 | 65.165 | 3.099 | 1 | [6], [3], Núñez L. (unpublished data) |
| Mangoes | *Anthracothorax dominicus* | 0 | 0 | 1 | 0 | [1, 2] | 20 | 18 | 2 | [3] | 0 | 1500 | 1500 | [2] | Caribbean* | Ancestral State Reconstruction | [8] |  |  | 6.250 | 1.708 |  |  | 3 | [6] |
| Mangoes | *Anthracothorax mango* | 0 | 0 | 1 | 0 | [1, 2] | 19 | 18 | 1 | [3] | 900 | 1500 | 600 | [2] | Caribbean | Ancestral State Reconstruction | [8] |  |  | 8.500 |  |  |  | 3 | [6] |
| Mangoes | *Anthracothorax nigricollis* | 0 | 1 | 0 | 0 | [1, 2] | 10 | -28 | 38 | [3] | 0 | 1750 | 1750 | [7] | North America | Ancestral State Reconstruction | [8] | 23.450 | 0.750 | 6.940 | 0.377 | 65.295 | 1.025 | 3 | [6], Núñez L. (unpublished data) |
| Mangoes | *Anthracothorax prevostii* | 1 | 1 | 0 | 0 | [1, 2] | 22 | -4 | 26 | [3] | 0 | 1500 | 1500 | [7] | North America* | Ancestral State Reconstruction | [8] | 26.418 | 0.573 | 6.633 | 0.404 | 66.765 | 7.284 | 3 | [6], [3], Núñez L. (unpublished data) |
| Mangoes | *Anthracothorax veraguensis* | 0 | 1 | 0 | 0 | [1, 2] | 10 | 7 | 3 | [3] | 0 | 700 | 700 | [7] | North America* | Ancestral State Reconstruction | [8] |  |  | 7.000 |  |  |  | 3 | [6] |
| Mangoes | *Anthracothorax viridigula* | 0 | 1 | 0 | 0 | [1, 2] | 9 | 2 | 7 | [3] | 0 | 500 | 500 | [2] | South America* | Ancestral State Reconstruction | [8] |  |  | 7.200 | 1.151 |  |  | 3 | [6], Núñez L. (unpublished data) |
| Mangoes | *Anthracothorax viridis* | 0 | 0 | 1 | 0 | [1, 2] | 19 | 18 | 1 | [3] | 800 | 1200 | 400 | [2] | Caribbean* | Ancestral State Reconstruction | [8] | 24.465 | 1.238 | 7.000 |  | 61.298 | 0.987 | 3 | [6], [3] |
| Mangoes | *Augastes lumachella* | 0 | 1 | 0 | 0 | [1, 2] | -18 | -20 | 2 | [3] | 950 | 1600 | 650 | [7] | South America* | Ancestral State Reconstruction | [8] |  |  | 4.400 | 0.566 |  |  | 2 | [6] |
| Mangoes | *Augastes scutatus* | 0 | 1 | 0 | 0 | [1, 2] | -18 | -20 | 2 | [3] | 1000 | 2000 | 1000 | [7] | South America* | Ancestral State Reconstruction | [8] |  |  | 4.250 | 1.061 |  |  | 2 | [6] |
| Mangoes | *Avocettula recurvirostris* | 0 | 1 | 0 | 0 | [1, 2] | 8 | 0 | 8 | [3] | 0 | 800 | 800 | [2] | South America* | Ancestral State Reconstruction | [8] | 16.685 | 0.055 | 4.250 | 0.071 | 57.950 | 0.030 | 1 | [6], [3] |
| Mangoes | *Chrysolampis mosquitus* | 0 | 1 | 0 | 0 | [1, 2] | 11 | -24 | 35 | [3] | 0 | 1740 | 1740 | [7] | South America | Ancestral State Reconstruction | [8] | 11.685 | 0.935 | 4.035 | 0.676 | 52.640 | 1.090 | 2 | [6], Núñez L. (unpublished data) |
| Mangoes | *Colibri coruscans* | 0 | 1 | 0 | 1 | [1, 2] | 8 | -28 | 36 | [3] | 600 | 3600 | 3000 | [7] | South America | Ancestral State Reconstruction | [8] | 22.625 | 1.205 | 7.475 | 0.726 | 72.885 | 3.815 | 2 | [6], Núñez L. (unpublished data) |
| Mangoes | *Colibri delphinae* | 0 | 1 | 0 | 0 | [1, 2] | 16 | -1 | 17 | [3] | 300 | 2800 | 2500 | [7] | North America | Ancestral State Reconstruction | [8] | 17.350 | 1.050 | 6.375 | 0.854 | 68.700 | 1.900 | 2 | [6], Núñez L. (unpublished data) |
| Mangoes | *Colibri serrirostris* | 0 | 1 | 0 | 0 | [1, 2] | -16 | -34 | 18 | [3] | 2075 | 2075 | 0 | [7] | South America* | Ancestral State Reconstruction | [8] | 21.236 | 1.402 | 6.200 | 0.490 | 68.098 | 4.367 | 2 | [6], [3], Núñez L. (unpublished data) |
| Mangoes | *Colibri thalassinus* | 1 | 1 | 0 | 0 | [1, 2] | 32 | -27 | 59 | [3] | 600 | 3350 | 2750 | [7] | North America | Ancestral State Reconstruction | [8] | 20.833 | 1.461 | 4.772 | 0.647 | 62.836 | 3.192 | 3 | [6], [3], Núñez L. (unpublished data) |
| Mangoes | *Doryfera johannae* | 0 | 1 | 0 | 0 | [1, 2] | 8 | -12 | 20 | [3] | 280 | 1800 | 1520 | [7] | South America | Ancestral State Reconstruction | [8] | 27.443 | 2.155 | 4.653 | 1.019 | 53.024 | 3.950 | 1 | [6], [3], Núñez L. (unpublished data) |
| Mangoes | *Doryfera ludovicae* | 0 | 1 | 0 | 0 | [1, 2] | 11 | -17 | 28 | [3] | 900 | 2700 | 1800 | [7] | South America | Ancestral State Reconstruction | [8] | 32.477 | 1.821 | 5.875 | 0.604 | 57.040 | 1.943 | 1 | [6], [3], Núñez L. (unpublished data) |
| Mangoes | *Eulampis holosericeus* | 0 | 0 | 1 | 0 | [1, 2] | 23 | 12 | 11 | [3] | 0 | 500 | 500 | [2] | Caribbean | Ancestral State Reconstruction | [8] |  |  | 5.975 | 1.068 |  |  | 3 | [6], Núñez L. (unpublished data) |
| Mangoes | *Eulampis jugularis* | 0 | 0 | 1 | 0 | [1, 2] | 19 | 12 | 7 | [3] | 800 | 1200 | 400 | [2] | Caribbean | Ancestral State Reconstruction | [8] | 23.200 | 3.400 | 9.300 | 1.760 | 72.600 | 3.000 | 4 | [6], Núñez L. (unpublished data) |
| Mangoes | *Heliactin bilophus* | 0 | 1 | 0 | 0 | [1, 2] | 2 | -24 | 26 | [3] | 0 | 500 | 500 | [2] | South America | Ancestral State Reconstruction | [8] |  |  | 2.150 | 0.286 |  |  | 2 | [6], Núñez L. (unpublished data) |
| Mangoes | *Heliothryx auritus* | 0 | 1 | 0 | 0 | [1, 2] | 5 | -22 | 27 | [3] | 0 | 400 | 400 | [2] | South America* | Ancestral State Reconstruction | [8] |  |  | 5.500 | 0.546 |  |  | 2 | [6], Núñez L. (unpublished data) |
| Mangoes | *Heliothryx barroti* | 0 | 1 | 0 | 0 | [1, 2] | 18 | 0 | 18 | [3] | 0 | 1830 | 1830 | [7] | South America | Ancestral State Reconstruction | [8] | 16.755 | 0.105 | 5.463 | 0.315 | 74.385 | 0.415 | 2 | [6], Núñez L. (unpublished data) |
| Mangoes | *Polytmus guainumbi* | 0 | 1 | 0 | 0 | [1, 2] | 9 | -18 | 27 | [3] | 0 | 600 | 600 | [7] | South America | Ancestral State Reconstruction | [8] | 21.848 | 4.391 | 4.883 | 0.356 | 59.433 | 3.244 | 3 | [6], [3], Núñez L. (unpublished data) |
| Mangoes | *Polytmus milleri* | 0 | 1 | 0 | 0 | [1, 2] | 6 | 6 | 0.2 | [3] | 1300 | 2200 | 900 | [7] | South America* | Ancestral State Reconstruction | [8] | 2.500 | 0.000 | 5.000 | 0.829 |  |  | 3 | [6] |
| Mangoes | *Polytmus theresiae* | 0 | 1 | 0 | 0 | [1, 2] | 5 | -12 | 17 | [3] | 0 | 300 | 300 | [7] | South America | Ancestral State Reconstruction | [8] | 17.769 | 4.865 | 3.575 | 0.218 | 54.935 | 1.558 | 3 | [6], [3], Núñez L. (unpublished data) |
| Mangoes | *Schistes geoffroyi* | 0 | 1 | 0 | 0 | [1, 2] | 10 | -18 | 28 | [3] | 1400 | 2500 | 1100 | [7] | South America | Ancestral State Reconstruction | [8] |  |  | 3.800 | 0.424 |  |  | 2 | [6] |
| Mountain Gems | *Eugenes fulgens* | 1 | 1 | 0 | 0 | [1, 2] | 33 | 8 | 25 | [3] | 900 | 3300 | 2400 | [7] | North America | Ancestral State Reconstruction | [5] | 29.791 | 6.885 | 7.078 | 0.683 | 69.006 | 4.452 | 2 | [6], [3], Núñez L. (unpublished data) |
| Mountain Gems | *Heliomaster constantii* | 1 | 1 | 0 | 0 | [1, 2] | 27 | 10 | 17 | [3] | 0 | 1500 | 1500 | [7] | North America | Ancestral State Reconstruction | [5] | 34.512 | 1.298 | 7.168 | 0.509 | 63.136 | 1.579 | 2 | [6], [3], Núñez L. (unpublished data) |
| Mountain Gems | *Heliomaster furcifer* | 0 | 1 | 0 | 0 | [1, 2] | -16 | -36 | 20 | [3] | 0 | 1500 | 1500 | [7] | South America | Ancestral State Reconstruction | [5] |  |  | 5.750 | 0.612 |  |  | 3 | [6], Núñez L. (unpublished data) |
| Mountain Gems | *Heliomaster longirostris* | 1 | 1 | 0 | 0 | [1, 2] | 18 | -24 | 42 | [3] | 0 | 1525 | 1525 | [7] | North America | Ancestral State Reconstruction | [5] | 34.533 | 0.464 | 6.380 | 0.708 | 60.617 | 1.562 | 2 | [6], [3], Núñez L. (unpublished data) |
| Mountain Gems | *Heliomaster squamosus* | 0 | 1 | 0 | 0 | [1, 2] | -10 | -24 | 14 | [3] | 0 | 800 | 800 | [2] | South America | Ancestral State Reconstruction | [5] |  |  | 5.750 | 0.612 |  |  | 2 | [6], Núñez L. (unpublished data) |
| Mountain Gems | *Hylonympha macrocerca* | 0 | 1 | 0 | 0 | [1, 2] | 11 | 10 | 1 | [3] | 900 | 1200 | 300 | [7] | South America* | Ancestral State Reconstruction | [8] |  |  | 7.250 | 0.645 |  |  | 3 | [6] |
| Mountain Gems | *Lampornis amethystinus* | 1 | 1 | 0 | 0 | [1, 2] | 22 | 15 | 7 | [3] | 900 | 3050 | 2150 | [7] | North America | Ancestral State Reconstruction | [5] | 21.980 | 1.667 | 5.863 | 0.827 | 65.245 | 4.073 | 3 | [6], [3], Núñez L. (unpublished data) |
| Mountain Gems | *Lampornis calolaemus* | 0 | 1 | 0 | 0 | [1, 2] | 11 | 8 | 3 | [10] | 800 | 2500 | 1700 | [2] | North America | Ancestral State Reconstruction | [5] |  |  | 5.425 | 0.660 |  |  | 2 | [6] |
| Mountain Gems | *Lampornis castaneoventris* | 0 | 1 | 0 | 0 | [1, 2] | 11 | 7 | 4 | [3] | 1220 | 3050 | 1830 | [7] | North America | Ancestral State Reconstruction | [8] | 21.800 | 0.500 | 5.425 | 0.610 | 62.150 | 3.650 | 2 | [6], Núñez L. (unpublished data) |
| Mountain Gems | *Lampornis clemenciae* | 1 | 1 | 0 | 0 | [1, 2] | 32 | 17 | 15 | [3] | 300 | 3900 | 3600 | [7] | North America | Ancestral State Reconstruction | [5] | 26.225 | 2.519 | 7.193 | 1.218 | 72.492 | 4.680 | 3 | [6], [3], Núñez L. (unpublished data) |
| Mountain Gems | *Lampornis hemileucus* | 0 | 1 | 0 | 0 | [1, 2] | 11 | 7 | 4 | [3] | 700 | 1400 | 700 | [2] | North America | Ancestral State Reconstruction | [5] | 20.750 | 0.650 | 5.650 | 0.635 | 61.350 | 3.050 | 2 | [6], Núñez L. (unpublished data) |
| Mountain Gems | *Lampornis viridipallens* | 0 | 1 | 0 | 0 | [1, 2] | 15 | 13 | 2 | [3] | 900 | 2200 | 1300 | [7] | North America | Ancestral State Reconstruction | [5] | 20.609 | 0.885 | 5.349 | 1.331 | 63.232 | 4.167 | 2 | [6], [3], Núñez L. (unpublished data) |
| Mountain Gems | *Lamprolaima rhami* | 1 | 1 | 0 | 0 | [1, 2] | 18 | 14 | 4 | [3] | 900 | 2950 | 2050 | [7] | North America | Ancestral State Reconstruction | [5] | 16.800 | 0.000 | 6.900 | 1.212 | 72.000 | 0.000 | 2 | [6], [3] |
| Mountain Gems | *Panterpe insignis* | 0 | 1 | 0 | 0 | [1, 2] | 11 | 7 | 4 | [3] | 1800 | 3000 | 1200 | [7] | North America | Ancestral State Reconstruction | [5] |  |  | 5.558 | 0.592 |  |  | 2 | [6], Núñez L. (unpublished data) |
| Mountain Gems | *Sternoclyta cyanopectus* | 0 | 1 | 0 | 0 | [1, 2] | 11 | 8 | 3 | [3] | 0 | 1900 | 1900 | [7] | South America | Morphology | [12] |  |  | 9.275 | 0.797 |  |  | 3 | [6] |
| Patagona | *Patagona gigas* | 0 | 1 | 0 | 1 | [1, 2] | 0 | -35 | 35 | [3] | 0 | 3660 | 3660 | [7] | South America* | Ancestral State Reconstruction | [8] |  |  | 20.567 | 2.272 |  |  | 2 | [6] |
| Topazes | *Florisuga fusca* | 0 | 1 | 0 | 0 | [1, 2] | -8 | -32 | 24 | [3] | 0 | 1400 | 1400 | [2] | South America* | Ancestral State Reconstruction | [8] |  |  | 8.000 | 1.414 |  |  | 3 | [6] |
| Topazes | *Florisuga mellivora* | 0 | 1 | 0 | 0 | [1, 2] | 18 | -20 | 2 | [3] | 0 | 1600 | 1600 | [7] | South America | Ancestral State Reconstruction | [8] | 18.310 | 0.430 | 6.850 | 0.444 | 75.440 | 2.680 | 3 | [6], Núñez L. (unpublished data) |
| Topazes | *Topaza pella* | 0 | 1 | 0 | 0 | [1, 2] | 0 | -2 | 2 | [3] | 250 | 500 | 250 | [7] | South America | Ancestral State Reconstruction | [8] | 4.300 | 0.000 | 11.752 | 2.075 |  |  | 3 | [6], Núñez L. (unpublished data) |

SUPPLEMENTARY REFERENCES

1. Sullivan BL, Wood CL, Iliff MJ, Bonney RE, Fink D, Kelling S. eBird: A citizen-based bird observation network in the biological sciences. Biological Conservation. 2009;142(10):2282-92. doi: <https://doi.org/10.1016/j.biocon.2009.05.006>.

2. del Hoyo J, Elliott A, Sargatal J, Christie D, de Juana E. Handbook of the Birds of the World Alive Barcelona, Spain: Lynx Edicions; 2017. Available from: <http://www.hbw.com/>.

3. Ornelas JF. Radiation in the genus *Amazilia*: A comparative approach to undestanding the diversification of Hummingbirds. Phoenix: The University of Arizona; 1995.

4. Arizmendi MC, Berlanga H. Colibríes de México y Norteamérica. Hummingbirds of Mexico and North America. Mexico: CONABIO; 2014. 160 p.

5. Licona-Vera Y, Ornelas JF. The conquering of North America: dated phylogenetic and biogeographic inference of migratory behavior in bee hummingbirds. BMC Evolutionary Biology. 2017;17(1):126. doi: 10.1186/s12862-017-0980-5.

6. Schuchmann KL. Family Trochilidae (Hummingbirds). In: del Hoyo J, Elliott A, Sargatal J, editors. Handbook of the birds of the world. Volume 5: Barn-owls to hummingbirds. Barcelona: Lynx Editions; 1999. p. 468–680.

7. Bleiweiss R. Ecological Causes of Clade Diversity in Hummigbirds: A Neontological Perspective on the Generation of Diversity. In: Ross RM, Allmon WD, editors. Causes of Evolution A Paleontological Perspective. Chicago: The University of Chicago Press; 1990. p. 354-80.

8. McGuire JA, Witt CC, Altshuler DL, Remsen JV. Phylogenetic Systematics and Biogeography of Hummingbirds: Bayesian and Maximum Likelihood Analyses of Partitioned Data and Selection of an Appropriate Partitioning Strategy. Syst Biol. 2007;56(5):837-56. doi: 10.1080/10635150701656360.

9. Ornelas JF, González C, de los Monteros AE, Rodríguez-Gómez F, García-Feria LM. In and out of Mesoamerica: temporal divergence of Amazilia hummingbirds pre-dates the orthodox account of the completion of the Isthmus of Panama. Journal of Biogeography. 2014;41(1):168-81. doi: 10.1111/jbi.12184.

10. NatureServe. Infonatura. Animales y Ecosistemas de América Latina Arlington, Virginia, USA2007. Available from: <http://infonatura.natureserve.org/>.

11. Rodríguez-Flores C. Organización de la comunidad de colibríes ermitaños (Trochilidae: Phaethorninae) y sus flores en bosques de tierra firme del Parque Nacional Natural Amacayacu (Amazonas, Colombia). . Bogotá: Universidad Nacional de Colombia; 2002.

12. Renner SC, Schuchmann K-L-. Biogeography, geographical Variation, and taxonomy of the hummingbird genera Eugenes Gould, 1856, Sternoclyta Gould, 1858, and Hylonympha Gould, 1873 (Aves: Trochilidae). Ornithologischer Anzeiger. 2004;43:103-14.
