# Supplementary material for: Are hummingbirds generalists or specialists? Using network analysis to explore the mechanisms influencing their interaction with nectar resources
Source: PLoS One. 2019 Feb 27;14(2):e0211855. doi: 10.1371/journal.pone.0211855 (PMC6392410; doi:10.1371/journal.pone.0211855)
Supplement: S1 Appendix — Results for the degree distribution, nestedness, and modularity analyses of the mutualistic network of hummingbird clades and their nectar plants. In this network, we included all records independently of plant origin (native or exotic to the American continent). (DOCX) [file pone.0211855.s007.docx]

**S1 Appendix**. **Network analysis of the matrix including native and exotic plant species.** Results for the degree distribution, nestedness, and modularity analyses of the mutualistic network of hummingbird clades and their nectar plants. In this network, we included all records independently of plant origin (native or exotic to the American continent).


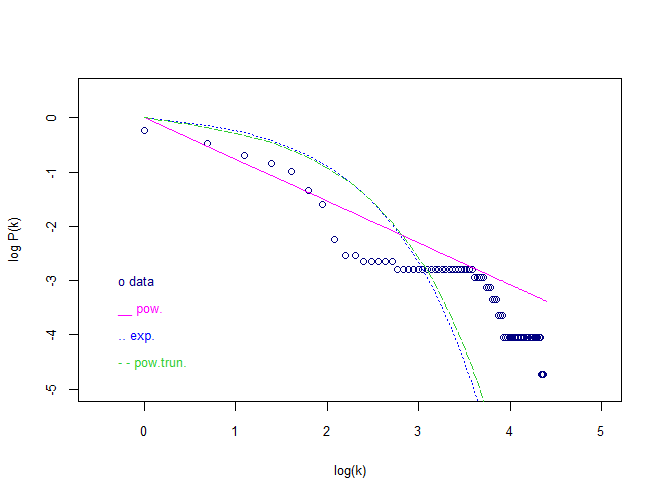


This graph shows the cumulative frequency distribution (P (*k*)) of the number of links (*k*) for the network between hummingbirds and their floral nectar resources. The graph in a log-log scale combines plant and hummingbird interactions. The original data (circles) was adjusted to three distributions: (1) power-law function (pow.), (2) exponential (exp.), and (3) truncated power-law (pow.trun.). The hummingbird-plant interaction network has a better fit to the power-law function (AIC exp. = 678.193, AIC pow. = 586.747, AIC pow.trun. = 676.967).

When the nodes are ordered based on the connection number, this net was highly nested (NODF = 72.160), and hummingbirds and plants got high NODF value (NODF hummingbirds = 82.320, NODF plants = 72.090). Compared with the null models, this matrix was statistically different from a randomly selected network using ER and CE models (NODF model ER = 44.220, *P* < 0.001, SES model ER = 15.022; NODF model CE = 52.260, *P* < 0.001, SES model CE = 10.001), but not using FF model (NODF model FF = 71.500, *P* = 0.938, SES model FF = 1.803).

When phylogeny was accounted for, the nestedness of the network diminished (NODF = 42.600, NODF hummingbirds = 64.650, NODF plants = 42.450). Nonetheless, the network remained statistically different from randomly selected networks with ER and CC models (NODF model ER = 22.120, *P* < 0.001, SES model ER = 9.356; NODF model CE = 29.170, *P* < 0.001, SES model CE = 6.693), but not from networks with FF model (NODF model FF = 40.487, *P* = 0.873, SES model FF = 5.579).

Respect modularity, the network were not significantly modular (M = 0.183 ± 0.002, M model ER = 0.222 (*P* = 0.944, SES model ER = -7.655), M model FF = 0.210 (*P* = 0.953, SES model FF = -5.657).
